# Synthesis and Biological Screening of Structurally Modified Phaeosphaeride Analogues

**DOI:** 10.3390/molecules30092016

**Published:** 2025-04-30

**Authors:** Konstantinos Rantzios, Oraia-Eirini Chatzimentor, George Leonidis, Jorgo Giuliani, Ioanna Sigala, Vasiliki Sarli

**Affiliations:** Department of Chemistry, Aristotle University of Thessaloniki, University Campus, 54124 Thessaloniki, Greece

**Keywords:** phaeosphaeride, tetronate, tetramate, natural product analogues, anticancer

## Abstract

Phaeosphaeride A and its analogues have been extensively explored for their potential pharmacological applications, particularly in the development of anticancer agents. In this study, the synthesis of structurally modified phaeosphaeride analogues is reported. The structures of the synthesized analogues bearing the tetrahydro- and hexahydro-2*H*-furo[3,2-*b*]pyran-2-one and hexahydropyrano[3,2-*b*]pyrrol-2(1*H*)-one moieties were assessed and the new compounds were evaluated for their antiproliferative activity against two cancer cell lines. Despite successful synthesis and structural modification, the majority of the phaeosphaeride analogues exhibited limited bioactivity. Structure-activity relationship studies suggested that specific modifications did not enhance anticancer potency. The hydroxy groups and the alkyl moiety in cyclic or non-cyclic phaeosphaeride analogues contribute to the activity, as shown by the activity of compounds **24** and **25**. The presence of double bonds and oxygen or nitrogen heteroatoms in furopyranones or pyranopyrrolones **9**, **28**, **29** and **33a**, does not significantly impact cytotoxic activity. These findings highlight the challenges in optimizing phaeosphaerides for anticancer applications and provide insights into future structural modifications to improve their therapeutic potential. Moreover, our studies open a synthetic route for the development of new phaeosphaeride analogues.

## 1. Introduction

Natural products have been a rich source of inspiration for drug discovery since ancient times [1]. Nowadays, numerous studies demonstrate the efficacy of natural products, their derivatives and synthetic analogues in targeting biological pathways and treating or preventing human diseases such as cancer, infections, cardiovascular diseases and others [2,3]. Natural product drug discovery is often a complicated process due to the structural complexity of natural products, requiring multistep, expensive, and time-consuming synthesis. In response to current drug discovery needs, natural product synthesis and the development of natural product-based chemical libraries remain at the forefront of pharmaceutical research [4,5,6].

**Figure 1:**
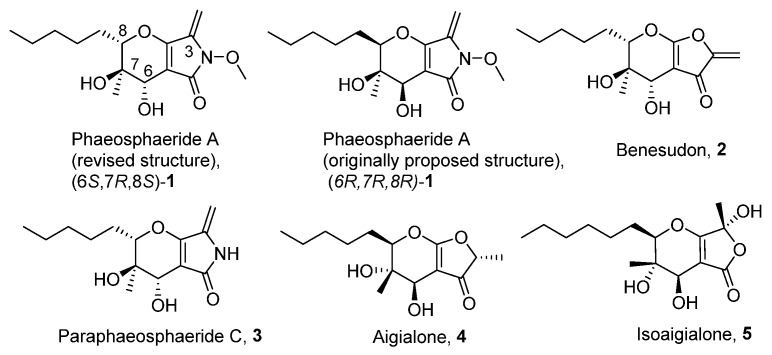
Phaeosphaerides and other structural related natural products.

In 2006 phaeosphaerides A and B were isolated by an endophytic fungus from the genus Phaeosphaeria [7]. Phaeosphaeride A was found to inhibit the Signal Transducer and Activator of Transcription 3 (STAT3) signalling pathway and display promising anticancer activities. In the following years, phaeosphaerides have attracted the attention of our group and Kobayashi’s as synthetic targets [8,9,10]. Initial efforts were focused to structural elucidation and determination of the mode of action of phaeosphaerides. Subsequent biological studies and SAR investigation by Berestetskiy et al. pointed out the herbicidal potential of phaeosphaeride A [11], while Abzianidze et al. studied the anticancer activities of synthetic phaeosphaeride derivatives [12,13]. Since phaeosphaerides [14], paraphaeosphaerides **3** [15], aigialone **4** [16], isoaigialone **5** [15] and related compounds [17] are biologically active natural products, we aimed to synthesize hydrofuropyrans and hydropyranopyrrolones as synthetic analogues of phaeosphaerides and study their antiproliferative properties (Figure 1).

**Figure 2:**
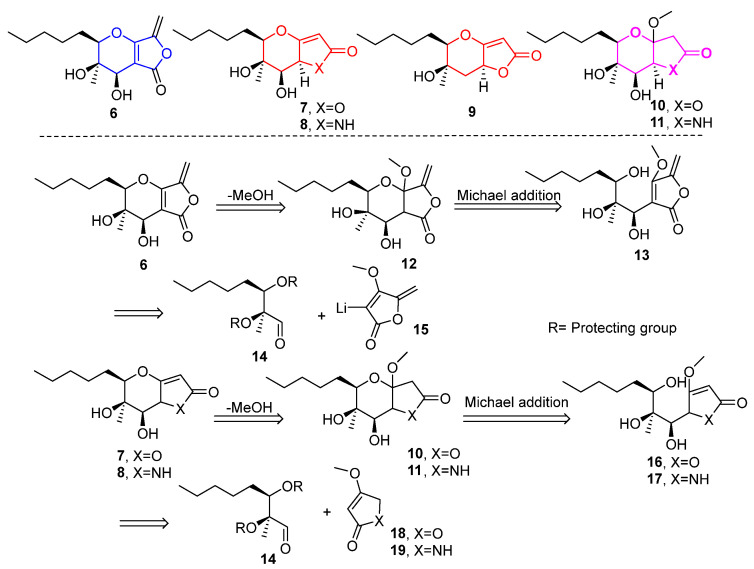
Retrosynthetic disconnections for phaeosphaeride analogues. Lactone **6** and analogues **7**–**11** were selected as synthetic targets in this work.

Our first plan included efforts to synthesize **6**, containing the tetrahydro-5*H*-furo[3,4-*b*]pyran-5-one scaffold, which is naturally occurring. The same protocol could then be applied to the synthesis of tetrahydro- and hexahydro-2*H*-furo[3,2-*b*]pyran-2-one **7**, **9, 10** and tetrahydro- and hexahydropyrano[3,2-*b*]pyrrol-2(1*H*)-ones **8**, **11**. The synthetic route would be similar to that initially reported by our group during the total synthesis of the proposed structure of phaeosphaeride A, (6*R*,7*R*,8*R*)-**1**, with significant differences after the aldehyde synthesis stage [9]. Herein, key synthetic steps were designed to include the addition of lithiated methyl tetronate **15** to **14**, the vinylogous aldol reaction of **18**, **19** to **14**, deprotection of the hydroxy protecting groups and intramolecular *oxy*-Michael reactions for the formation of the 3,4-dihydro-*2H*-pyran (Figure 2).

## 2. Results

### 2.1. Synthesis of Phaeosphaeride Analogues

**Scheme 1:**
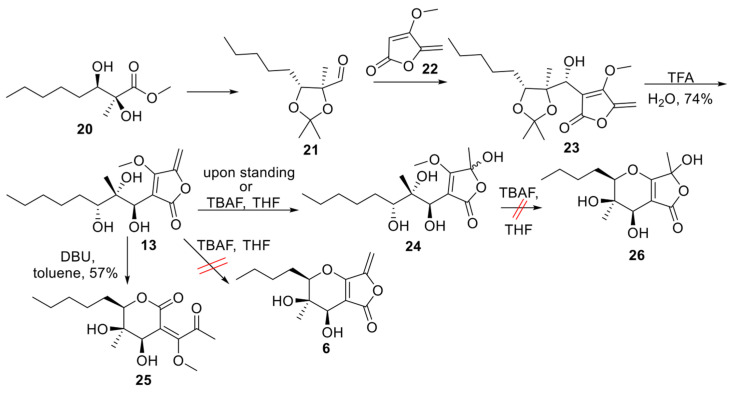
Cyclisation of tetronate **13** [18].

Aldehyde **21** (Scheme 1) represents a versatile chiral intermediate to access phaeosphaeride analogues. According to our previous work, the synthesis of compound **21** involved the addition of vinyl lithium reagent of **22** to the acetonide-protected aldehyde **21** following the acetonide group deprotection using TFA. Although compound **13** was stable enough to be purified by column chromatography, upon standing, it was hydrated to compound **24**. Therefore, **13** was advanced to the next step immediately after its purification. Subsequently, intramolecular oxy-Michael addition using various reagents and conditions was attempted. Initially, 1.5 equivalent of TBAF (1.6M) in THF was used at 60 °C following the previously described procedure for the Michael reaction in phaeosphaeride derivatives synthesis (Table 1). However, the hydration of the exocyclic double bond took place and the formation of isomer **24** (20% yield) was again observed together with a complicated reaction mixture containing *retro* aldol reaction products. In a second attempt, a different base DBU was selected instead of TBAF. The reaction was carried out using 1.5 equivalents of DBU in toluene at rt and stirring overnight. In this case, the six-membered lactone **25** formation took place and the desired product **6** could not be detected. On the other hand, the cyclisation of **13** in acidic conditions using *p*-TSA resulted in complicated mixtures. Our experiments indicate that the intramolecular Michael reaction is challenging to perform, and tetronate **13** preferably produces lactone **25**.

**Figure 3:**
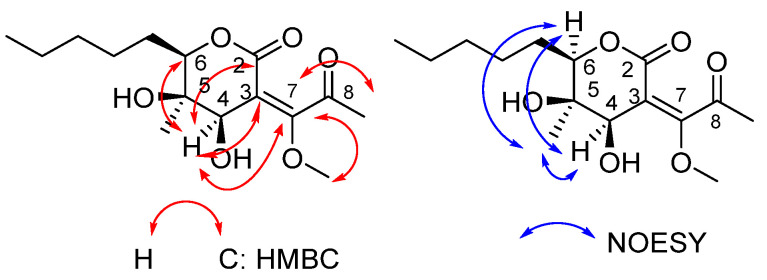
Key HMBC and NOESY and correlations of compound **25**.

The two-dimensional HMBC and NOESY correlations, as well as the final structure assigned to product **25**, are shown in Figure 3. The two-dimensional spectra demonstrate the characteristic correlation between the C-7 of the double bond and H atoms of the methoxy group. HMBC correlations of H-4 /C-2 and C-7 indicated the formation of the tetrahydro-2*H*-pyran-2-one core. The chemical shift in the ketone carbonyl carbon at 198.3 ppm is highly diagnostic of the structure of **25**. The assigned structure was further supported by detailed analysis of the 1D and 2D NMR data (Table 2).

**Scheme 2:**
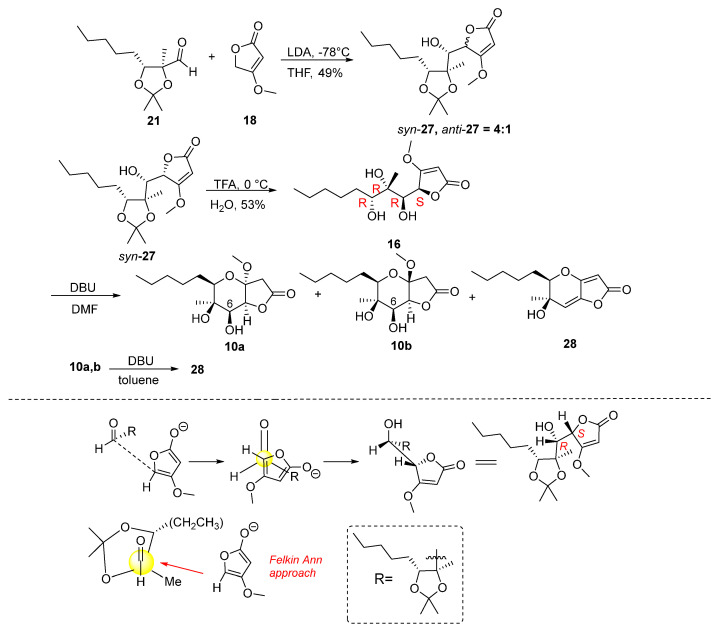
Synthesis of *γ*-butyrolactones **10a,b** and butenolide **28**.

Attention was then turned to the synthesis of analogues **7** and **9** (Figure 2). Specifically, the synthesis started via a reaction between aldehyde **21** and the lithium derivative of 4-methoxyfuran-2(5*H*)-one **18**. The reaction was carried out using LDA in THF solvent at −78 °C for two hours (Scheme 2), which resulted in the formation of butenolides **27** in 49% yield and in a ratio of *syn*:*anti* = 4:1. We were able to isolate the major isomer *syn*-**27**, the stereochemistry of which was determined at a later stage. The addition of 4-methoxyfuran-2(5*H*)-one to **21** furnishes mainly the Felkin–Anh product *syn*-**27** as shown in Scheme 2. However, it should not be excluded that butanolide *syn*-**27** is the thermodynamic stable product resulting from the epimerization of the butenolide stereogenic center under basic conditions as previously reported by Karak et al. [19].

Having *syn*-**27** in hand, the synthetic course proceeded to the deprotection step using TFA (Scheme 2). Butenolide **16** was obtained in 53% yield and was then stirred under basic conditions to form the tetrahydropyran ring (Table 3). The reaction was carried out with 1.5 equiv. of DBU in DMF solvent at 60 °C for 72 h, which yielded a mixture of two cyclization product isomers **10a**,**b** in a 4:1 ratio and in 69% yield. Their complete separation was not possible by column chromatography in various solvents. However, we were able to isolate and fully characterize a small amount of the minor isomer **10b**, with a purity of 70% relative to the major one **10a**. Along with the mixture of isomers, the reaction also resulted in the formation of product **28** in 39%. Compound **28** was formed from MeOH elimination and dehydration of alcohols **10a**,**b**.

**Figure 4:**
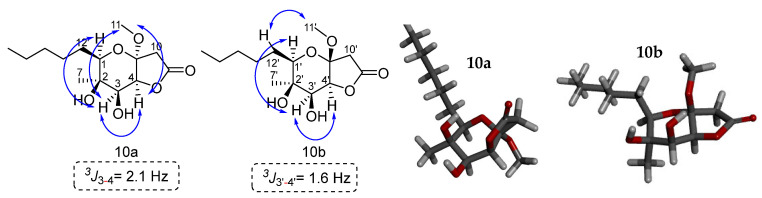
Key NOESY correlations of lactones **10a** and **10b**. The modelling of compounds **10a** and **10b** was performed with the MM2 method molecular energy calculations using Chem3D 15.0 software. BIOVIA Discovery Studio was used for the visualization of the 3D model.

Our next efforts were focused on the structure determination of **10b**. It was possible to isolate also a small amount of minor isomer **10b** to assign its stereochemistry. The sample had a purity of 70% of **10b** and 30% of **10a**. The two-dimensional NOESY spectrum shows key correlations between the C-1′ hydrogen and the C-3′ hydrogen. Additionally, a correlation is observed between the C-3′ and C-4′ hydrogens, suggesting the *syn* arrangement between them. The coupling constant ^3^J_3′-4′_ = 1.6 Hz confirms this stereochemistry. For **10b** the methoxy group shows a correlation of its hydrogens with the C-12′ hydrogens. For **10a** the stereochemistry for the methoxy group could be determined by the association of its hydrogens with the C-3 and C-4 hydrogens. Another sample that was enriched with **10a** (**10a**:**10b** = 4:1) was used for the 2D NMR experiments. NOESY correlations for **10a** indicated a *syn* relationship between H-1, H-3, CH_3_O and H-4 on the dihydropyran ring. This is verified by the coupling constant ^3^J_3-4_ of 2.1 Hz between H-3 and H-4. Based on these results we can conclude that the relative stereochemistry of the two isomers is this shown in Figure 4.

To improve the cyclization reaction of **16**, it was carried out under the same conditions changing the time from 72 to 24 h (Table 2). It was observed that in a shorter reaction time, the mixture of isomers was formed in a higher yield of 74%, while **28** was isolated in a lower yield (12%). At the same time, an attempt was made to form compound **28** without obtaining the mixture of isomers. Thus, compounds **10a,b** reacted with three equivalents of DBU in DMF with at 80 °C for 48 h. From the reaction both the mixture of isomers **10a,b** and compound **28** were obtained in 46% yield and 47% yield, respectively. In conclusion, the formation of **28** is favoured with an increase in base equivalents, temperature and reaction time. In another attempt, a mixture of **10a,b** reacted with three equivalents of DBU in DMF at 80 °C overnight. The reaction led to **28** in 23%. When changing the solvent from DMF to toluene the reaction, proceeded slowly. Thus, using 1.5 equivalents of DBU at 80 °C for 48 h with unchanged starting material, **10a,b** was received, together with a small amount of **28** (6%).

**Scheme 3:**
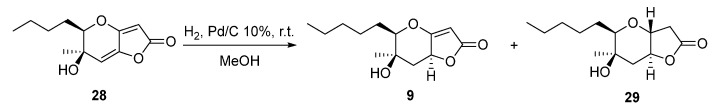
Catalytic hydrogenation reaction of compound **28**.

Another interesting phaeosphaeride analogue was obtained by reacting compound **28** under reductive catalytic hydrogenation conditions (Scheme 3). Specifically, the reaction was carried out in the presence of 10% Pd/C catalyst in MeOH solvent at room temperature for 30 min under an H_2_ atmosphere. The ^1^H NMR spectrum data indicated that the reduction took place at the double bond of the six membered ring forming compound **9** in 79% yield. The fully hydrogenated product **29** was also isolated in 18% yield.

**Figure 5:**
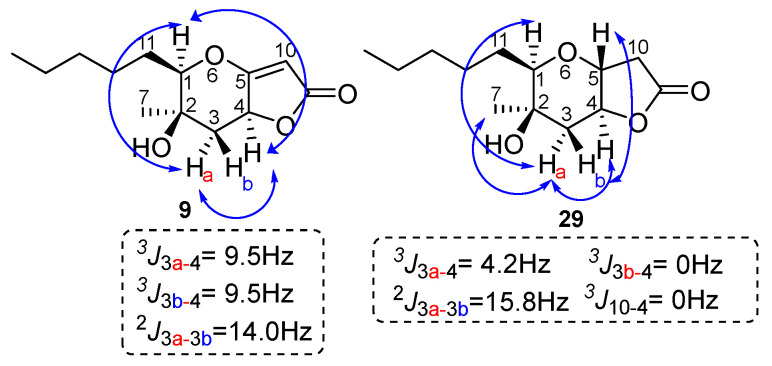
Relative stereochemistry for the furo[3,2-*b*]pyran-2-one system of **9** and **29**. Key NOE interactions are indicated by arrows.

**Figure 6:**
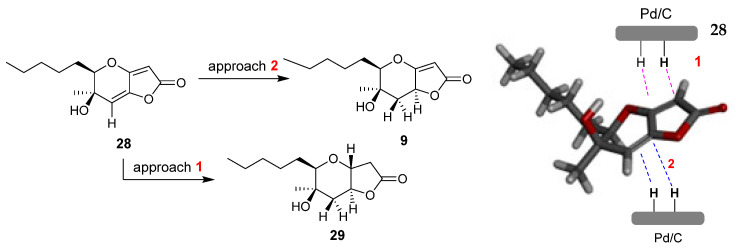
Catalytic hydrogenation of **28**. The modelling of compound **28** was performed with the MM2 method molecular energy calculations using Chem3D 15.0 software. BIOVIA Discovery Studio was used for the visualization of the 3D model.

To assign the stereochemistry of derivative **9**, the two-dimensional NOESY spectrum was recorded. As shown in Figure 5, there is a correlation between the C-4 hydrogen and the hydrogen of C-1 hydrogen. Thus, we conclude that the stereochemistry of compound **9** is that shown in Figure 5 below. This stereochemistry is considered more likely because the catalyst approaches **28** from side 2, due to the smaller hindrance of the methyl group compared to the hydroxy group on the opposite side (Figure 6). Compound **29** is produced when the catalyst approaches **28** from side 1, with the double bond of the five-membered unsaturated lactone first being reduced, or possibly being produced by the subsequent reduction of **9**. For compound **29**, the NOEs on H-5 with H-3b and H-3a with H-1, H-7 and H-4 defined the H-5, pentyl and OH group on the same side of the hexahydro-*2H*-furo[3,2-*b*]pyran-2-one, **28**.

**Scheme 4:**
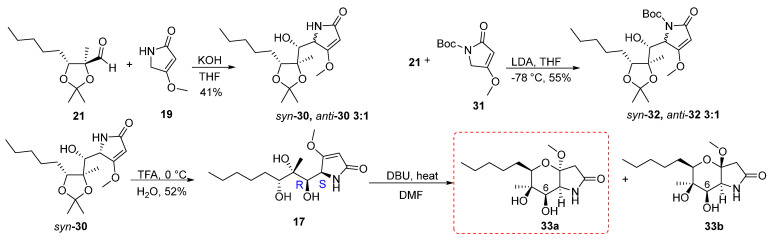
Synthesis of the pyrano[3,2-*b*]pyrrol-2(*1H*)-one **33a**.

The synthetic route followed to obtain the tetrahydropyrano[3,2-*b*]pyrrol-2(*1H*)-one **11** started from the vinylogous aldol reaction of aldehyde **21** with **19** (Scheme 4). First, the reaction was carried out using 1.6 equivalents of 4-methoxy-3-pyrrolin-2-one **19**, by adding an aqueous solution of KOH base (4M) in THF solvent at room temperature overnight. Formation of the anion of compound **19** and the addition to the aldehyde was carried out at 60 °C, after which the reaction was brought to room temperature. The reaction gave a mixture of adducts **30** in a ratio of *syn*:*anti* = 3:1 in a yield of 41%. The determination of the stereochemistry of the products was attempted at a later stage after the cyclization and formation of the pyran ring. The separation of the two isomers was troublesome due to the excess of **19**, as the products and starting material had similar Rf values when developed by thin layer chromatography. To optimize the reaction, one equivalent of **19** was used to facilitate the isolation process of the products. Furthermore, to control the formation of isomers, the addition of aldehyde **21** to the anion of compound **19** was carried out at 0 °C, while the reaction was then allowed to reach room temperature. But even in this case, two isomers were formed in a ratio of 4:1 and 13% yield, without the starting materials being completely consumed. When **21** reacted with the Boc protected pyrrolin-2-one **31** at −78 °C with LDA as a base in THF the vinylogous aldol reaction yielded adducts **32** in 55% yield in *syn*:*anti* = 3:1 ratio.

**Figure 7:**
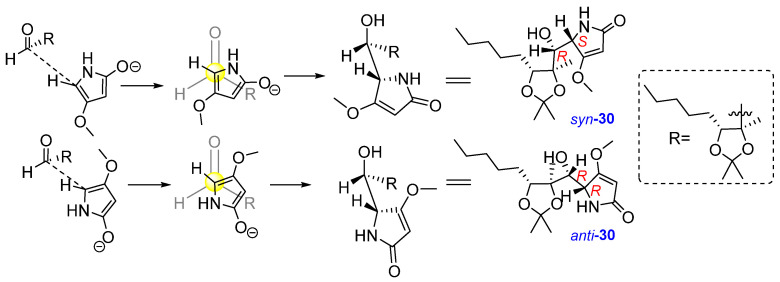
Formation of adducts *syn*-**30** and *anti*-**30**.

The addition of the 4-methoxy-1,5-dihydro-2*H*-pyrrol-2-one **19** to aldehyde **21** could result from the following vinylogous aldol reaction, shown in Figure 7. *Syn-anti* isomerization on the asymmetric centre of dihydro-2-pyrrolidone could also occur.

Having obtained the mixture of the two isomers **30** from the vinylogous aldol reaction, we proceeded to the next step of deprotection. During this reaction, *syn*-**30** was deprotected using TFA to give **17** in 50% yield. Then, **17** reacted with 1.5 equiv. of TBAF in THF solvent at 60 °C for 1 h; however, a complex mixture of compounds was received. In a second attempt, the reaction was carried out under the same conditions and left stirring overnight (Table 4). From the ^1^H spectrum a complex mixture of compounds was observed. In addition, the DBU base was used to achieve cyclization. The reaction was initially carried out using 1.5 equivalents of DBU in toluene solvent at room temperature overnight. However, we did not observe any change in the starting material. Thus, the reaction was carried out again under the same conditions but with heating at 60 °C, which led to the formation of two isomers of forms **33a,b**, in a 62% yield and in a 10:1 ratio. We were able to isolate and characterize only **33a**.

**Figure 8:**
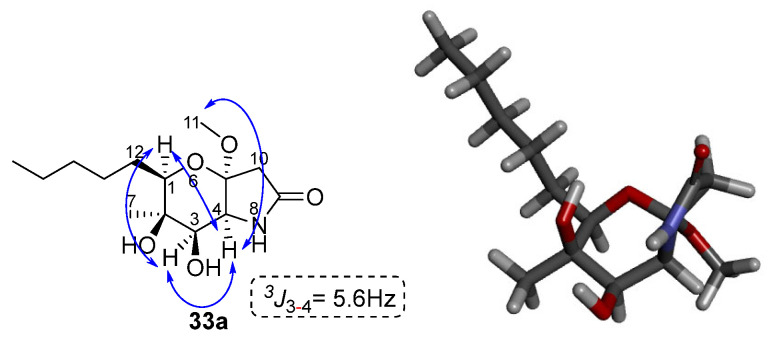
Key NOESY and correlations of compound **33a**. The modelling of compound **33a** was performed with the MM2 method molecular energy calculations using Chem3D 15.0 software. BIOVIA Discovery Studio was used for the visualization of the 3D model.

The stereochemistry of compound **33a** was determined based on the two-dimensional NOESY spectrum data. Specifically, as shown in Figure 8, correlation of the hydrogens of methyl 7 and N-H with the hydrogen of C-3 is observed. In addition, there is a correlation between the C-1 hydrogen with the C-3 and C-4 hydrogens. Finally, the C-4 hydrogen is correlated with the 11 methoxy group. Based on these observations, the stereochemistry of the cyclized product **33a** can be assigned as shown in Figure 8 below.

### 2.2. Cell Viability Studies

In the final phase of our study, we aimed to determine the anticancer potential of newly synthesized phaeosphaeride derivatives. We utilized two distinct cancer cell lines representing different malignancies: HeLa cells derived from cervical cancer and MOLT-4 cells from acute lymphoblastic leukemia. The cytotoxic activity of selected compounds was evaluated using the MTT metabolic assay. Cells were exposed to various concentrations of the compounds (ranging from 5 to 100 μM) for a duration of 48 h. The results, expressed as EC_50_ values (the concentration causing 50% loss of cell viability compared to non-treated cells), are presented in Table 5.

**Figure 9:**
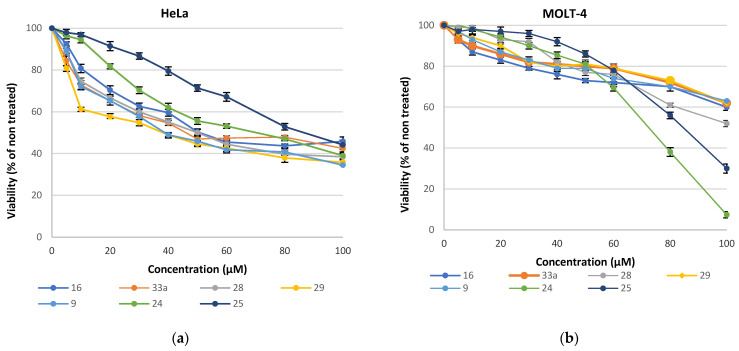
Growth inhibition of (**a**) HeLa cells; (**b**) Molt-4 cells grown in the presence of different concentrations of the synthesized compounds for 48 h. Values are means of three independent experiments, with the standard errors of the mean represented by vertical bars.

Analysis of the cytotoxic effects revealed that MOLT-4 cells (Figure 9b) exhibited greater resistance (EC_50_ > 70 μM) compared to HeLa cells (Figure 9a), except for **25**, which had a similar limited effect on both cell lines. Treatment of HeLa cells with the compounds showed a notably higher impact with the most potent compounds exhibiting EC_50_ values in the 40 μM range.

## 3. Discussion

Inspired by the structures of natural phaeosphaerides and paraphaesphaerides a series of analogues bearing the tetrahydro- and hexahydro-2*H*-furo[3,2-*b*]pyran-2-one and hexahydropyrano[3,2-*b*]pyrrol-2(1*H*)-one moieties were synthesized. Key synthetic steps included the vinylogous aldol reactions to aldehyde **21**, deprotection of the acetonide protecting group and intramolecular *oxy*-Michael reactions for the formation of the 3,4-dihydro-*2H*-pyran ring. The synthetic approach proved challenging in terms of diasteroselectivity and stability of synthetic intermediates; however, it has produced divergent sp^3^-enriched structures. Despite successful synthesis and structural modification, the majority of the phaeosphaeride analogues exhibited limited bioactivity against 2 cancer cells, HeLa and MOLT-4. The structure–activity relationship analysis suggested that the new modifications on the phaeosphaeride bicyclic system did not enhance anticancer potency and the *α,β*-unsaturated carbonyl functionality is important for activity. Phaeosphaerides appear to act through mixed mechanisms of action involving inhibition of the IL-6-activated STAT3 signalling pathway [18], oxidative stress and the modulation of JNK, ERK1/2, and p38 signalling pathways, according to Hirayama and Abzianidze [20,21]. As recently reported by Hirayama et al. phaeosphaeride A potently inhibits the proliferation of HeLa cells with IC_50_ value of 8.8 μΜ [18]. These studies indicate that the presence of exomethylene group at the C-3 position of phaeosphaeride is important for activity, suggesting that phaeosphaerides act as Michael acceptors in cancer cells. Our findings further support the importance of exomethylene group for activity, but also its high reactivity with nucleophiles such as water (demonstrated by the formation of **24**). In addition, the hydroxy groups and the alkyl moiety in cyclic or non-cyclic phaeosphaeride analogues contribute to the activity, as shown by the antiproliferative activity of **24** and **25**. The presence of double bonds and oxygen or nitrogen heteroatoms in furopyranones or pyranopyrrolones **9**, **28**, **29** and **33a** does not significantly impact cytotoxic activity. In HeLa cells, the synthesized compounds demonstrate moderate activity (EC_50_ values in the 40 μM range), and weaker activity against MOLT-4 cells. The most active compounds against MOLT-4 are the *α,β*-unsaturated carbonyl compounds 2-furanone **24** and lactone **25**.

## 4. Materials and Methods

General Experimental Details

Reactions were carried out under an atmosphere of Argon unless otherwise specified. Commercial starting materials were purchased from Merck (Darmstadt, Germany) or Alfa Aesar (Ward Hill, MA, USA) and used without further purification. Reactions were monitored by TLC using silica plates 60-F264 and UV light as a visualizing agent or aqueous ceric sulphate/phosphomolybdic acid, ethanolic *p*-anisaldehyde solution, potassium permanganate solution and heat as developing agents. ^1^H and ^13^C NMR spectra were recorded at 500 and 126 MHz (Agilent) with tetramethylsilane as an internal standard. Chemical shifts are given in δ values (ppm) from internal reference peaks (TMS ^1^H 0.00; CDCl_3_ ^1^H 7.26, ^13^C 77.16, (CD_3_)_2_SO ^1^H 2.50, ^13^C 39.52, (CD_3_)_2_CO ^1^H 2.05, ^13^C 29.84, 206.26). LC-MS analysis was performed on a LC-20AD Shimadzu connected to Shimadzu LCMS-2010EV (Shimadzu Kyoto, Japan) equipped with C_18_ analytical column (Supelco discovery C_18_, 5 μm 250 × 4.6 mm). HRMS experiments were carried out on a QExactive Plus Mass Spectrometer (Agilent, Santa Clara, CA, USA); flow rate 0.5 mL / min, 90% CAN + 0.1% HCOOH; spray voltage = 3 kV; capillary temperature = 300 °C. Melting points (mp) are uncorrected. Optical rotation was measured on a Kruss P-3000 polarimeter with a sodium lamp at the solvent, temperature, and concentration indicated for each compound. ESI-MS analysis and NMR spectra of all synthesized compounds are reported in the Supplementary Materials.

**4-methoxy-5-methylene-3-((1*S*,2*R*,3*S*)-1,2,3-trihydroxy-2-methyloctyl)furan-2(5*H*)-one, 13**

To a round bottom flask containing **23** [8] (19.3 mg, 0.057 mmol), a solution of TFA, in water (0.5 mL, 6.521 mmol, 1:1 *v*/*v*), was added. The reaction was stirred for 2 h at 0 °C, quenched with saturated aqueous NaHCO_3_ and extracted with DCM. The combined organic layers were washed with brine, dried over Na_2_SO_4,_ and concentrated under reduced pressure. The residue was purified by column chromatography (gradient elution PS–EA 2:1 to 1:2) to afford **13** (74%) as orange oil. **13**: ^1^H NMR (500 MHz, CDCl_3_) δ 5.16 (d, *J* = 3.0 Hz, 1H), 5.16 (d, *J* = 3.0 Hz, 1H), 4.95 (s, 1H), 4.21 (s, 4H), 3.63 (dd, *J* = 9.7, 2.7 Hz, 1H), 3,10 (br, 1H), 2.7 (br, 1H), 1.67–1.54 (m, 3H), 1.51–1.40 (m, 2H), 1.37–1.27 (m, 3H), 1.19 (s, 3H), 0.88 (t, *J =* 7.0 Hz, 3H); ^13^C NMR (126 MHz, CDCl_3_) δ 171.0, 163.7, 149.3, 105.3, 94.8, 77.1, 74.9, 69.8, 60.5, 31.8, 30.9, 25.9, 22.6, 18.9, 14.0; ESI-MS, positive mode: *m*/*z* calcd mass for C_15_H_24_O_6_ [M+Na]^+^ = 323.1471, was found to be 322.95.

**5-hydroxy-4-methoxy-5-methyl-3-((1*R*,2*S*,3*R*)-1,2,3-trihydroxy-2-methyloctyl) furan-2(5*H*)-one, 24**

To a solution of **13** (7.6 mg, 0.025 mmol) in 0.7 mL THF, TBAF (11 μL, 0.038 mmol, 1.6 M) was added. After stirring for 1 h at 60 °C, the reaction was quenched with saturated aqueous NaHCO_3_ and extracted with EA. The combined organic layers were washed with brine, dried over Na_2_SO_4_ and concentrated in vacuo. The residue was purified with column chromatography (gradient elution PS–EA 2:1 to 1:2) to afford the isomeric mixture **24** (20%) as orange oil. **24** (major isomer): ^1^H NMR (500 MHz, CDCl_3_) δ 4.62 (s, 1H), 4.31 (s, 3H), 4.07 (dd, *J* = 10.0, 2.3 Hz, 1H), 1.77–1.64 (m, 5H), 1.59 (s, 3H), 1.38 (m, 3H), 1.35 (s, 3H), 0.90 (t, *J* = 6.9 Hz, 3H); ^13^C NMR (126 MHz, CDCl_3_) δ 163.1, 161.0, 105.7, 94.5, 84.1, 83.7, 70.5, 62.4, 31.6, 28.7, 25.6, 22.8, 22.5, 21.3, 14.0; ESI-LCMS, positive mode: *m*/*z* calcd mass for C_15_H_24_NaO_7_ [M+Na]^+^ = 341.1576, was found to be 341.20.

**(4*R*,5*R*,6*R*,*E*)-4,5-dihydroxy-3-(1-methoxy-2-oxopropylidene)-5-methyl-6-pentyltetrahydro-2*H*-pyran-2-one**, **25**

To a solution of **13** (5.3 mg, 0.018 mmol) in 0.47 mL toluene, DBU (4 μL, 0.026 mmol) was added, and the reaction was stirred overnight at room temperature. The reaction mixture was then concentrated under reduced pressure and the residue subjected to column chromatography (gradient elution PS–EA 2:1 to 1:2) to form **25** (57%) as orange oil. **25**: ^1^H NMR (500 MHz, CDCl_3_) δ 4.90 (s, 1H), 3.89 (s, 3H), 3.47 (d, *J* = 9.7 Hz, 1H), 2.51 (s, 3H), 2.07 (br, 1H), 1.64–1.45 (m, 4H), 1.52–1.43 (m, 2H), 1.36 (s, 3H), 1.34–1.27 (m, 2H), 0.89 (t, *J* = 6.7 Hz, 3H); ^13^C NMR (126 MHz, CDCl_3_) δ 198.3, 168.6, 164.8, 106.9, 90.4, 76.0, 70.1, 58.1, 31.7, 30.9, 30.7, 25.9, 22.5, 15.3, 14.0; ESI-MS, positive mode: *m*/*z* calcd mass for C_15_H_24_O_6_ [M+Na]^+^ = 323.1471; 323.05 was found. Optical activity: observed rotation α_D_ = +2.27 (*c* = 0.0044 g/mL, T = 26.4 °C, CHCl_3_).

**(*S*)-5-((*R*)-hydroxy((4*R,*5*R*)-2,2,4-trimethyl-5-pentyl-1,3-dioxolan-4-yl)methyl)-4-methoxyfuran-2(5*H*)-one, *syn*-27**

To a solution of diisopropylamine (0.23 mL, 1.624 mmol) in 1 mL THF under argon at −78 °C, a solution of *n*-BuLi in THF (1.06 mL, 1.6 M) was added. After 45 min, a solution of **18** (110 mg, 0.96 mmol) in 0.8 mL THF was added dropwise over a 5 min period, followed by the addition of **21** (185 mg, 1.624 mmol) in 1.23 mL THF. The reaction mixture was stirred for 3 h at −78 °C. Upon completion, the reaction was quenched with 0.16 mL MeOH followed by 2.5 mL of sat. NH_4_Cl_(aq)_ and extracted with EA. The combined organic layers were washed with brine, dried over Na_2_SO_4_, and concentrated under reduced pressure. The residue was subjected to column chromatography (gradient elution PS–EA 3:1 to 1:2) to yield *syn-, anti*-**27** (49%) as orange oil. *Syn*-**27**: ^1^H NMR (500 MHz, CDCl_3_) δ 5.13 (s, 1H), 5.08 (s, 1H), 4.00 (d, *J* = 9.5 Hz, 1H), 3.92 (s, 3H), 3.91 (d, *J* = 1.8 Hz, 1H), 3.79 (s, 1H), 1.64–1.46 (m, 6H), 1.44 (s, 3H), 1.34 (s, 3H), 1.31 (m, 2H), 1.18 (s, 3H), 0.89 (t, *J* = 6.2 Hz, 3H); ^13^C NMR (126 MHz, CDCl_3_) δ 180.6, 172.5, 107.4, 89.4, 82.6, 82.5, 77.5, 74.2, 59.6, 31.8, 30.4, 28.8, 26.9, 26.9, 22.6, 16.8, 14.0; ESI-MS, positive mode: *m*/*z* calcd mass for C_17_H_28_O_6_ [M+Na]^+^ = 351.1784; 350.95 was found. Optical activity: observed rotation α_D_^25^ = −16.0 (*c* = 0.0025 g/mL, CHCl_3_).

**(*S*)-4-methoxy-5-((1*R*,2*S*,3*R*)-1,2,3-trihydroxy-2-methyloctyl)furan-2(5*H*)-one, 16**

To a round bottom flask containing *syn*-**27** (436.6 mg, 1.33 mmol) a solution of TFA in water (5.85 mL, 77.16 mmol, 1:1 *v*/*v*) was added. The reaction was stirred for 2 h at 0 °C, quenched with saturated aqueous NaHCO_3_ and extracted with DCM. The combined organic layers were washed with brine, dried over Na_2_SO_4,_ and concentrated in vacuo. The residue was purified with column chromatography (gradient elution PS–EA 1:3 to 1:6) to afford **16** (53%) as white solid. **16**: ^1^H NMR (500 MHz, DMSO-*d*_6_) δ 5.32 (s, 1H), 5.11 (s, 1H), 4.81 (d, *J* = 6.9 Hz, 1H), 4.33 (s, 1H), 4.18 (d, *J* = 7.6 Hz, 1H), 3.92 (d, *J* = 6.9 Hz, 1H), 3.86 (s, 3H), 3.81 (br, 1H), 1.50 (m, 2H), 1.25 (m, 6H), 1.03 (s, 3H), 0.85 (t, *J* = 6.1 Hz, 3H); ^13^C NMR (126 MHz, DMSO-*d*_6_) δ 182.4, 173.2, 89.9, 78.1, 74.8, 74.4, 70.7, 60.0, 32.0, 30.5, 26.3, 22.7, 19.5, 14.5. ESI-HRMS, positive mode: *m*/*z* calcd mass for C_14_H_24_O_6_ [M+Na]^+^ = 311.1471; 311.1465 was found. Optical activity: observed rotation α_D_^20^ = +26.1 (*c* = 0.0023 g/mL, MeOH). Melting range: 175–178 °C.

**(3*aS*,5*R*,6*R*,7*R*,7*aS*)-6,7-dihydroxy-3*a*-methoxy-6-methyl-5-pentylhexahydro-2*H*-furo[3,2-*b*]pyran-2-one, 10a and (3a*R*,5*R*,6*R*,7*R*,7a*S*)-6,7-dihydroxy-3*a*-methoxy-6-methyl-5-pentylhexahydro-2*H*-furo[3,2-*b*]pyran-2-one, 10b**

To a solution of **16** (113.7 mg, 0.396 mmol) in 10.5 mL DMF, DBU (0.09 mL, 0.592 mmol) was added, and the rection was stirred overnight at 60 °C. The reaction mixture was then concentrated under reduced pressure and the residue was subjected to column chromatography (gradient elution PS–EA 7:1 to 1:2) to yield **28** (12%) followed by **10** (74%, **10a**/**10b** = 4/1). **10a**: ^1^H NMR (500 MHz, CDCl_3_) δ 4.72 (d, *J* = 2.1 Hz, 1H), 4.22 (d, *J* = 2.1 Hz, 1H), 3.44 (d, *J* = 8.7 Hz, 2H), 3.38 (s, 3H), 2.87 (d, *J* = 17.6 Hz, 1H), 2.78 (d, *J* = 17.6 Hz, 1H), 1.44 (m, 2H), 1.32 (m, 6H), 1.25 (s, 3H), 0.89 (t, *J* = 6.9 Hz, 3H); ^13^C NMR (126 MHz, CDCl_3_) δ 173.3, 109.6, 93.5, 90.5, 77.6, 75.9, 51.3, 39.4, 31.7, 30.7, 25.97, 22.6, 16.4, 14.0. **10b**: ^1^H NMR (500 MHz, CDCl_3_) δ 4.75 (d, *J* = 2.9 Hz, 1H), 4.37 (d, *J* = 2.9 Hz, 1H), 3.50 (d, *J* = 9.6 Hz, 1H), 3.35 (s, 3H), 2.83 (d, *J* = 17.9 Hz, 1H), 2.78 (d, *J* = 17.9 Hz, 1H), 1.56 (m, 5H), 1.32 (s, 3H), 1.31 (s, 3H), 0.90 (t, *J* = 6.4 Hz, 3H); ^13^C NMR (126 MHz, CDCl_3_) δ 172.7, 109.6, 93.5, 89.4, 78.8, 76.6, 51.2, 39.4, 31.7, 31.0, 26.0, 22.6, 16.3, 14.0; ESI-MS, positive mode: *m*/*z* calcd mass for C_14_H_24_NaO_6_ [M+Na]^+^ = 311.1471, was found 310.90. **28**: ^1^H NMR (500 MHz, acetone-*d*_6_) δ 5.79 (d, *J* = 1.6 Hz, 1H), 5.24 (d, *J* = 1.6 Hz, 1H), 4.45 (br, 1H), 4.17 (dd, *J* = 9.9, 2.6 Hz, 1H), 1.95–1.80 (m, 3H), 1.74–1.65 (m, 1H), 1.52–1.44 (m, 1H), 1.41 (s, 3H), 1.39–1.31 (m, 3H), 0.92 (t, *J* = 6.9 Hz, 3H); ^13^C NMR (126 MHz, acetone-*d*_6_) δ 168.1, 166.7, 144.1, 110.1, 88.1, 87.6, 67.2, 31.5, 28.0, 25.6, 25.2, 22.3, 13.4; ESI-MS, negative mode: *m*/*z* calcd mass for C_13_H_18_O_4_ [M-H]^−^ = 237.1127; 236.90 was found. Optical activity: observed rotation α_D_^20^ = +64.15° (c = 0.0053 g/mL, MeOH).

**(5*R*,6*R*)-6-hydroxy-6-methyl-5-pentyl-5,6-dihydro-2*H*-furo[3,2-** ***b*** **]pyran-2-one, 28**

To a solution of mixture **10a,b** (42.2 mg, 0.146 mmol) in 3.8 mL DMF, DBU (0.07 mL, 0.430 mmol) was added, and the reaction was stirred overnight at 80 °C. The reaction mixture was then concentrated in vacuo, and its residue subjected to column chromatography (gradient elution PS–EA 7:1 to 4:1) to afford **28** in a 23% yield.

**(5*R,*6*R*,7a*S*)-6-hydroxy-6-methyl-5-pentyl-5,6,7,7a-tetrahydro-2*H*-furo[3,2-*b*]pyran-2-one**, **9 and (3*aR*,*5R*,*6R*,*7aS*)-6-hydroxy-6-methyl-5-pentylhexahydro-2*H*-furo[3,2-*b*]pyran-2-one**, **29**

To a solution of **28** (12.1 mg, 0.051 mmol) in 1.5 mL MeOH, a catalytic amount of Pd/C (10% *w*/*w*) was added under a hydrogen atmosphere. The reaction mixture was stirred for 30 min at room temperature, filtrated over celite and then concentrated under reduced pressure. The residue was subjected to column chromatography (gradient elution PS–EA 2:1 to 1:2) to yield **9** (79%) and **29** (18%) as orange oils. **9**: ^1^H NMR (500 MHz, CDCl_3_) δ 5.12 (d, *J* = 0.9 Hz, 1H), 4.93 (t, *J*= 9.5, 1H), 3.91 (d, *J* = 9.1 Hz, 1H), 2.62 (dd, *J* = 14.0, 9.5 Hz, 1H), 2.10 (br, 1H), 1.82 (dd, *J* = 14.0, 9.5 Hz, 1H), 1.74–1.63 (m, 2H), 1.36 (m, 6H), 1.32 (s, 3H), 0.91 (t, *J* = 6.8 Hz, 3H); ^13^C NMR (126 MHz, CDCl_3_) δ 180.5, 173.5, 88.4, 84.9, 71.4, 69.7, 42.4, 31.6, 27.6, 26.8, 25.4, 22.5, 14.0; ESI-MS, positive mode: *m*/*z* calcd mass for C_13_H_20_O_4_ [M+Na]^+^ = 263.1259; 263.15 was found. Optical activity: observed rotation α_D_ = +133.3 (c = 0.0033 g/mL, T = 22.8 °C, MeOH). **29**: ^1^H NMR (500 MHz, CDCl_3_) δ ^1^H NMR (500 MHz, CDCl_3_) δ 4.42 (d, *J* = 1.9 Hz, 1H), 4.23 (br, 1H), 3.16 (d, *J* = 8.1 Hz, 1H), 2.77–2.52 (m, 2H), 2.42 (d, *J* = 15.8 Hz, 1H), 2.34–2.13 (br, 1H), 1.79 (dd, *J* = 15.8, 4.2 Hz, 1H), 1.55 (m 2H), 1.27 (m, 6H), 1.12 (s, 3H), 0.89 (t, *J* = 6.8 Hz, 3H); ^13^C NMR (126 MHz, CDCl_3_) δ 175.2, 82.2, 77.7, 73.9, 67.5, 38.4, 38.3, 31.8, 28.0, 25.6, 24.6, 22.6, 14.0; ESI-MS, positive mode: *m*/*z* calcd mass for C_13_H_22_O_4_ [M+H]^+^ = 243.1596; 243.1572 was found.

**(*S*)-5-((*R*)-hydroxy((4*R*,5*R*)-2,2,4-trimethyl-5-pentyl-1,3-dioxolan-4-yl)methyl)-4-methoxy-1,5-dihydro-2*H*-pyrrol-2-one, *syn*-30, (*R*)-5-((*R*)-hydroxy((4*R*,5*R*)-2,2,4-trimethyl-5-pentyl-1,3-dioxolan-4-yl)methyl)-4-methoxy-1,5-dihydro-2*H*-pyrrol-2-one, *anti*-30**

To a solution of **19** (17 mg, 0.149 mmol) in 0.7 mL THF, KOH_(aq)_ (0.043 mL, 4M) was added, dropwise, and the reaction was stirred at 60 °C. After one hour, a solution of **21** (20 mg, 0.093 mmol) in 0.6 mL THF was added and the reaction mixture was stirred at room temperature overnight. Upon completion, the reaction was concentrated under reduced pressure and its residue was subjected to column chromatography (gradient elution DCM-MeOH 10:0.4 to 10:0.7) to afford **30** (41%, *syn*-**30**/*anti*-**30** = 3/1) as orange oil. *Syn***-30**: ^1^H NMR (500 MHz, CDCl_3_) δ 5.69 (s, 1H), 5.07 (s, 1H), 4.39 (s, 1H), 3.84 (1H, overlapping with OMe), 3.83 (s, 3H), 3.76 (d, *J* = 9.9 Hz, 1H), 2.73 (d, *J* = 10.4 Hz, 1H), 1.60–1.47 (m, 3H), 1.42 (s, 3H), 1.35 (s, 3H), 1.31 (m, 5H), 1.16 (s, 3H), 0.89 (t, *J* = 6.9 Hz, 3H); ^13^C NMR (126 MHz, CDCl_3_) δ 176.7, 175.4, 107.7, 94.7, 84.3, 81.7, 72.5, 58.5, 31.8, 29.7, 28.6, 26.9, 26.8, 22.5, 17.8, 14.0; ESI-MS, negative mode: *m*/*z* calcd mass for C_17_H_28_NO_5_ [M-H]^-^ = 326.1967; 326.05 was found. *Anti*-**30**: ^1^H NMR (500 MHz, CDCl_3_) δ 7.78 (s, 1H), 5.16 (s, 1H), 5.09 (s, 1H), 3.85 (1H, overlapping with OMe), 3.83 (s, 3H), 1.57 (m, 4H), 1.46 (s, 3H), 1.20 (s, 3H), 1.33 (m, 4H), 0.89 (t, *J* = 6.9 Hz, 3H); ^13^C NMR (126 MHz, CDCl_3_) δ 170.7, 166.2, 132.4, 108.5, 108.1, 93.0, 83.2, 82.5, 58.2, 32.0, 29.1, 28.6, 26.7, 26.4, 22.7, 22.3, 14.2; ESI-MS, negative mode: *m*/*z* calcd mass for C_17_H_29_O_5_ [M-H]^−^ = 326.1973; 326.1971 was found.

***tert*** **-butyl (*S*)-2-((*R*)-hydroxy((4*R*,5*R*)-2,2,4-trimethyl-5-pentyl-1,3-dioxolan-4-yl)methyl)-3-methoxy-5-oxo-2,5-dihydro-1*H*-pyrrole-1-carboxylate, *syn*-32, *tert*-butyl (*R*)-2-((*R*)-hydroxy((4*R,*5*R*)-2,2,4-trimethyl-5-pentyl-1,3-dioxolan-4-yl)methyl)-3-methoxy-5-oxo-2,5-dihydro-1*H*-pyrrole-1-carboxylate, *anti*-32**

To a solution of diisopropylamine (0.23 mL, 1.66 mmol) in 1 mL THF under argon at 0 °C, a solution of *n*-BuLi in THF (1.08 mL, 1.6 M) was added, and the overall mixture was transferred to a dry ice bath (−78 °C) to stir. After 20 min, a solution of **31** (345.9 mg, 1.66 mmol) in 1.47 mL THF was added dropwise over 5 min, followed by the addition of **21** (118.59 mg, 0.55 mmol) in 0.57 mL THF. The reaction mixture was stirred for 2 h at −78 °C. Upon completion, the reaction was quenched with 0.16 mL MeOH followed by sat. NH_4_Cl_(aq)_ and extracted with EA. The combined organic layers were washed with brine, dried over Na_2_SO_4_, and concentrated under reduced pressure. The residue was subjected to column chromatography (gradient elution PS–EA 3:1 to 2:1) to yield the isomeric mixture **32** (55%) as orange oil. *Syn*-**32**: ^1^H NMR (500 MHz, CDCl_3_) δ 5.26 (d, *J* = 6.1Hz, 1H), 5.01 (s, 1H), 4.72 (s, 1H), 4.07 (d, *J* = 6.3Hz, 1H), 3.83 (overlapping with OMe, 1H), 3.80 (s, 3H), 1.68 (m, 1H), 1.54 (s, 9H), 1.53-1.33 (m, 3H), 1.22-1.30 (m, 4H), 1.08 (s, 3H), 0.88 (t, *J* = 6.8 Hz, 3H); ^13^C NMR (126 MHz, CDCl_3_) δ 176.0, 168.4, 153.7, 107.1, 94.2, 85.6, 84.4, 83.4, 65.0, 58.5, 32.2, 30.2, 28.9, 28.3, 27.5, 26.5, 22.8, 16.2, 14.2; ESI-MS, positive mode: *m*/*z* calcd mass for C_22_H_37_O_7_ [M+Na]^+^ = 450.2468; 450.2448 was found. *Anti*-**32**: ^1^H NMR (500 MHz, CDCl_3_) δ 5.06 (s, 1H), 4.80 (s, 1H), 4.20 (m, 2H), 3.84 (overlapping with OMe, 1H), 3.84 (s, 3H), 1.56 (s, 9H), 1.25-1.55 (m, 8H), 1.25 (s, 3H), 0.89 (t, *J* = 6.5 Hz, 3H); ^13^C NMR (126 MHz, CDCl_3_) δ 175.1, 168.3, 107.6, 95.2, 83.9, 83.4, 79.5, 70.5, 58.7, 32.2, 29.1, 28.3, 28,1, 26.9, 26.4, 22.7, 18.0, 14.2; ESI-MS, positive mode: *m*/*z* calcd mass for C_22_H_37_O_7_ [M+Na]^+^ = 450.2468; 450.2445 was found.

**(*S*)-4-methoxy-5-((*1R,2S,3R*)-1,2,3-trihydroxy-2-methyloctyl)-1,5-dihydro-2*H*-pyrrol-2-one, 17**

To a round bottom flask containing *syn*-**30** (8 mg, 0.024 mmol), a solution of TFA in water (0.22 mL, 2.81 mmol, 1:1 *v*/*v*) was added. The reaction was stirred for 30 min at 0 °C quenched with saturated aqueous NaHCO_3_ and extracted with DCM. The combined organic layers were washed with brine, dried over Na_2_SO_4,_ and concentrated in vacuo. The residue was purified with column chromatography (gradient elution DCM–MeOH 10:0.2 to 10:0.7) to afford **17** (50%). **17**: ^1^H NMR (500 MHz, CDCl_3_) δ 6.90 (s, 1H), 5.03 (s, 1H), 4.48 (br, 1H), 4.34 (br, 1H), 4.23 (s, 1H), 3.82 (overlapping with OMe, 1H), 3.80 (s, 3H), 3.60 (d, *J* = 9.40 Hz, 1H), 1.25-1.41 (m, 8H), 1.26 (s, 3H), 0.89 (t, *J* = 6.75Hz, 3H); ^13^C NMR (126 MHz, CDCl_3_) δ 171.2, 166.5, 133.8, 110.7, 93.0, 78.0, 58.5, 32.2, 32.0, 30.1, 26.7, 23.5, 23.0, 14.4; ESI-MS, positive mode: *m*/*z* calcd mass for C_14_H_25_O_5_ [M+Na]^+^ = 310.1630; 310.1618 was found.

**(3*aR,*5*R,*6*R,*7*R,*7*aS*)-6,7-dihydroxy-3a-methoxy-6-methyl-5-pentylhexahydropyrano[3,2-*b*]pyrrol-2(1*H*)-one**, **33a**

To a solution of **17** (6.8 mg, 0.024 mmol) in 0.63 mL toluene was added DBU (5 μL, 0.036 mmol) and the reaction was stirred overnight at 60 °C. The reaction mixture was then concentrated under reduced pressure and the residue subjected to column chromatography (gradient elution DCM–MeOH 10:0.2 to 10:0.5) to afford **33a** (62%) as yellow solid. **33a**: ^1^H NMR (500 MHz, CDCl_3_) δ 6.65 (s, 1H), 4.39 (d, *J* = 5.6 Hz, 1H), 4.10 (d, *J* = 5.6 Hz, 1H), 3.39 (t, *J* = 6.7 Hz, 1H), 3.36 (s, 3H), 2.70 (br, 2H), 2.44 (br, 1H), 1.74–1.49 (m, 2H), 1.33 (m, 6H), 1.19 (s, 3H), 0.90 (t, *J* = 6.7 Hz, 3H); ^13^C NMR (126 MHz, CDCl_3_) δ 174.1, 110.3, 91.6, 76.8, 73.7, 66.6, 51.3, 41.2, 31.9, 31.4, 26.2, 22.6, 17.3, 14.0; ESI-MS, positive mode: *m*/*z* calcd mass for C_14_H_26_O_5_ [M+H]^+^ = 288.1811; 287.90 was found.

Cell Toxicity

Cell Culture: HeLa and MOLT-4 cancer cell lines were kindly provided by Professor Eleni Nikolakaki (Laboratory of Biochemistry, Department of Chemistry of Aristotle University of Thessaloniki). HeLa cells were maintained at 37 °C with 5% CO_2_ in DMEM, while MOLT-4 cells were maintained in RPMI medium, both supplemented with 10% (*v*/*v*) fetal bovine serum (FBS) and antibiotics/antimycotics.

MTT Assays: Cells (HeLa and MOLT-4) were seeded in 96-well plates (3 × 10^3^ cells for HeLa and 15 × 10^3^ per well) and one day after were exposed to increasing concentrations of the inhibitors for 48 h. The viability of the cells was estimated by a (3-(4,5-dimethylthiazol-2-yl)-2,5-diphenyltetrazolium bromide (MTT) metabolic colorimetric assay as described previously [22]. The absorbance values (the means ± SE of three independent experiments) of treated cells were normalized to the untreated cells, which was set as 100% viability. Half maximal effective concentration where then calculated (EC_50_ values).

## 5. Conclusions

In summary, a synthetic route towards the synthesis of several synthetic analogues of phaeosphaerides employing vinylogous aldol and intramolecular Michael reactions was explored. Further optimization of the vinylogous reaction steps is necessary; however, it was possible to create sp^3^-rich scaffolds with multiple stereogenic centres related to the natural phaeosphaerides, worthy of more chemical and pharmacological exploration. These findings highlight the challenges in optimizing phaeosphaerides for anticancer applications and provide insights into future structural modifications to improve their therapeutic potential.

## Figures and Tables

**Table 1:** Conditions for the cyclisation of tetronate **13**.

Entry	Reagent	Equiv.	Temperature (°C)	Time (h)	Yield
**1**	TBAF (1.6M)	1.5	60	1	**24** (20%) + complex mixture
**2**	TBAF (1.6M)	1.5	rt	overnight	**24** (11%) + complex mixture
**3**	DBU	1.5	rt	overnight	**25** (57%)
**4**	*p*-TSA	1.5	60	0.5	**24** (10%) + complex mixture

**Table 2:** ^1^H and ^13^C NMR Data for **25**, at 500 and 125 MHz.

Position	δ_C_ (mult.)	δ_H_ (mult., *J* in Hz)	Position	δ_C_ (mult.)	δ_H_ (mult., *J* in Hz)
**1**	-	-	**9**	31.7	1.64–1.45 (m, 2H)
**2**	168.6	-	**10**	22.5	1.34–1.27 (m, 2H)
**3**	106.9	-	**11**	14.0	0.89 (t, *J* = 6.7 Hz, 3H)
**4**	70.1	4.90 (s, 1H)	**12**	15.3	1.36 (s, 3H)
**5**	90.4	-	**13**	198.3	-
**6**	76.0	3.47 (d, *J* = 9.7 Hz, 1H)	**14**	30.7	2.51 (s, 3H)
**7**	30.7	1.64–1.45 (m, 2H)	**15**	164.8	-
**8**	25.9	1.52–1.43 (m, 2H)	**16**	58.1	3.89 (s, 3H)

**Table 3:** Cyclisation of **16** in DMF.

Entry	Equiv.	Temperature (°C)	Time (h)	Yield
**1**	1.5	60	72	**10a,b** (69%), **28** (39%)
**2**	1.5	60	24	**10a,b** (74%), **28** (12%)
**3**	3	80	48	**10a,b** (46%), **28** (47%)

**Table 4:** Cyclisation of **33a** in DMF.

Entry	1.5 Equiv.	Temperature (°C)	Solvent	Time (h)	Yield
**1**	TBAF	60	THF	1	complex mixture
**2**	TBAF	60	THF	24	complex mixture
**3**	DBU	rt	Toluene	24	no reaction
**4**	DBU	60	Toluene	24	**33a,b** (86%)

**Table 5:** Half maximal effective concentration (EC_50_ Values, μΜ) of selected compounds against HeLa and MOLT-4 cancer cell lines. Results are presented as mean ± SE of three independent experiments.

Compound	HeLa (μΜ)	MOLT-4 (μΜ)	Compound	HeLa (μΜ)	MOLT-4 (μΜ)
**16**	50.3 ± 2.1	>100	**25**	88.6 ± 3.2	83.8 ± 2.9
**24**	57.9 ± 1.6	71.8 ± 2.4	**9**	43.5 ± 2.1	>100
**28**	49.4 ± 2.4	>100	**29**	39.1 ± 1.9	>100
**33a**	46.2 ± 3.7	>100			

## Data Availability

Data are contained within the article and Supplementary Materials.

## Supplementary Materials

The following supporting information can be downloaded at https://www.mdpi.com/article/10.3390/molecules30092016/s1: Figures S1–S51: 1H-NMR and 13C-NMR spectra for 13; ESI-LCMS analysis for 13; 1H-NMR and 13C-NMR spectra for 24; ESI-LCMS analysis for 24; 1H-NMR and 13C-NMR spectra for 25; 1H-1H COSY and 1H-1H NOESY spectra for 25; 1H-13C HSQC and 1H-13C HMBC spectra for 25; ESI-LCMS analysis of 25; 1H-NMR spectrum for syn-,anti-27; ESI-LCMS analysis for syn-,anti-27; 1H-NMR and 13C-NMR spectra for syn-27; 1H-NMR and 13C-NMR spectra for 16; 1H-1H COSY and 1H-13C HMBC spectra for 16; 1H-13C HSQC spectrum 16; ESI-LCMS analysis of 16; 1H-NMR spectrum for 10a; 13C-NMR and 1H-1H NOESY spectra for 10a; 1H-NMR and 13C-NMR spectra for 10b; 1H-1H NOESY spectrum for 10b; 1H-13C HSQC and 1H-13C HMBC spectra for 10b; ESI-LCMS analysis of 10b; 1H-NMR and 13C-NMR spectra for 28; 1H-1H COSY and 1H-13C HMBC spectra for 28; 1H-13C HSQC spectrum for 28; ESI-LCMS analysis of 28; 1H-NMR and 13C-NMR spectra for 9; 1H-1H COSY and 1H-13C HSQC spectra for 9; 1H-13C HMBC and 1H-1H NOESY spectra for 9; ESI-LCMS analysis of 9; 1H-NMR and 13C-NMR spectra for 29; 1H-1H COSY and 1H-1H NOESY spectra for 29; 1H-13C HSQC and 1H-13C HMBC spectra for 29; ESI-LCMS analysis of 29; 1H-NMR and 13C-NMR spectra for syn-30; 1H-1H COSY and 1H-13C HMBC spectra for syn-30; 1H-13C HSQC spectrum for syn-30; MS analysis for syn-30; 1H-NMR and 13C-NMR spectra for anti-30; 1H-1H COSY and 1H-1H NOESY spectra for anti-30; 1H-13C HMBC and 1H-13C HSQC spectra for anti-30; HRMS analysis for anti-30; 1H-NMR and 13C-NMR spectra for syn-32; 1H-NMR and 13C-NMR spectra for anti-32; HRMS analysis for anti-32; 1H-NMR and 13C-NMR spectra for 17; ESI-LCMS analysis of 17; 1H-NMR and 13C-NMR spectra for 33a; 1H-1H COSY spectrum and HMBC spectrum for 33a; 1H-13C HSQC and 1H-1H NOESY spectra for 33a; 1H-1H NOESY spectrum for 33a; ESI-LCMS analysis of 33a; HPLC-MS parameters and method development. Table S1: Conditions for the elution of 33a.

## Abbreviations

The following abbreviations are used in this manuscript:

STAT3	Signal Transducer and Activator of Transcription 3
SAR	Structure Activity Relationships
DMEM	Dulbecco’s Modified Eagle Medium
TBAF	Tetrabutylammonium fluoride
DBU	1,8-Diazabicyclo(5.4.0)undec-7-ene
TFA	Trifluoroacetic acid
*p*TSA	*p*-Toluenesulfonic acid
