# Supplementary material for: Synthesis and Biological Screening of Structurally Modified Phaeosphaeride Analogues
Source: Molecules. 2025 Apr 30;30(9):2016. doi: 10.3390/molecules30092016 (PMC12073321; doi:10.3390/molecules30092016)
Supplement: Supplementary file 1 [file molecules-30-02016-s001.zip › molecules-3579558-supplementary.pdf]

**Supplementary Material**

# Synthesis and Biological Screening of Structurally Modified Phaeosphaeride Analogues

Konstantinos Rantzios, Oraia-Eirini Chatzimentor, George Leonidis, Jorgo Giuliani, Ioanna Sigala and Vasiliki Sarli*

Department of Chemistry, Aristotle University of Thessaloniki, University Campus, 54124, Thessaloniki, Greece

*Corresponding Authors: sarli@chem.auth.gr

## Supporting Information

| | |
|---|---|
| $^{1}H$-NMR and $^{13}C$-NMR spectra for **13** | **S3** |
| ESI-LCMS analysis for **13** | **S4** |
| $^{1}H$-NMR and $^{13}C$-NMR spectra for **24** | **S5** |
| ESI-LCMS analysis for **24** | **S6** |
| $^{1}H$-NMR and $^{13}C$-NMR spectra for **25** | **S7** |
| $^{1}H$-$^{1}H$ COSY and $^{1}H$-$^{1}H$ NOESY spectra for **25** | **S8** |
| $^{1}H$-$^{13}C$ HSQC and $^{1}H$-$^{13}C$ HMBC spectra for **25** | **S9** |
| ESI-LCMS analysis of **25** | **S10** |
| $^{1}H$-NMR spectrum for *syn-,anti*-**27** | **S11** |
| ESI-LCMS analysis for *syn-,anti*-**27** | **S11** |
| $^{1}H$-NMR and $^{13}C$-NMR spectra for *syn*-**27** | **S12** |
| $^{1}H$-NMR and $^{13}C$-NMR spectra for **16** | **S13** |
| $^{1}H$-$^{1}H$ COSY and $^{1}H$-$^{13}C$ HMBC spectra for **16** | **S14** |
| $^{1}H$-$^{13}C$ HSQC spectrum **16** | **S15** |
| ESI-LCMS analysis of **16** | **S15** |
| $^{1}H$-NMR spectrum for **10a** | **S16** |
| $^{13}C$-NMR and $^{1}H$-$^{1}H$ NOESY spectra for **10a** | **S17** |
| $^{1}H$-NMR and $^{13}C$-NMR spectra for **10b** | **S18** |
| $^{1}H$-$^{1}H$ NOESY spectrum for **10b** | **S19** |
| $^{1}H$-$^{13}C$ HSQC and $^{1}H$-$^{13}C$ HMBC spectra for **10b** | **S20** |

| | |
|---|---|
| ESI-LCMS analysis of **10b** | **S21** |
| $^{1}H$-NMR and $^{13}C$-NMR spectra for **28** | **S22** |
| $^{1}H$-$^{1}H$ COSY and $^{1}H$-$^{13}C$ HMBC spectra for **28** | **S23** |
| $^{1}H$-$^{13}C$ HSQC spectrum for **28** | **S24** |
| ESI-LCMS analysis of **28** | **S25** |
| $^{1}H$-NMR and $^{13}C$-NMR spectra for **9** | **S26** |
| $^{1}H$-$^{1}H$ COSY and $^{1}H$-$^{13}C$ HSQC spectra for **9** | **S27** |
| $^{1}H$-$^{13}C$ HMBC and $^{1}H$-$^{1}H$ NOESY spectra for **9** | **S28** |
| ESI-LCMS analysis of **9** | **S29** |
| $^{1}H$-NMR and $^{13}C$-NMR spectra for **29** | **S30** |
| $^{1}H$-$^{1}H$ COSY and $^{1}H$-$^{1}H$ NOESY spectra for **29** | **S31** |
| $^{1}H$-$^{13}C$ HSQC and $^{1}H$-$^{13}C$ HMBC spectra for **29** | **S32** |
| ESI-LCMS analysis of **29** | **S33** |
| $^{1}H$-NMR and $^{13}C$-NMR spectra for *syn*-**30** | **S34** |
| $^{1}H$-$^{1}H$ COSY and $^{1}H$-$^{13}C$ HMBC spectra for *syn*-**30** | **S35** |
| $^{1}H$-$^{13}C$ HSQC spectrum for *syn*-**30** | **S36** |
| MS analysis for *syn*-**30** | **S36** |
| $^{1}H$-NMR and $^{13}C$-NMR spectra for *anti*-**30** | **S37** |
| $^{1}H$-$^{1}H$ COSY and $^{1}H$-$^{1}H$ NOESY spectra for *anti*-**30** | **S38** |
| $^{1}H$-$^{13}C$ HMBC and $^{1}H$-$^{13}C$ HSQC spectra for *anti*-**30** | **S39** |
| HRMS analysis for *anti*-**30** | **S40** |
| $^{1}H$-NMR and $^{13}C$-NMR spectra for *syn*-***32*** | **S41** |
| $^{1}H$-NMR and $^{13}C$-NMR spectra for *anti*-***32*** | **S42** |
| HRMS analysis for *anti*-***32*** | **S43** |
| $^{1}H$-NMR and $^{13}C$-NMR spectra for **17** | **S44** |
| ESI-LCMS analysis of **17** | **S45** |
| $^{1}H$-NMR and $^{13}C$-NMR spectra for **33a** | **S46** |
| $^{1}H$-$^{1}H$ COSY spectrum and HMBC spectrum for **33a** | **S47** |
| $^{1}H$-$^{13}C$ HSQC and $^{1}H$-$^{1}H$ NOESY spectra for **33a** | **S48** |
| $^{1}H$-$^{1}H$ NOESY spectrum for **33a** | **S49** |
| ESI-LCMS analysis of **33a** | **S50** |
| HPLC-MS parameters and method development | **S51** |

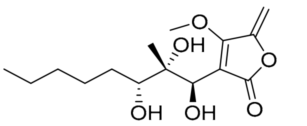


**13**

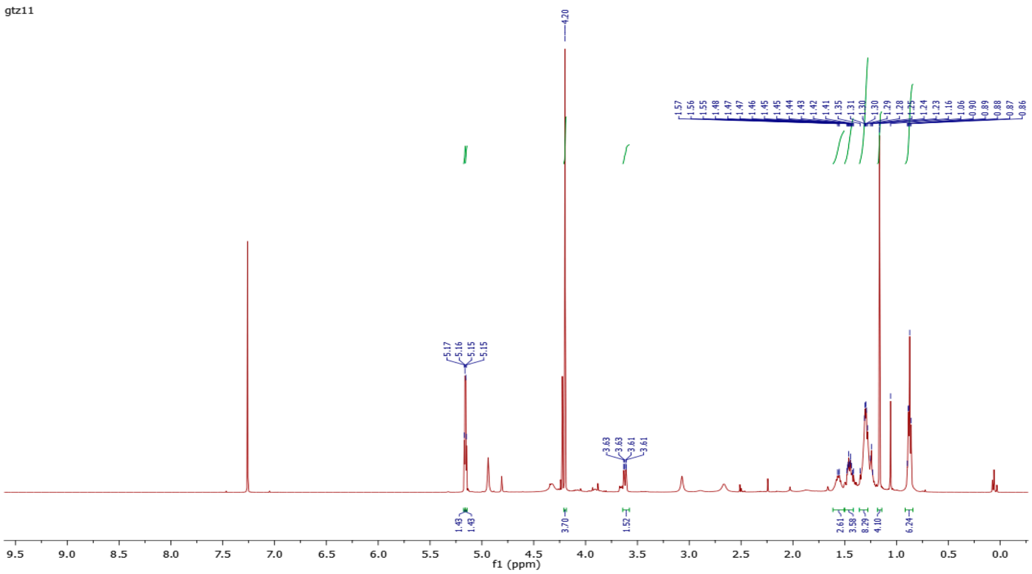


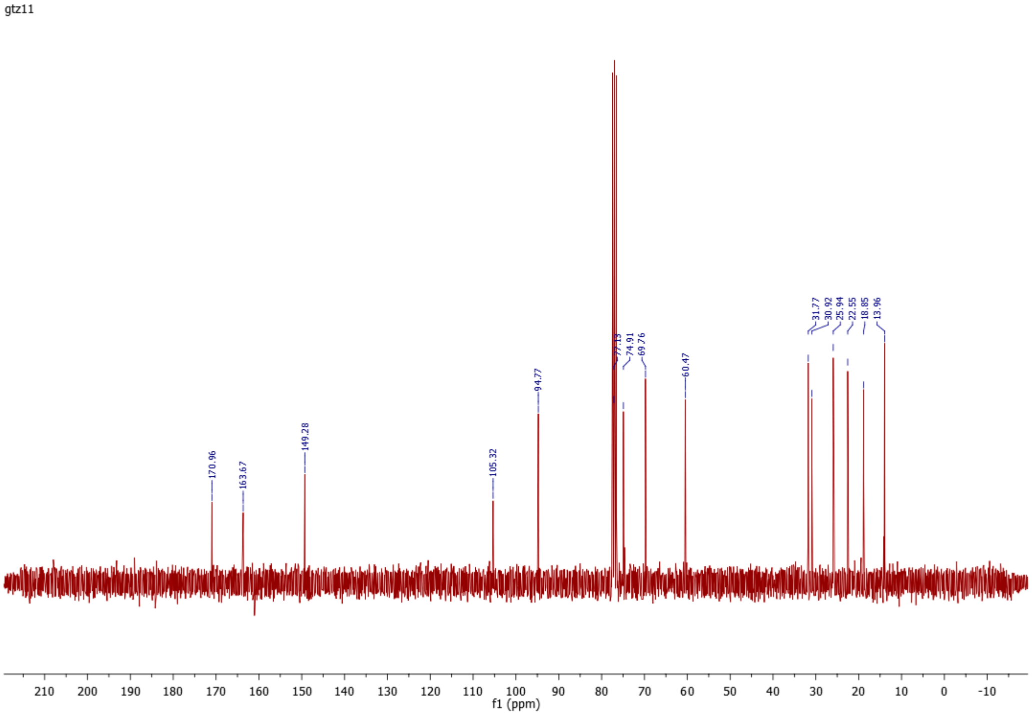


***Figure S1:*** $^{1}$H-NMR and $^{13}$C-NMR spectra for **13**

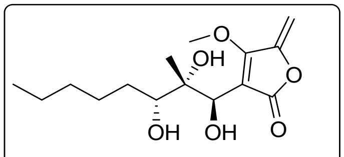


**13**

The compound **13** was eluted isocratically with methanol, with a retention time of 8.1 minutes. LC-MS analysis revealed a main peak with a relative area of 99.2 out of a total relative area of 100.0, corresponding to a calculated purity of 99.2%.

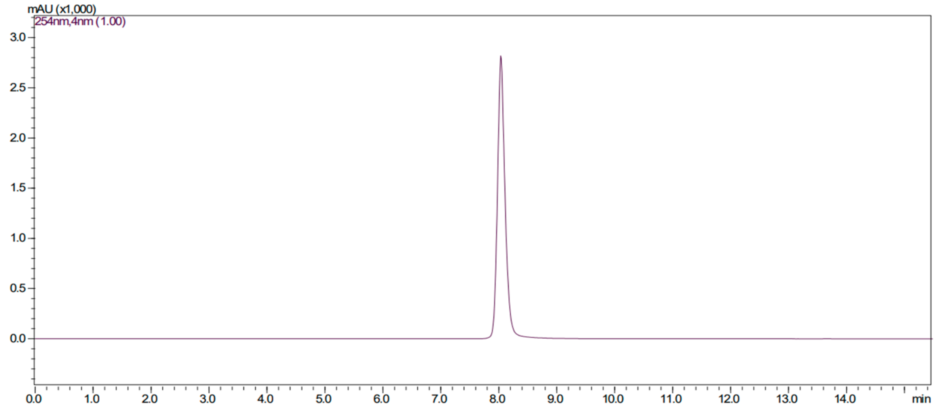


ESI-MS, positive mode: m/z calcd mass for $C_{15}H_{24}O_6$ $[M+Na]^+$ = 323.1471, was found 322.95.

(+)

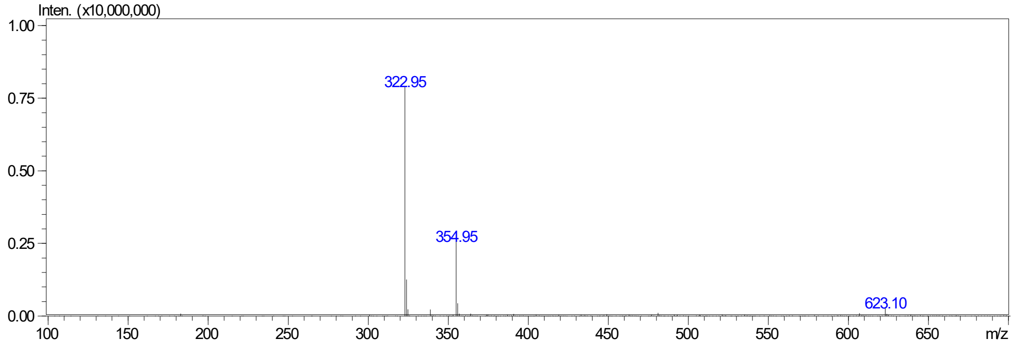


***Figure S2:*** ESI-LCMS analysis for **13**

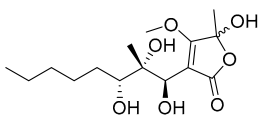


**24**

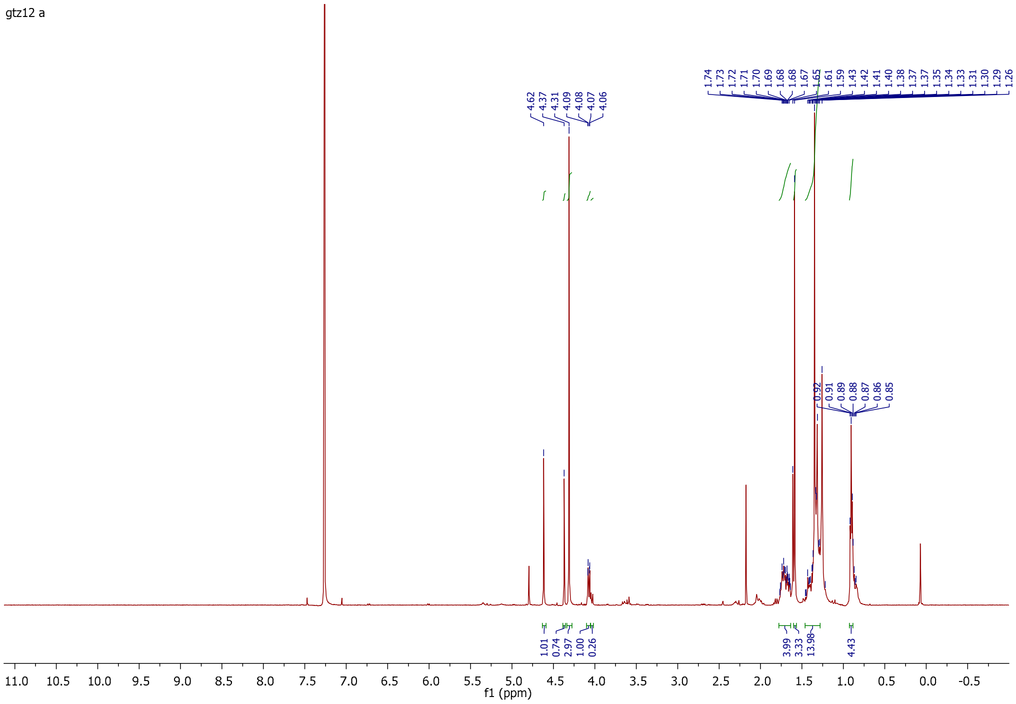


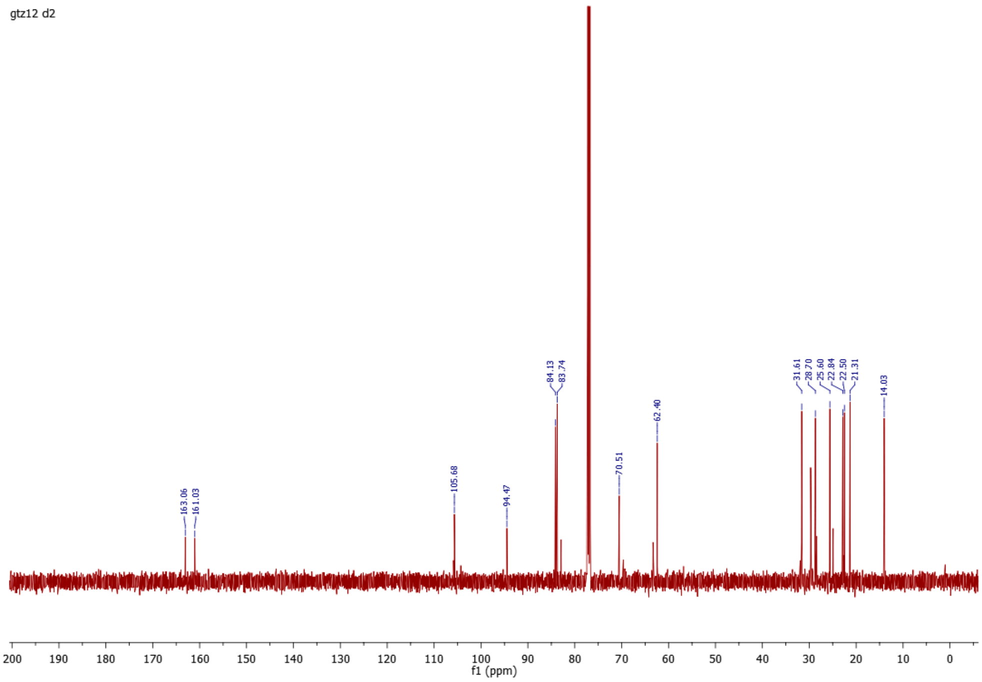


***Figure S3:*** $^{1}$H-NMR and $^{13}$C-NMR spectra for **24**

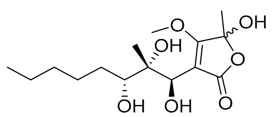


**24**

The compound **24** was eluted isocratically with methanol, with a retention time of 15.2 minutes. LC-MS analysis revealed a main peak with a relative area of 94.6 out of a total relative area of 100.0, corresponding to a calculated purity of 94.6%.

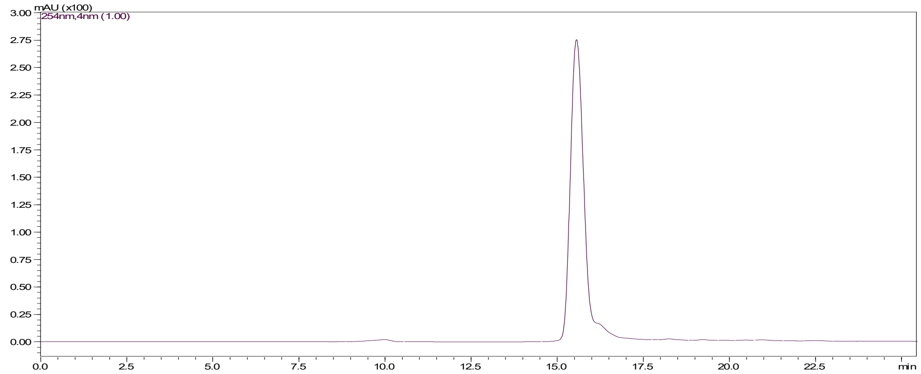


ESI-LCMS, positive mode: m/z calcd mass for $C_{15}H_{26}NaO_7$ $[M+Na]^+$ = 341.1576, was found 341.20.

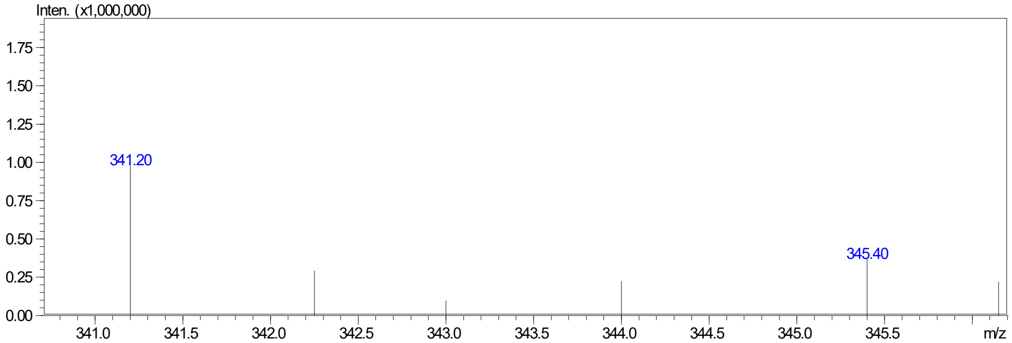


***Figure S4:*** ESI-LCMS analysis for **24**

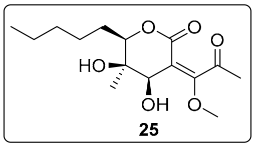

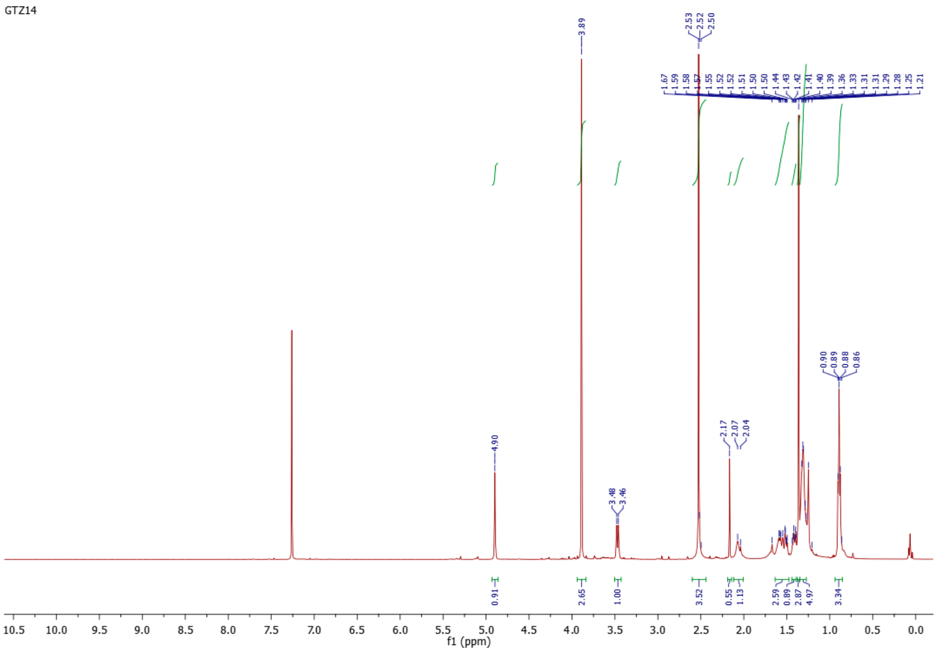

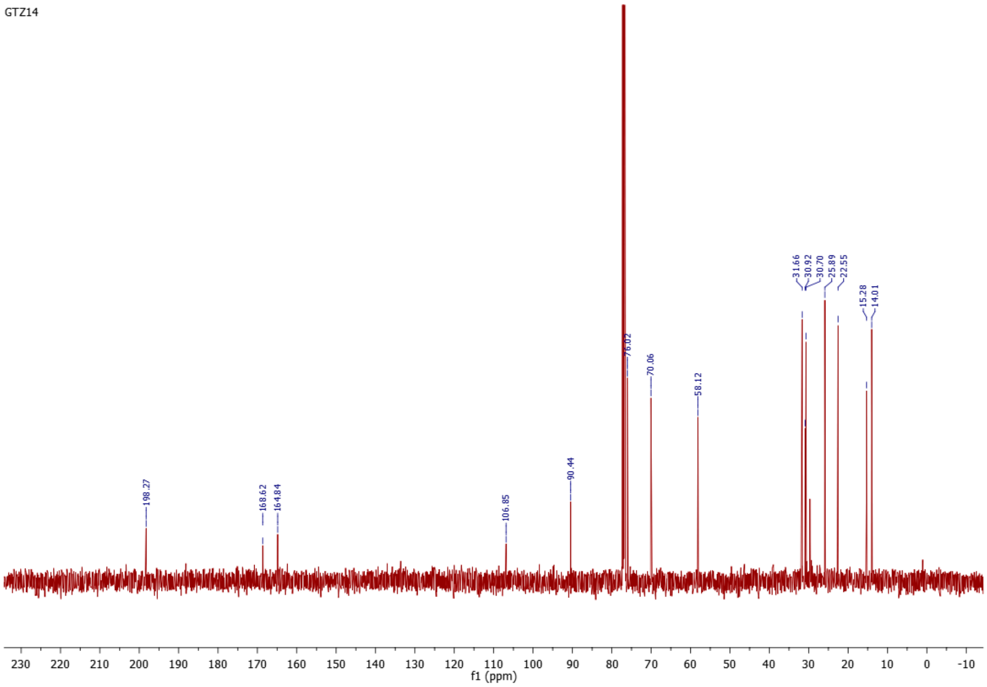

***Figure S5:*** $^{1}$H-NMR and $^{13}$C-NMR spectra for **25**

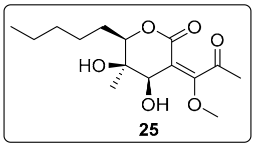


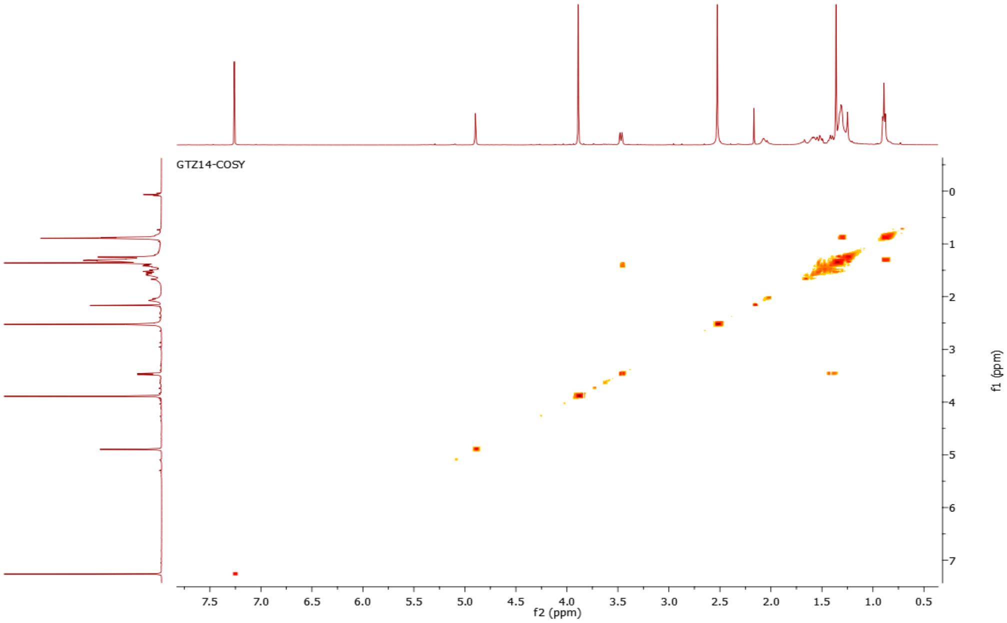


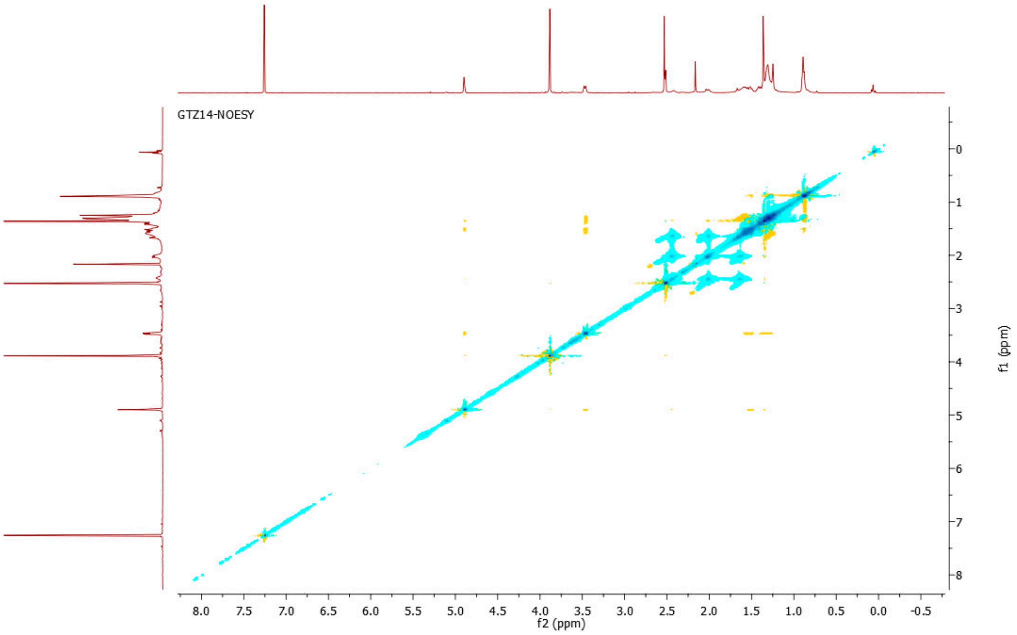


***Figure S6:*** $^{1}H$-$^{1}H$ COSY and $^{1}H$-$^{1}H$ NOESY spectra for **25**

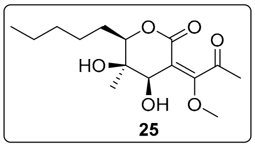


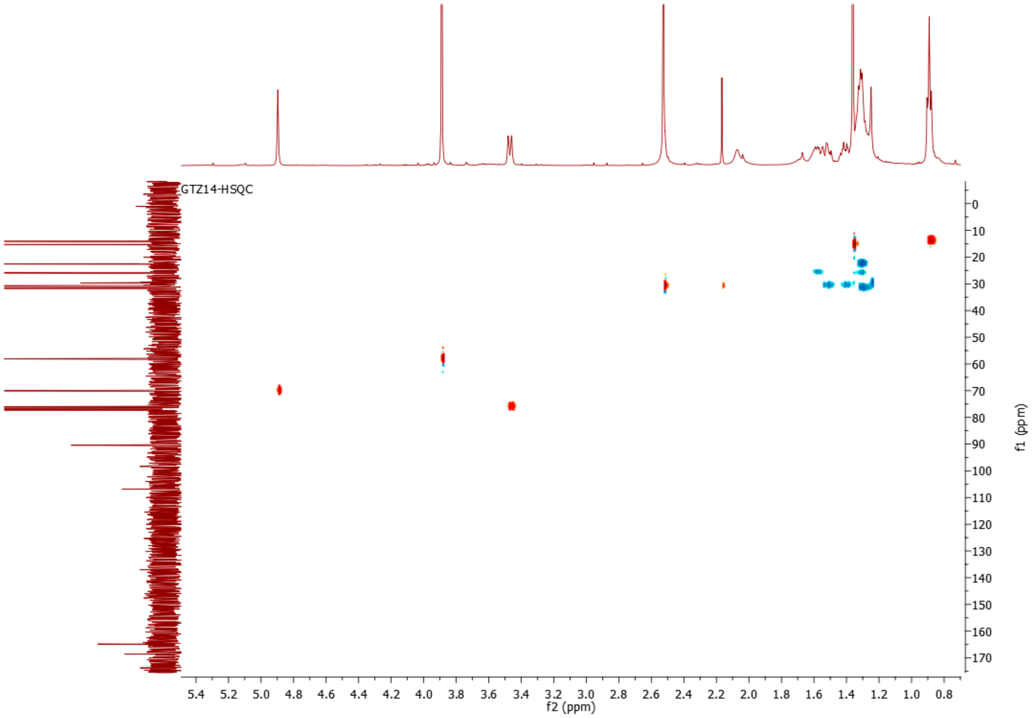


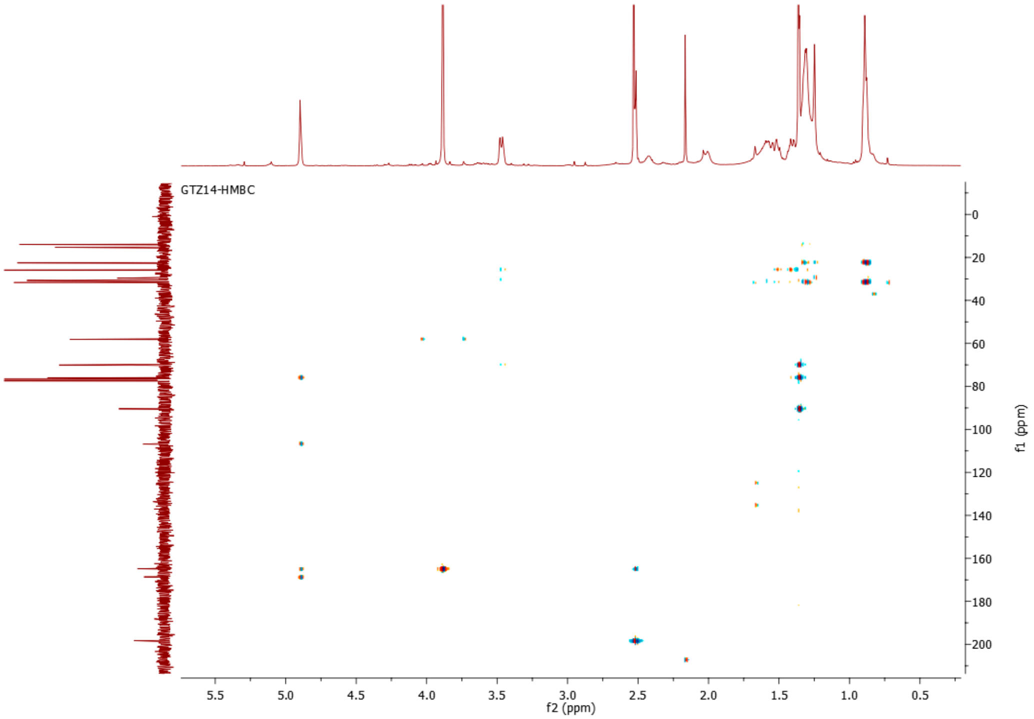


***Figure S7:*** $^1$H-$^{13}$C HSQC and $^1$H-$^{13}$C HMBC spectra for **25**

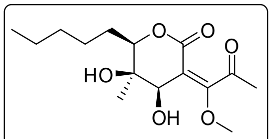


**25**

The compound **25** was eluted isocratically with methanol, with a retention time of 10.4 minutes. LC-MS analysis revealed a main peak with a relative area of 98.3 out of a total relative area of 100.0, corresponding to a calculated purity of 98.3%. Early eluting peaks corresponding to solvent front and impurities were excluded from the integration.

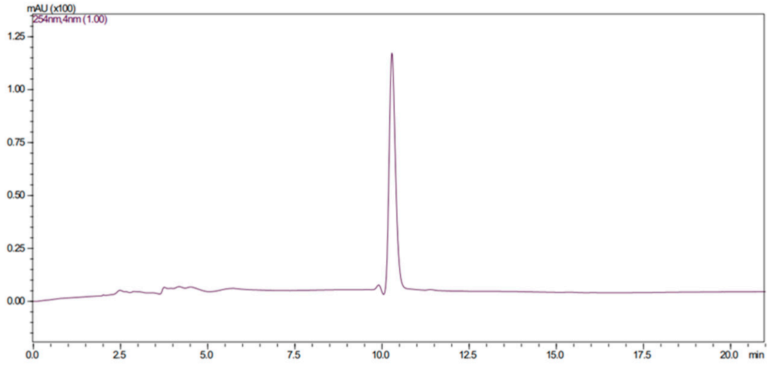


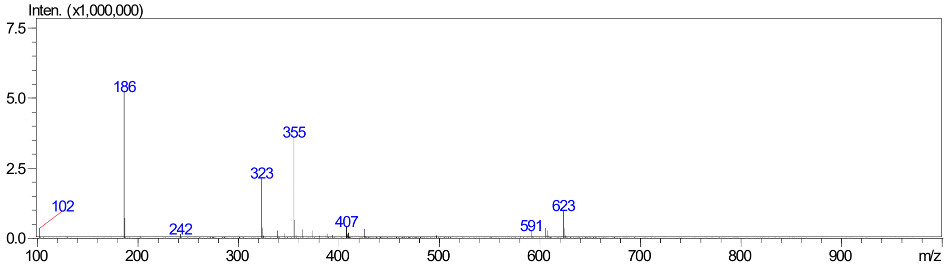


(-)

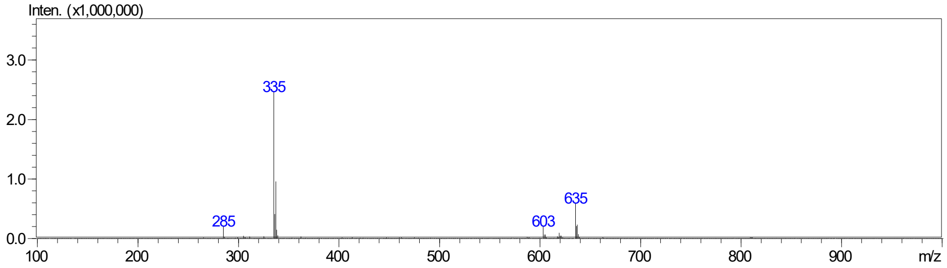


ESI-MS, positive mode: m/z calcd mass for $C_{15}H_{24}O_6$ $[M+Na]^+$ = 323.1471, was found 323.05.

***Figure S8:*** ESI-MS analysis of **25**

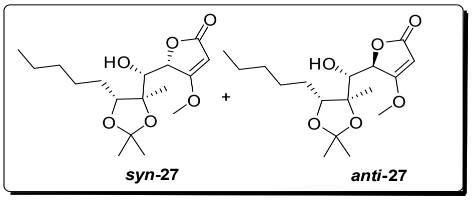


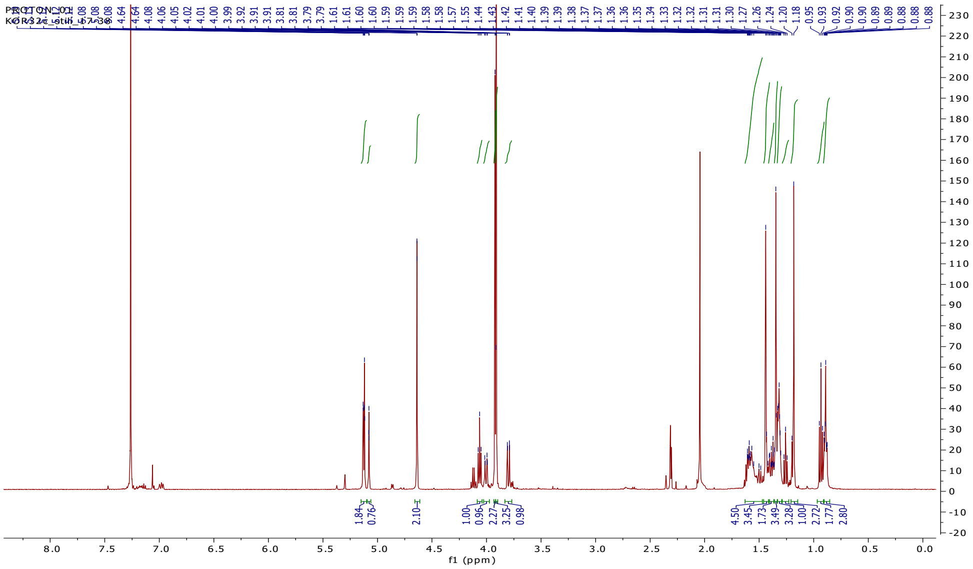


***Figure S9:*** $^{1}$H-NMR spectrum for *syn-,anti*-**27**

The compounds *syn-,anti*-**27** were eluted isocratically with methanol, with a retention time of 8.4 minutes. LC-MS analysis revealed a main peak with a relative area of 92.5 out of a total relative area of 100.0, corresponding to a calculated purity of 92.5%.

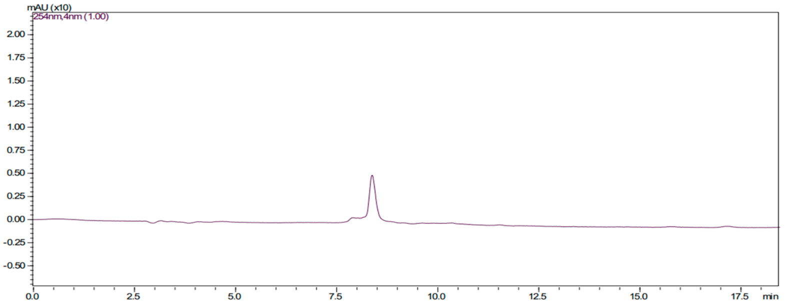


ESI-LCMS, positive mode: m/z calcd mass for $C_{17}H_{28}O_6$ $[M+Na]^+$ = 351.1784, was found 350.95

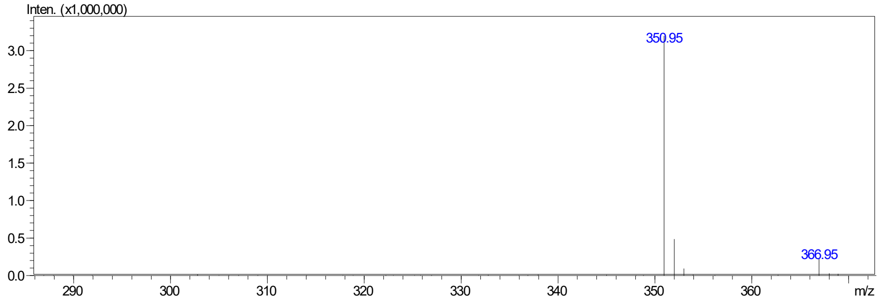


***Figure S10:*** ESI-LCMS analysis for *syn-,anti*-**27**

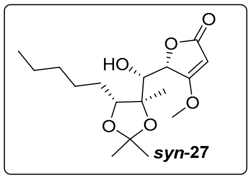


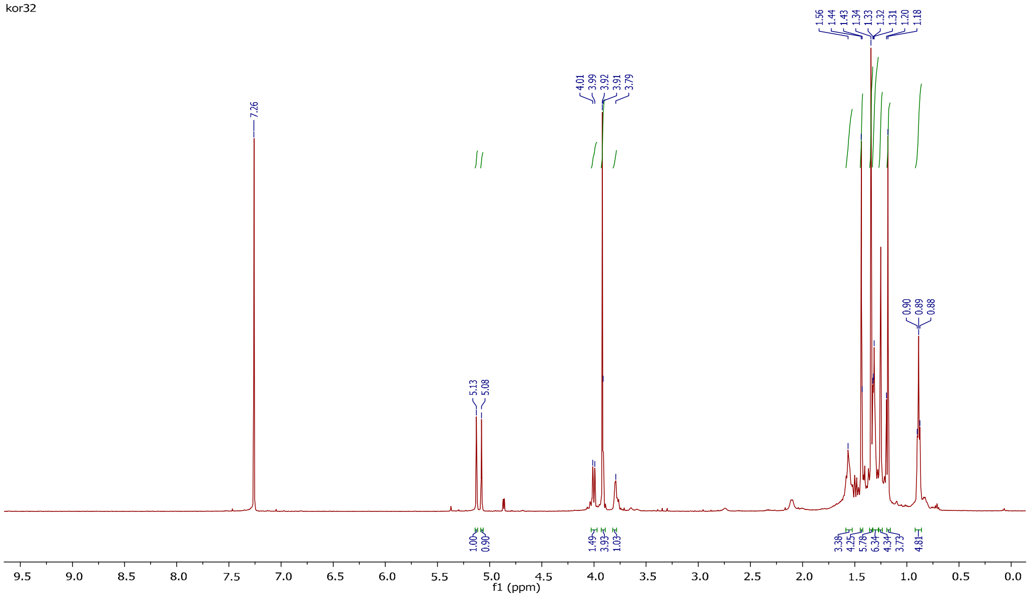


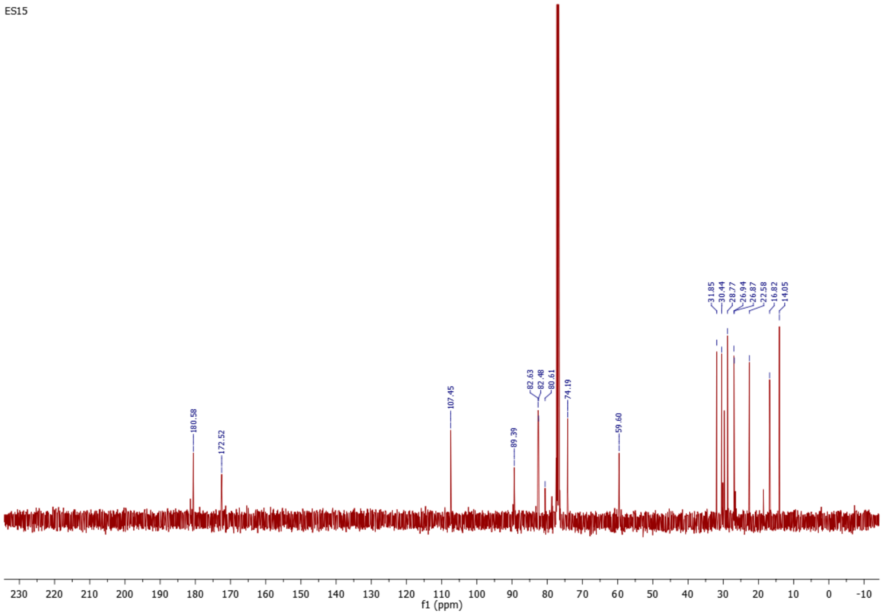


***Figure S11:*** $^{1}$H-NMR and $^{13}$C-NMR spectra for *syn*-**27**

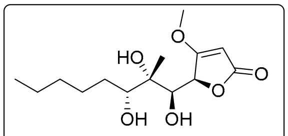

16

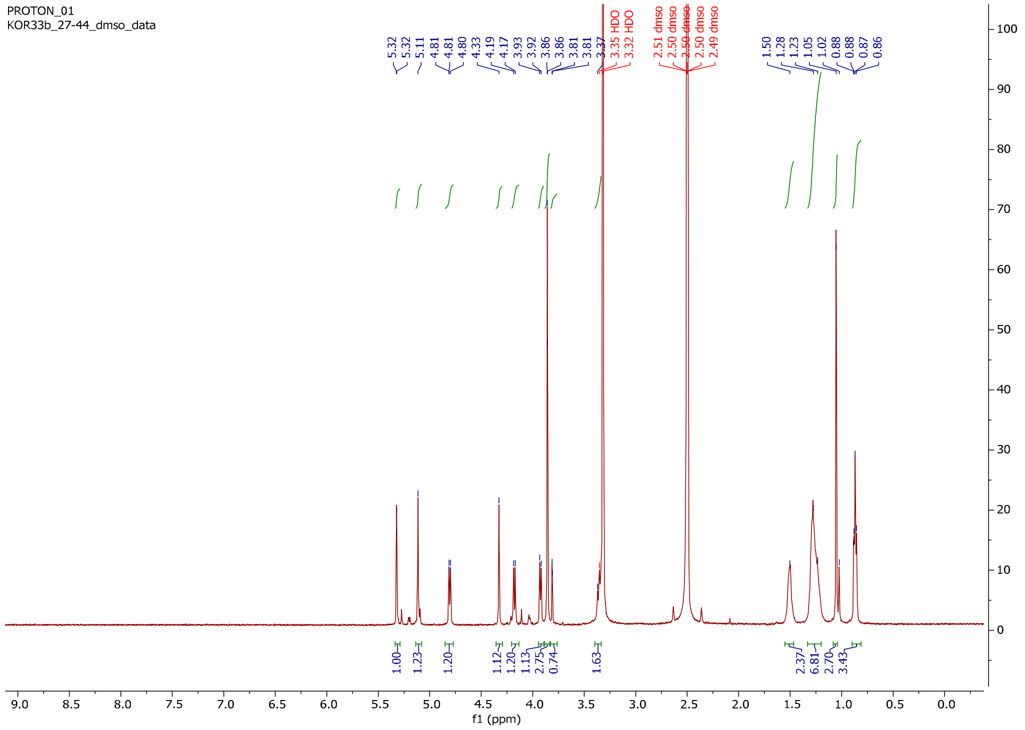


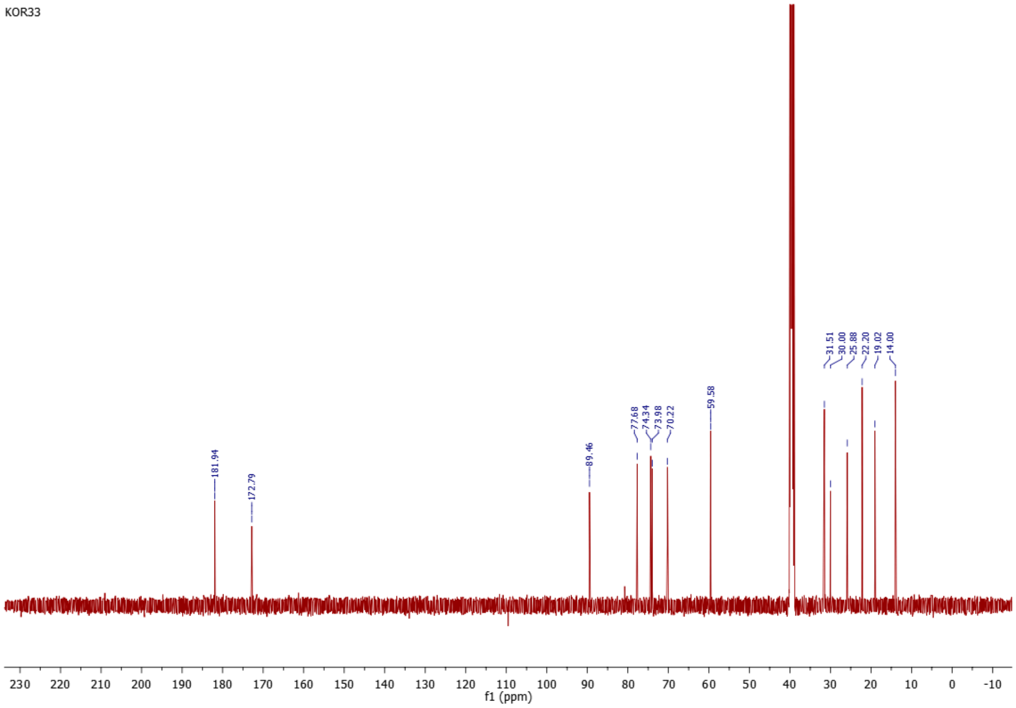


***Figure S12:*** $^{1}$H-NMR and $^{13}$C-NMR spectra for **16**

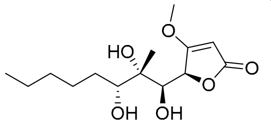


16

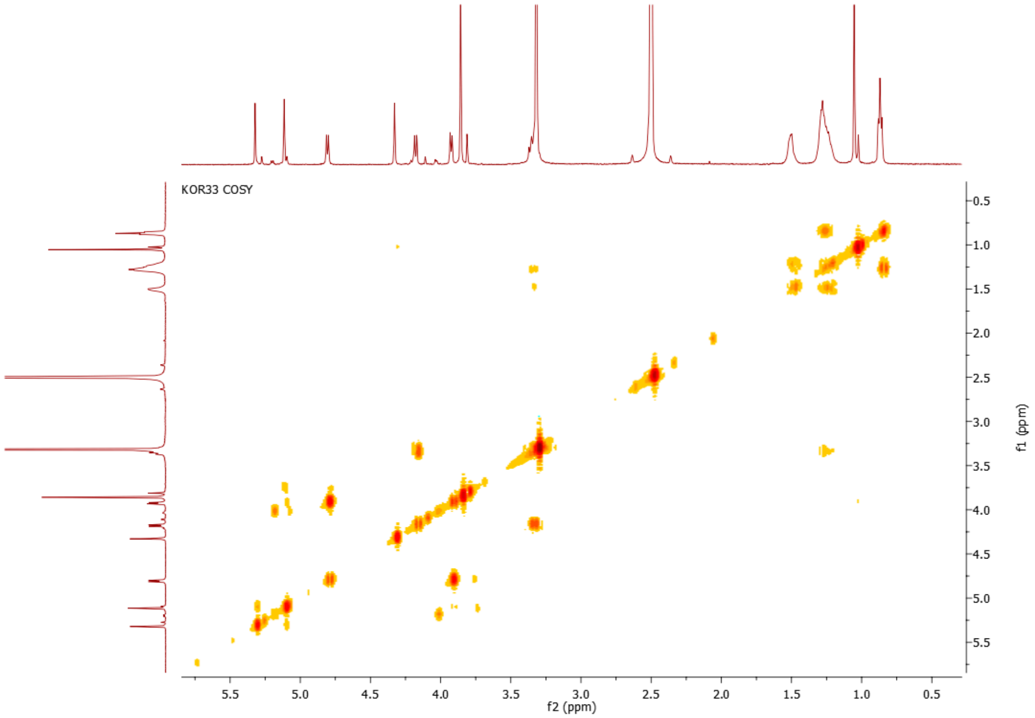


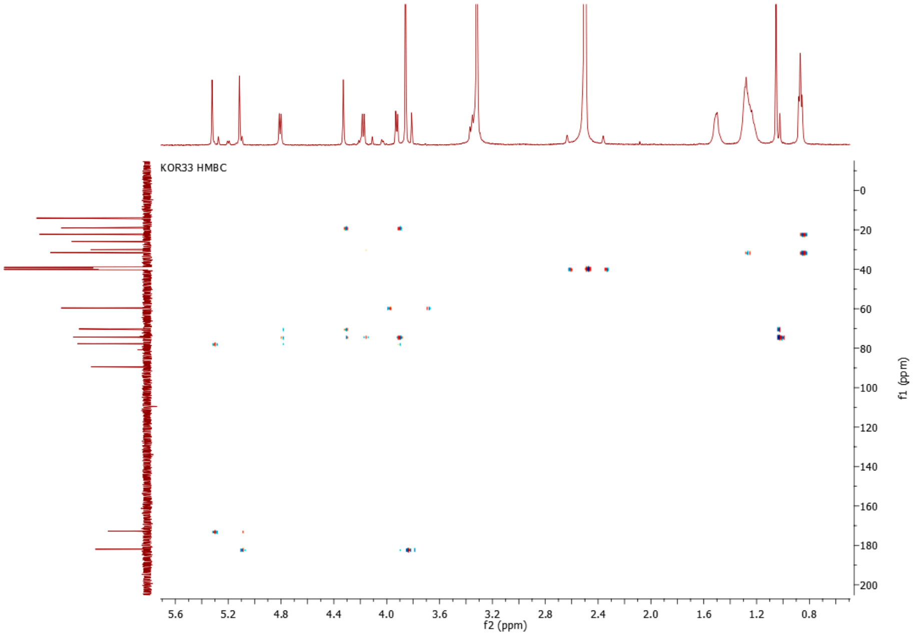


***Figure S13:*** $^{1}H$-$^{1}H$ COSY and $^{1}H$-$^{13}C$ HMBC spectra for **16**

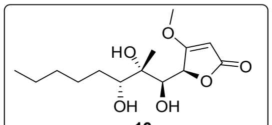


**16**

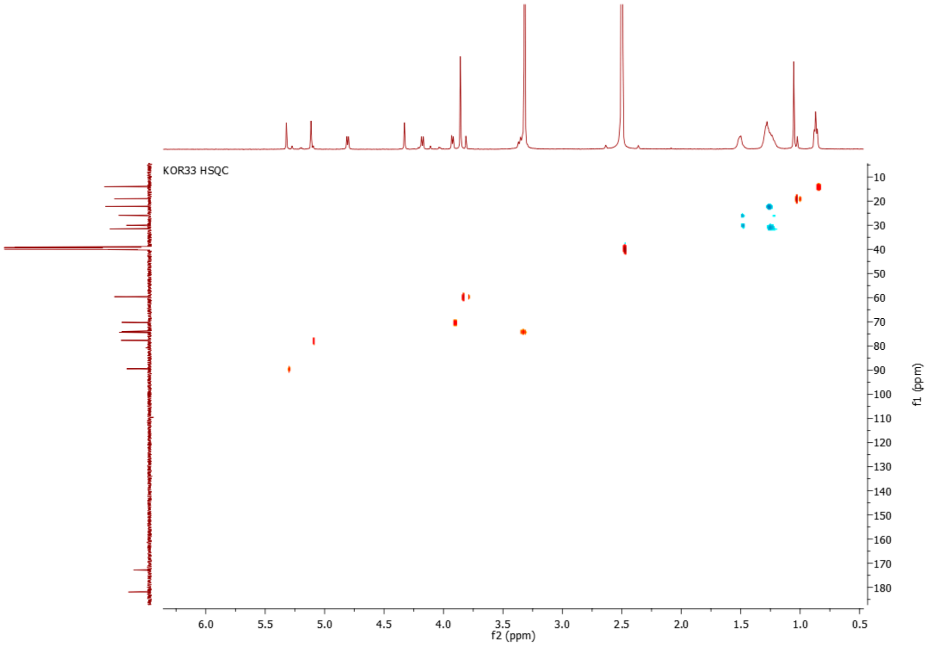


***Figure S14:*** $^{1}H$-$^{13}C$ HSQC spectrum **16**

The compound **16** was eluted isocratically with methanol, with a retention time of 8.8 minutes. LC-MS analysis revealed a main peak with a relative area of 94.1 out of a total relative area of 100.0, corresponding to a calculated purity of 94.1%.

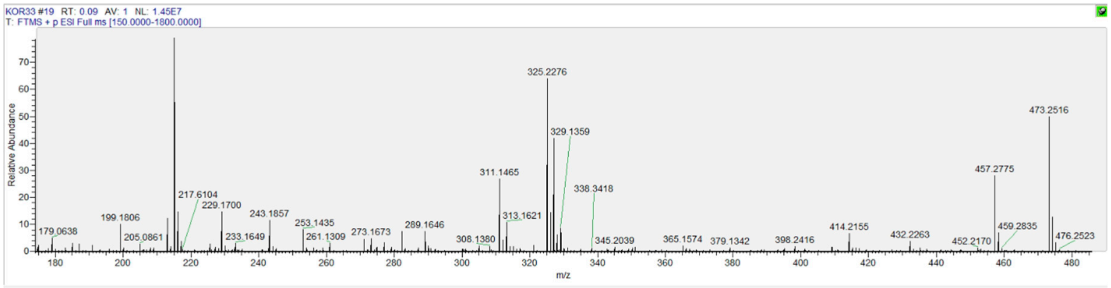


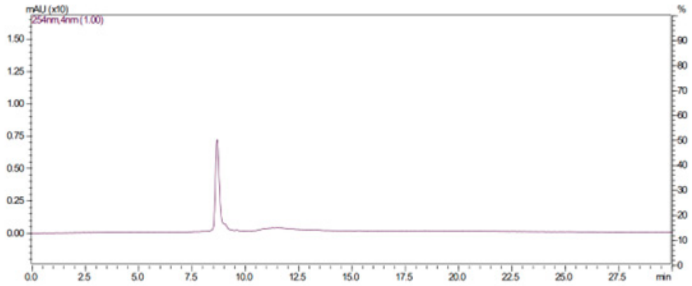


ESI-HRMS, positive mode: m/z calcd mass for $C_{14}H_{24}O_6$ $[M+Na]^+$ = 311.1471, was found 311.1465 for **16**

***Figure S15:*** ESI-LCMS for **16**

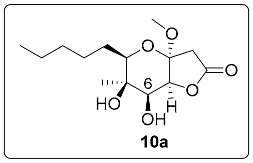

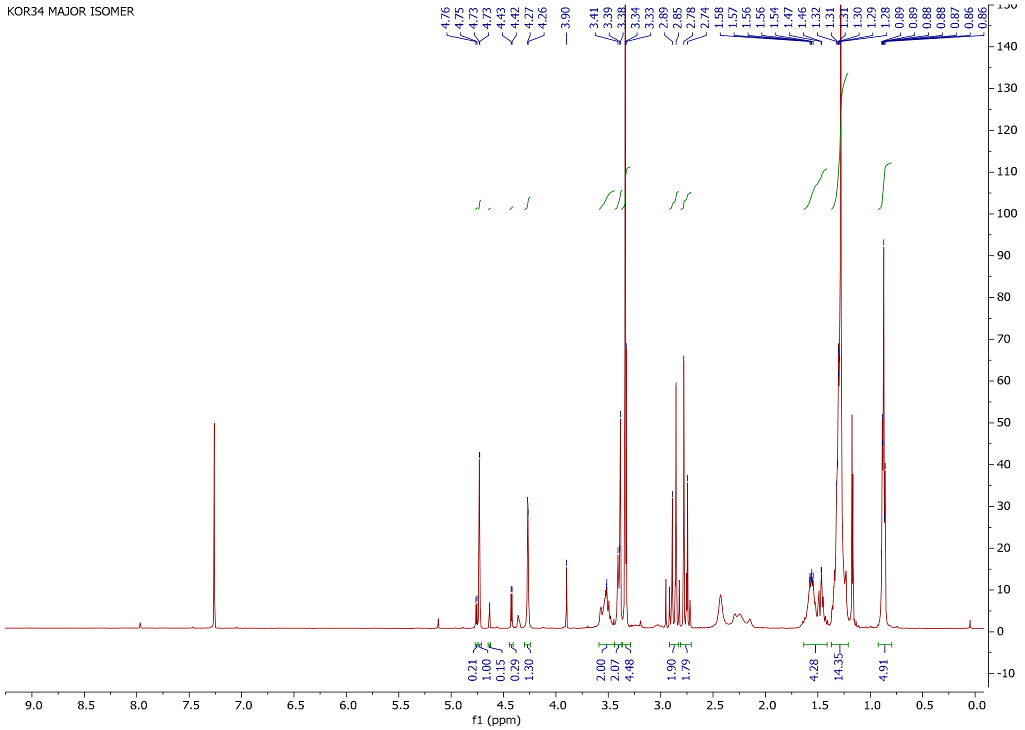


***Figure S16:*** $^{1}$H-NMR spectrum for **10a**

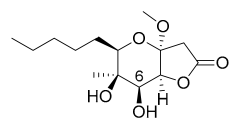

**10a**

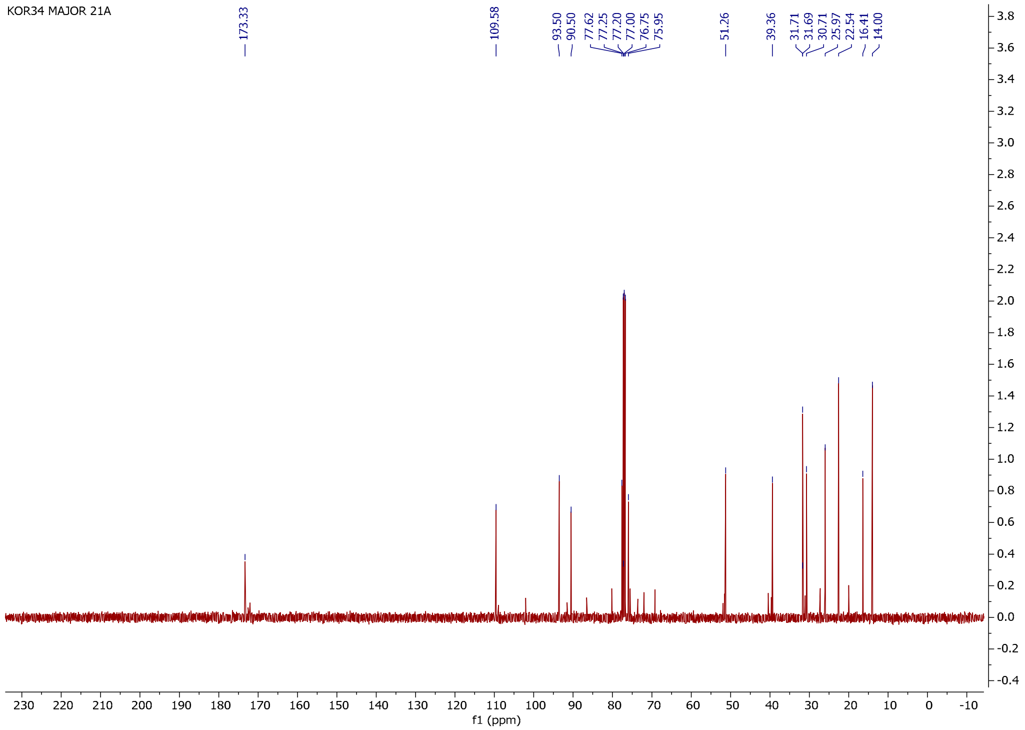


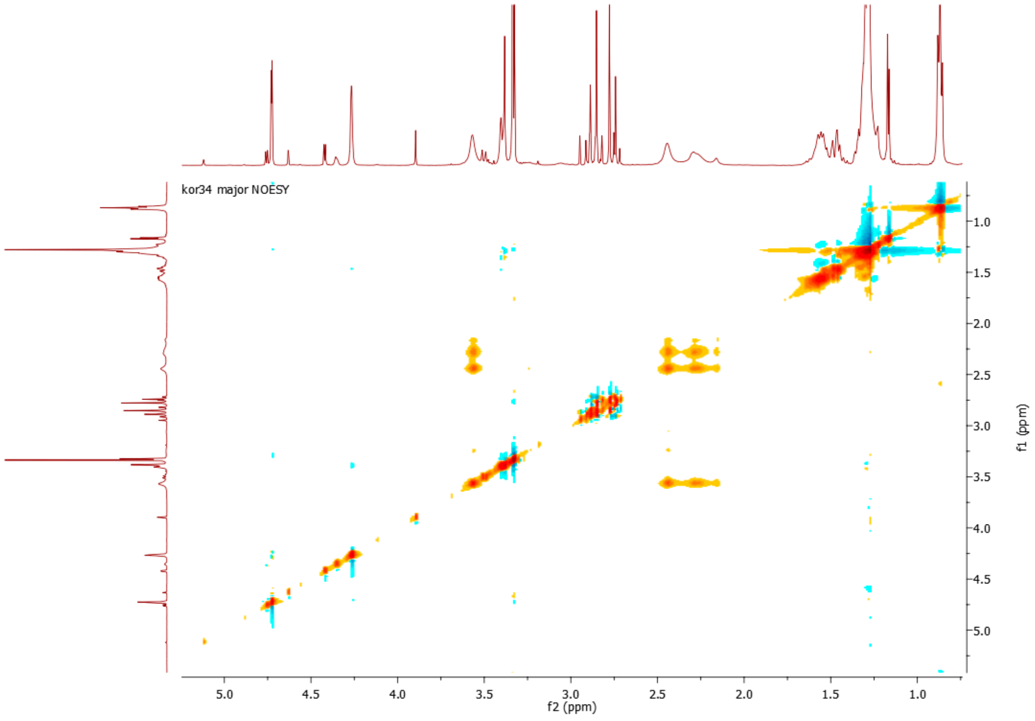


***Figure S17:*** $^{13}$C-NMR and $^{1}$H-$^{1}$H NOESY spectra for **10a**

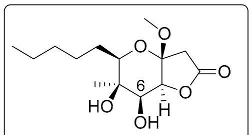

**10b**

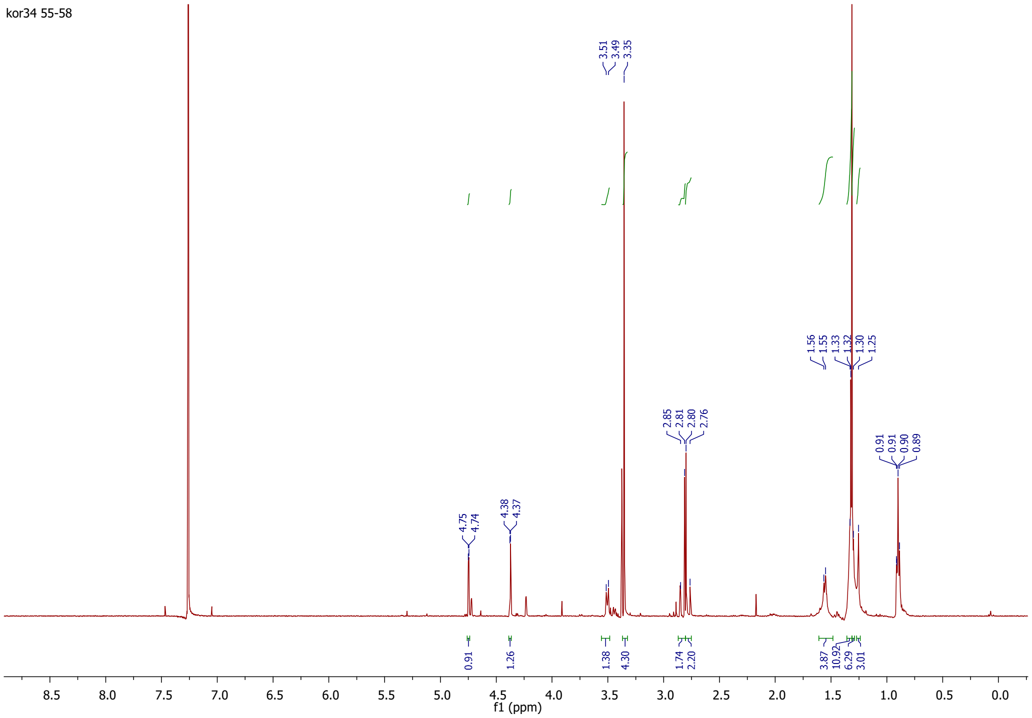


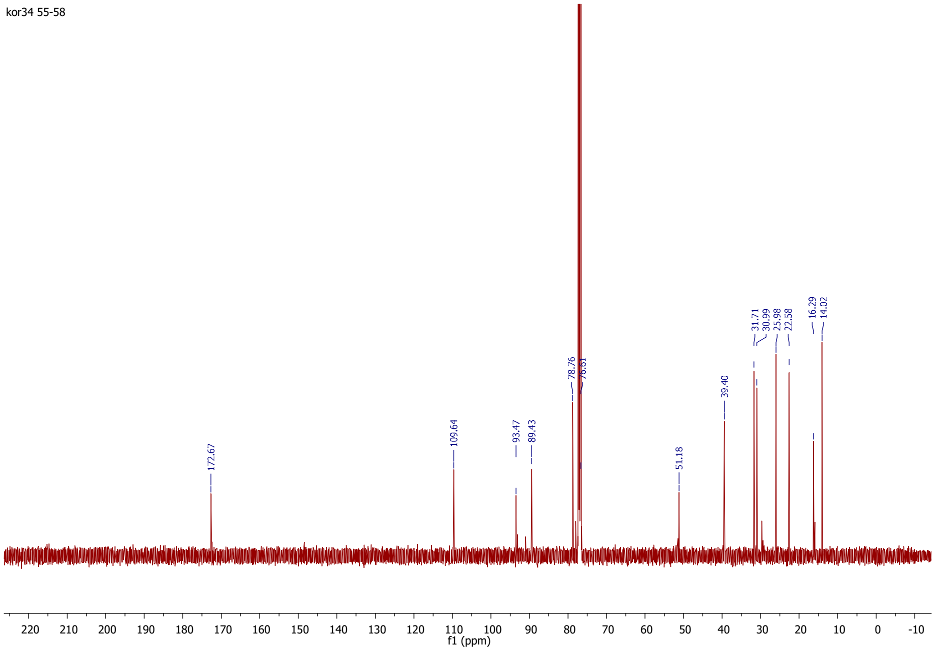


***Figure S18:*** $^{1}$H-NMR and $^{13}$C-NMR spectra for **10b**

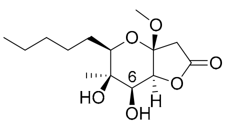


**10b**

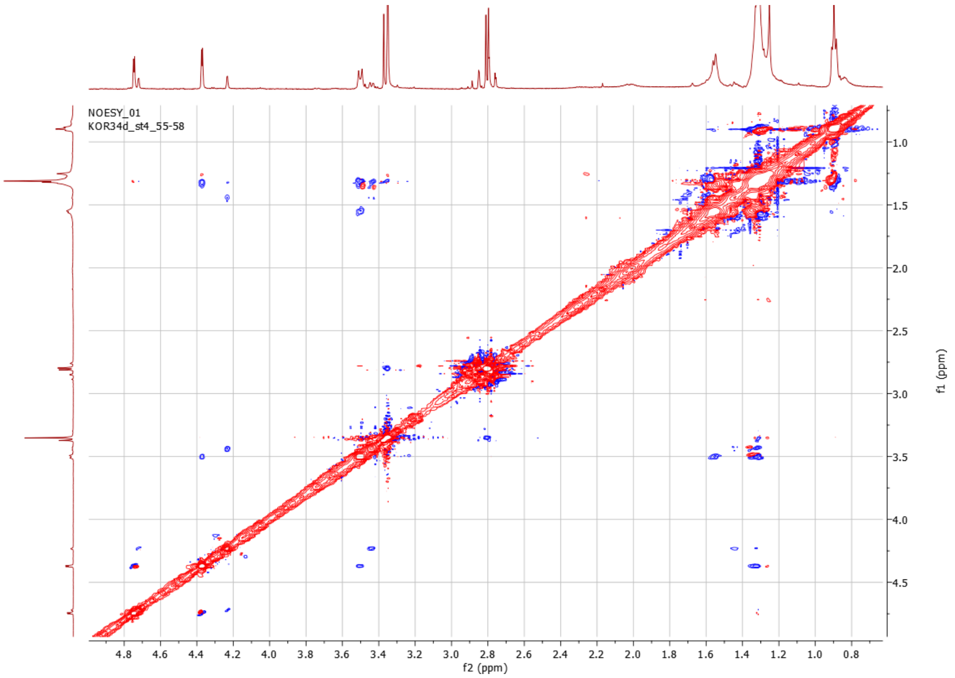


***Figure S19:*** $^{1}$H-$^{1}$H NOESY spectra for **10b**

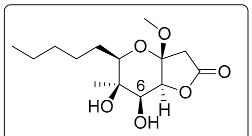


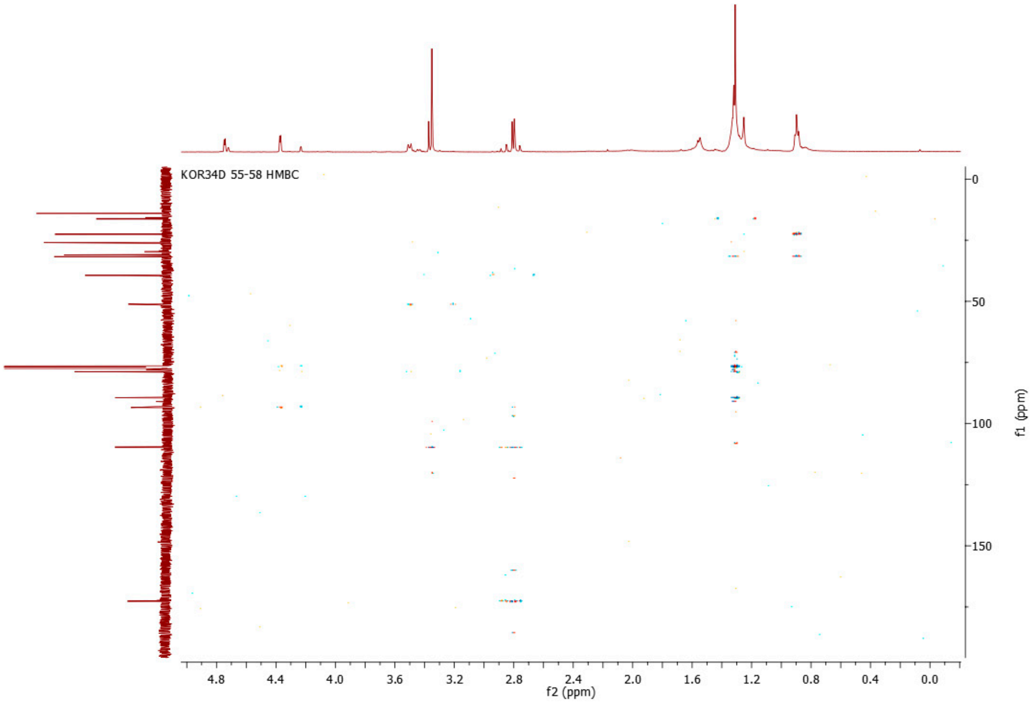


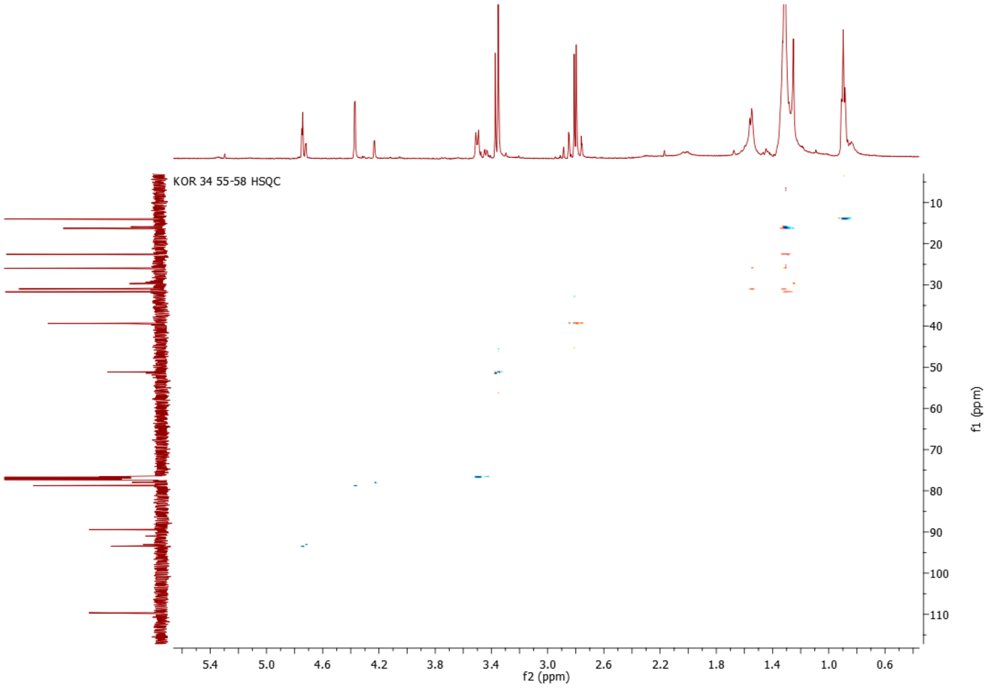


***Figure S20:*** $^{1}H$-$^{13}C$ HMBC and $^{1}H$-$^{13}C$ HSQC spectra for **10b**

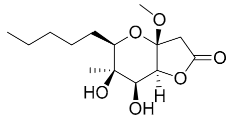


**10b**

The compound **10b** was eluted isocratically with methanol, with a retention time of 9.1 minutes. LC-MS analysis revealed a main peak with a relative area of 94.6 out of a total relative area of 100.0, corresponding to a calculated purity of 94.6%.

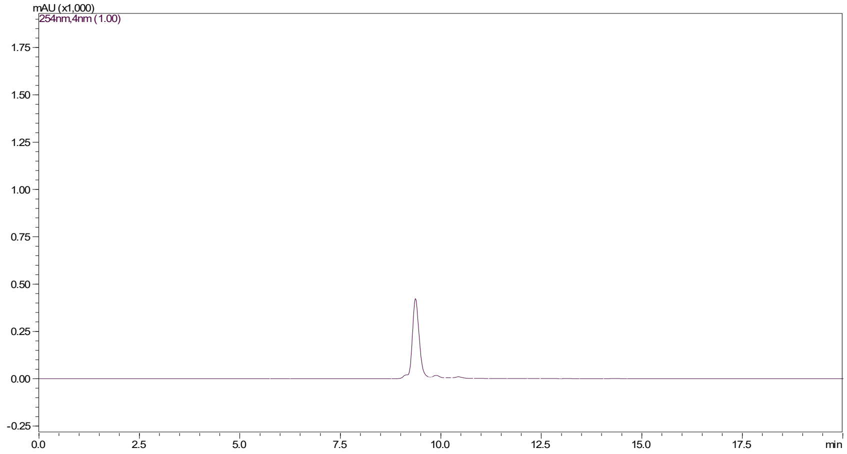


ESI-MS, positive mode: m/z calcd mass for $C_{14}H_{24}NaO_6$ $[M+Na]^+$ = 311.1471, was found 310.90

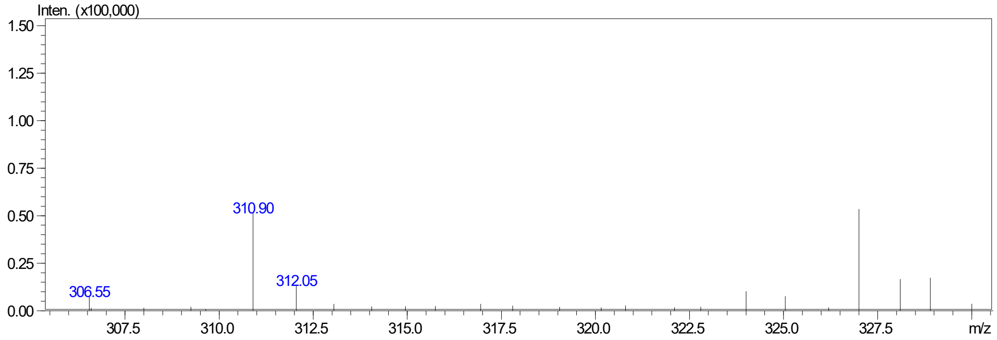


***Figure S21:*** ESI-LCMS for **10b**

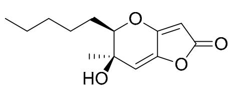


**28**

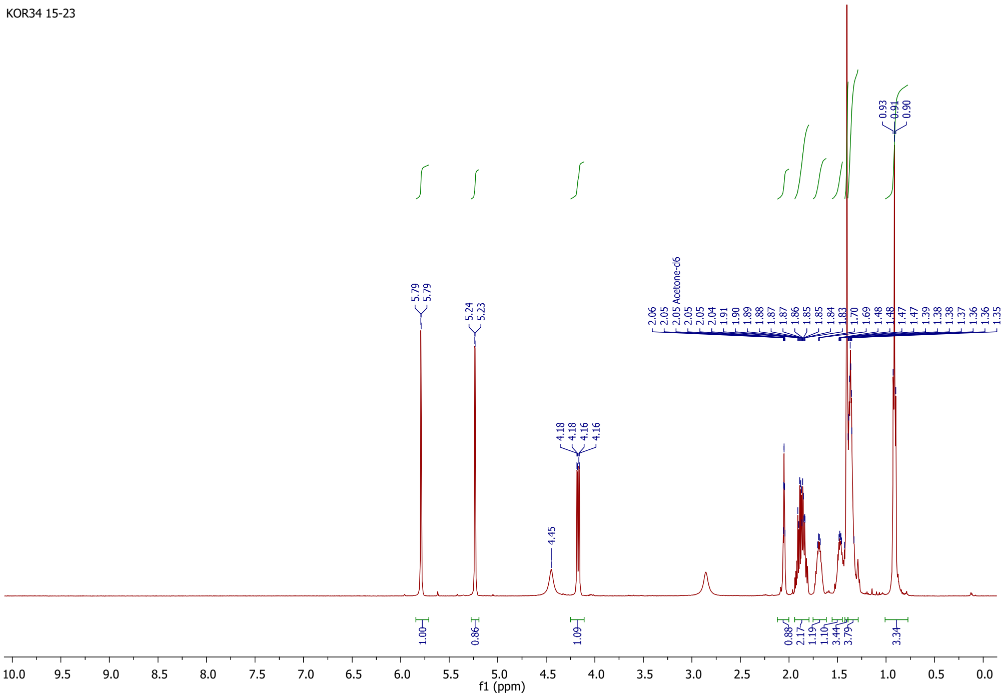


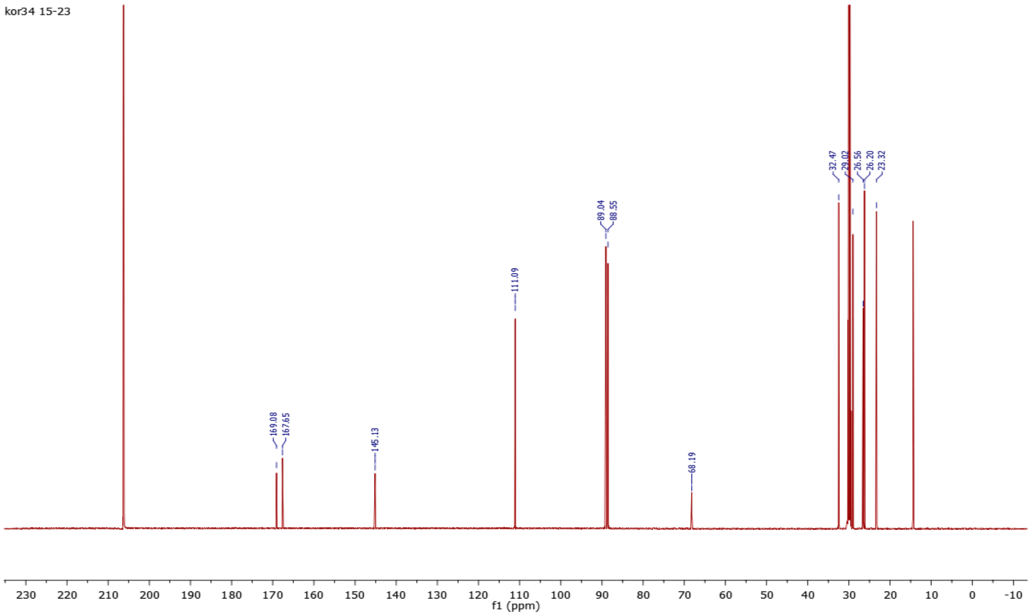


***Figure S22:*** $^{1}$H-NMR and $^{13}$C-NMR spectra for **28**

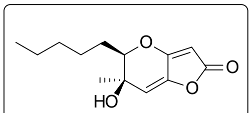


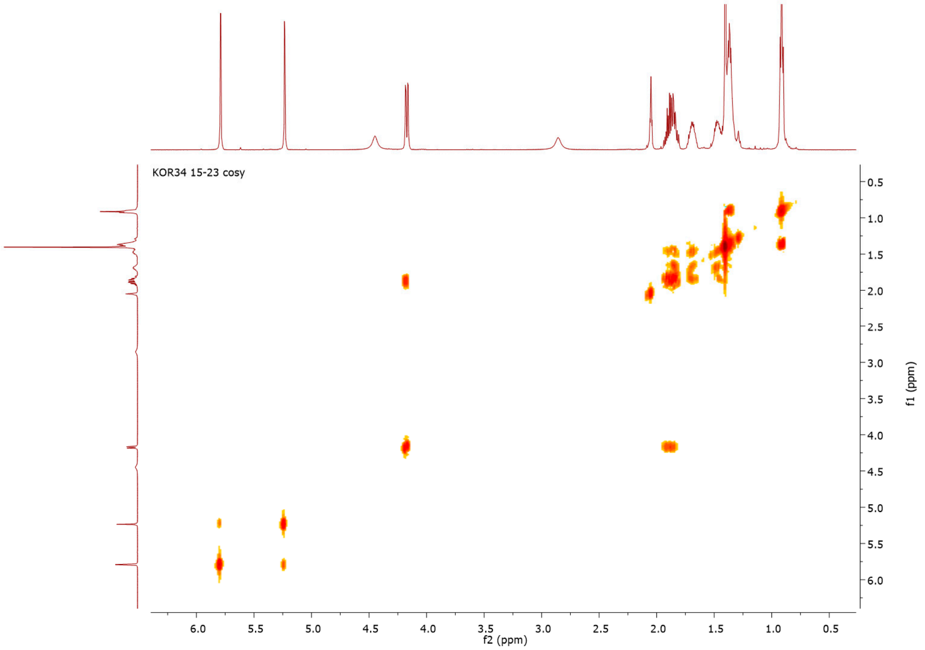


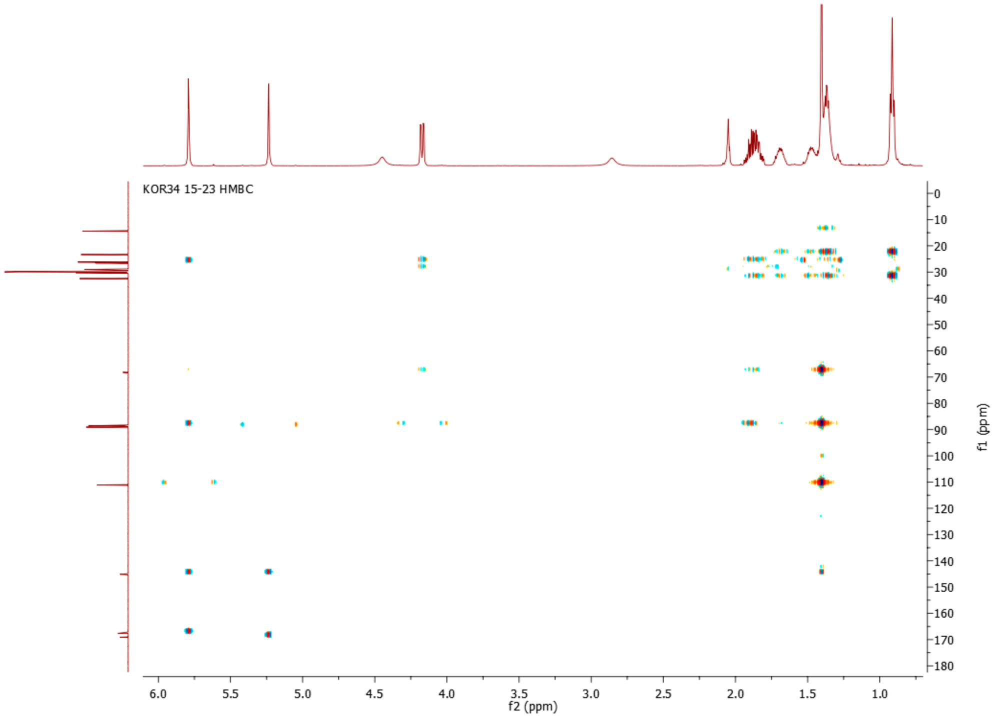


***Figure S23:*** $^1H$-$^1H$ COSY and $^1H$-$^{13}C$ HMBC spectra for **28**

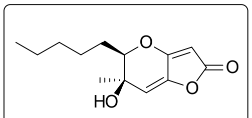

**28**

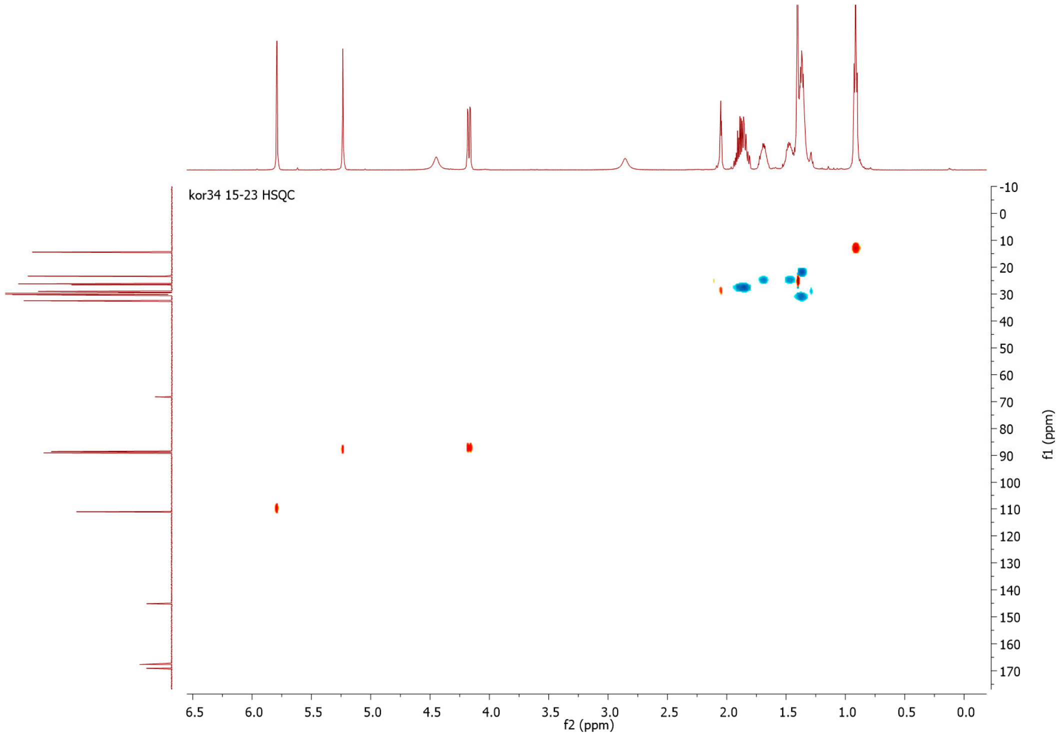


***Figure S24:*** $^1$H-$^{13}$C HSQC spectrum for **28**

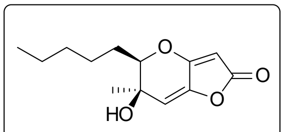

**28**

The compound **28** was eluted isocratically with methanol, with a retention time of 9.6 minutes. LC-MS analysis revealed a main peak with a relative area of 98.6 out of a total relative area of 100.0, corresponding to a calculated purity of 98.6%.

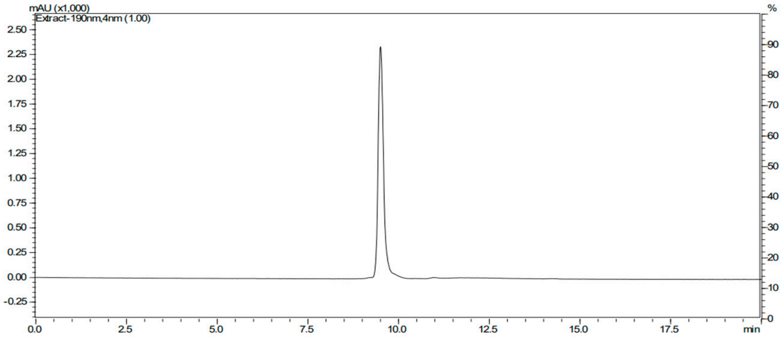


ESI-MS, negative mode: m/z calcd mass for $C_{13}H_{18}O_4$ $[M-H]^-$ = 237.1127, was found 236.90

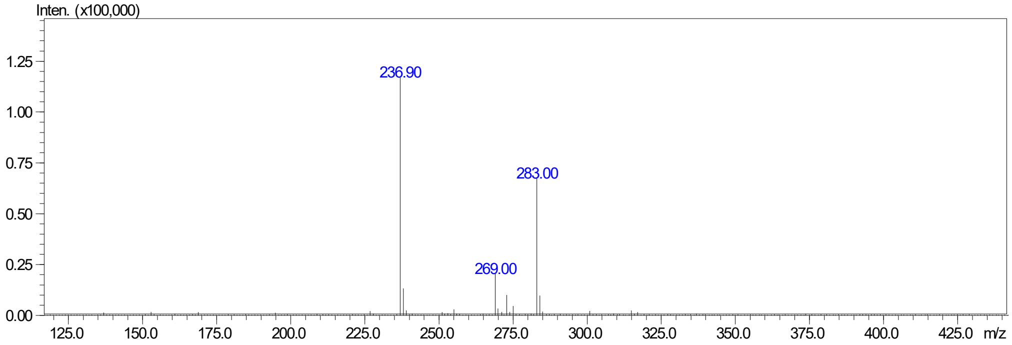


***Figure S25:*** ESI-LCMS for **28**

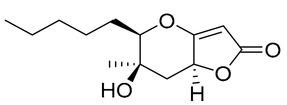

**9**

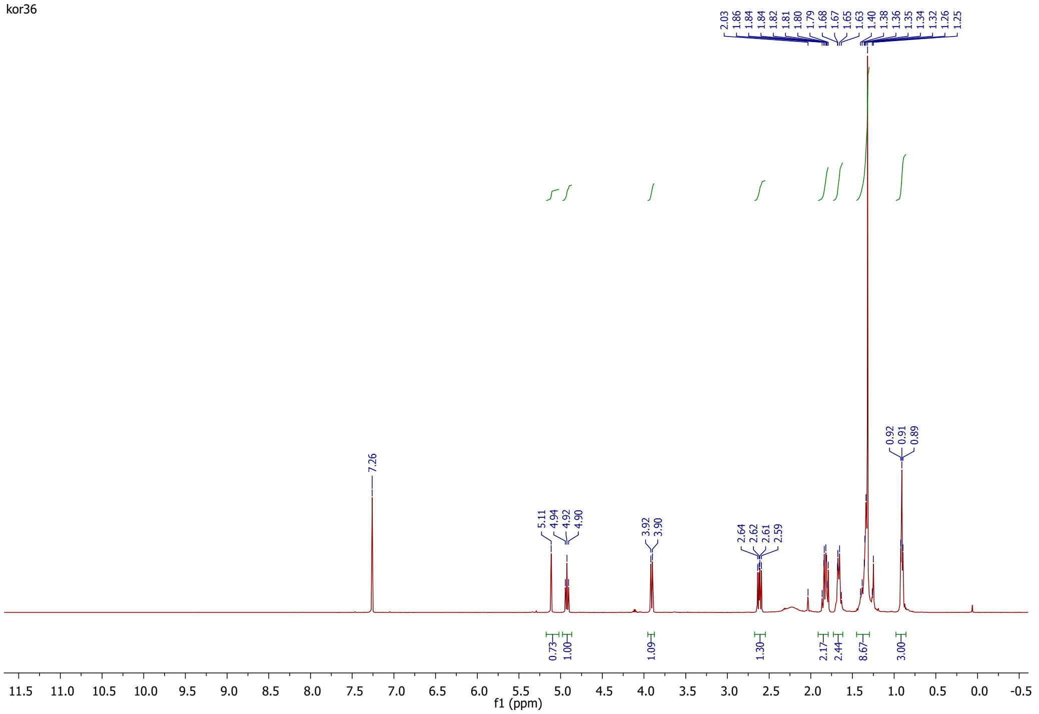


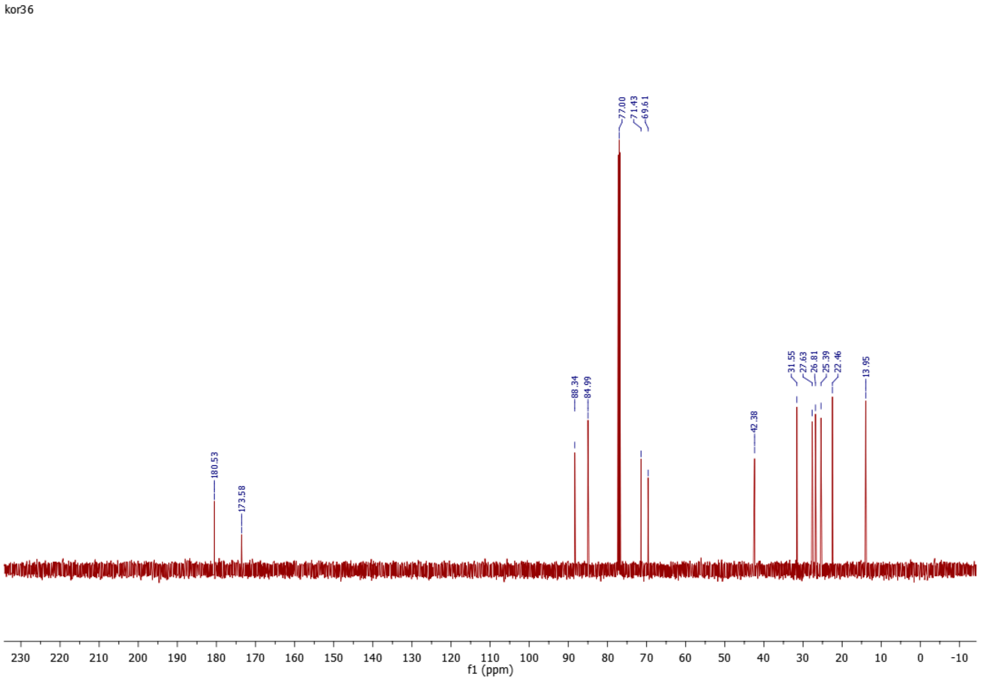


***Figure S26:*** $^{1}H$-NMR and $^{13}C$-NMR spectra for **9**

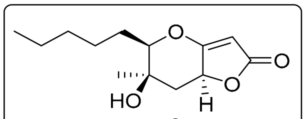


**9**

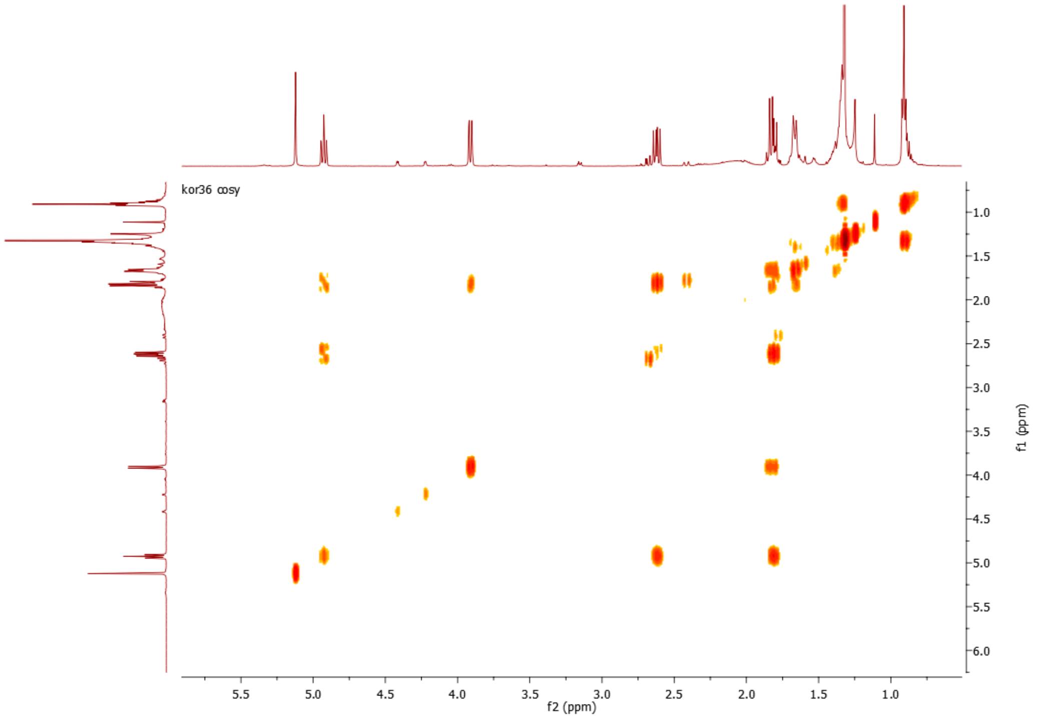


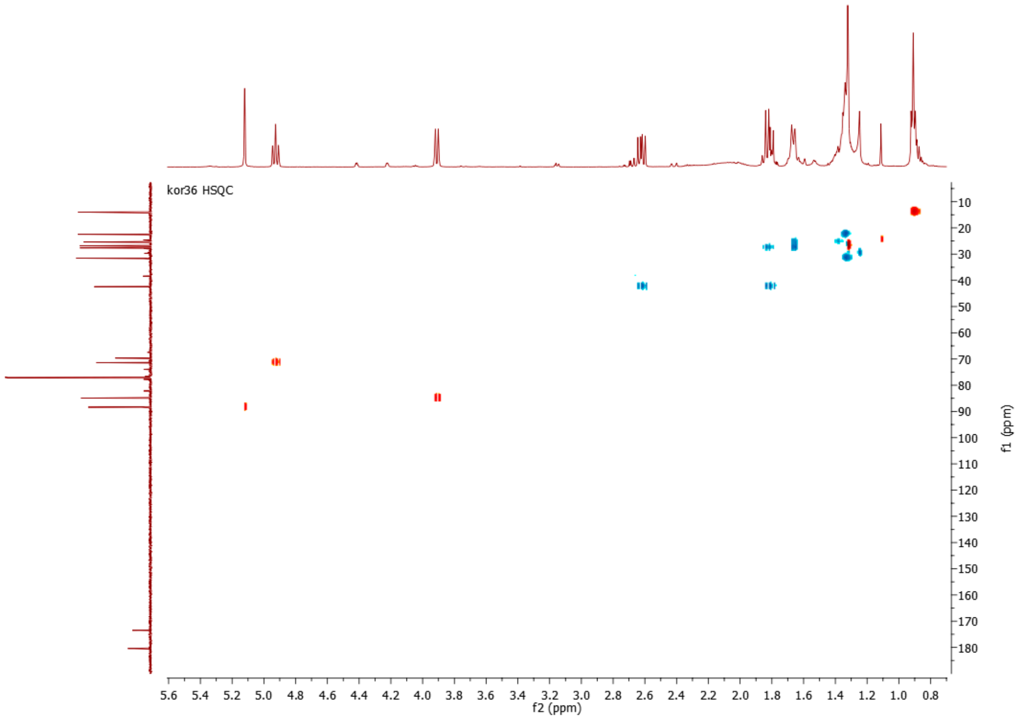


***Figure S27:*** $^{1}$H-$^{1}$H COSY and $^{1}$H-$^{13}$C HSQC spectra for **9**

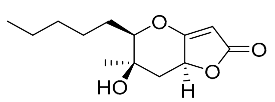


**9**

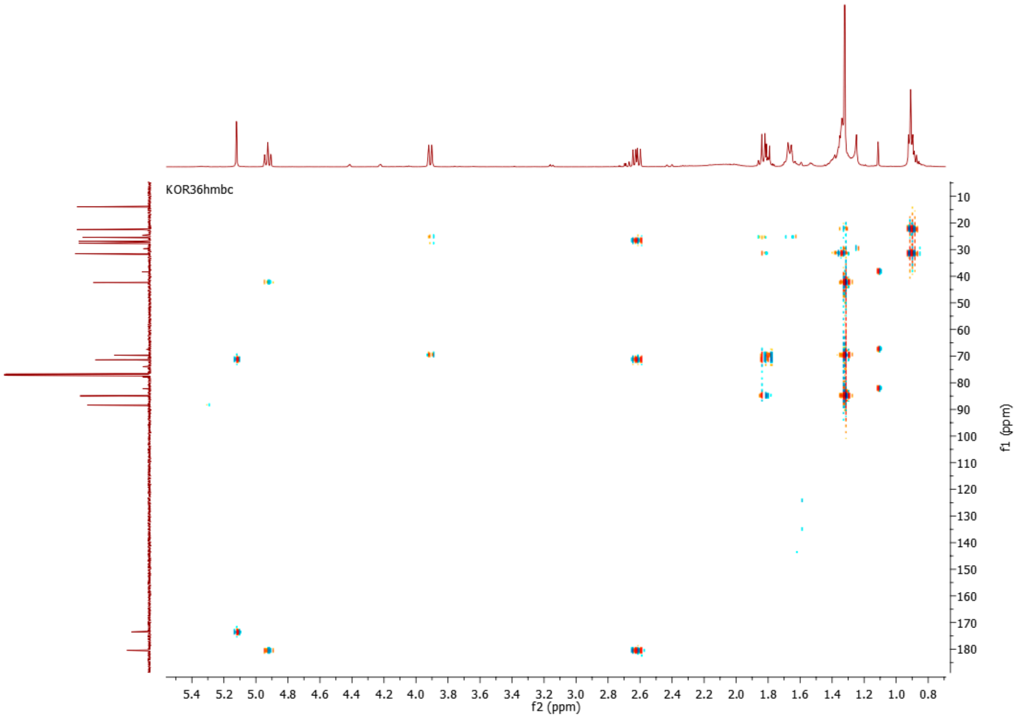


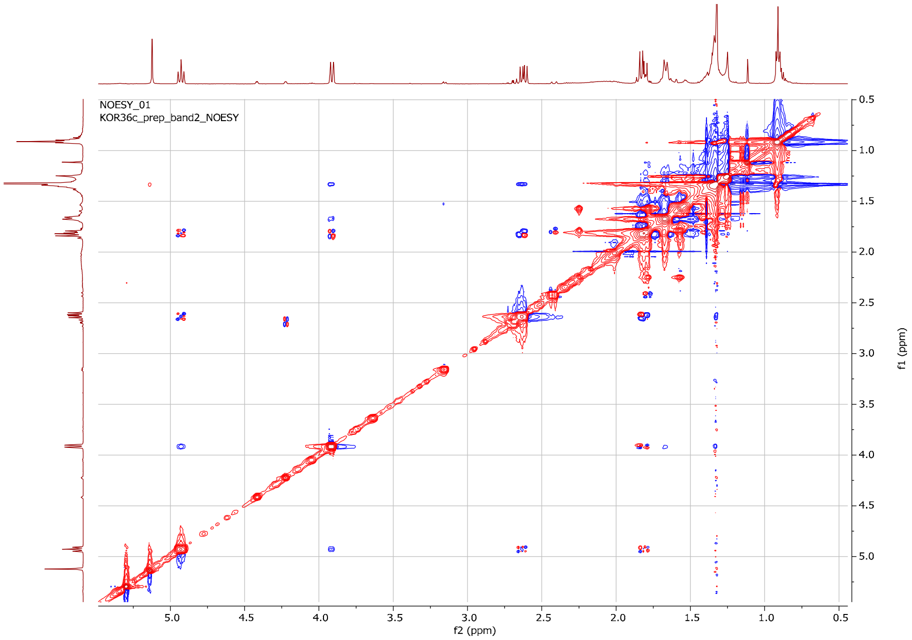


***Figure S28:*** $^{1}$H-$^{13}$C HMBC and $^{1}$H-$^{1}$H NOESY spectra for **9**

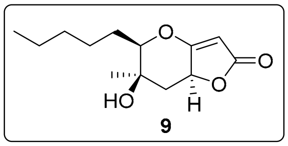

The compound **9** was eluted isocratically with methanol, with a retention time of 8.7 minutes. LC-MS analysis revealed a main peak with a relative area of 93.9 out of a total relative area of 100.0, corresponding to a calculated purity of 93.9%. Early eluting peaks corresponding to solvent front and impurities were excluded from the integration.

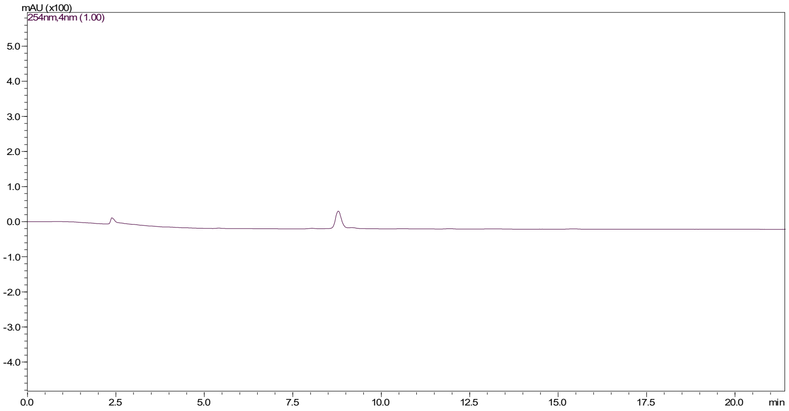


ESI-LCMS, positive mode: m/z calcd mass for $C_{13}H_{20}O_4$ $[M+Na]^+$ = 263.1259, was found 263.15

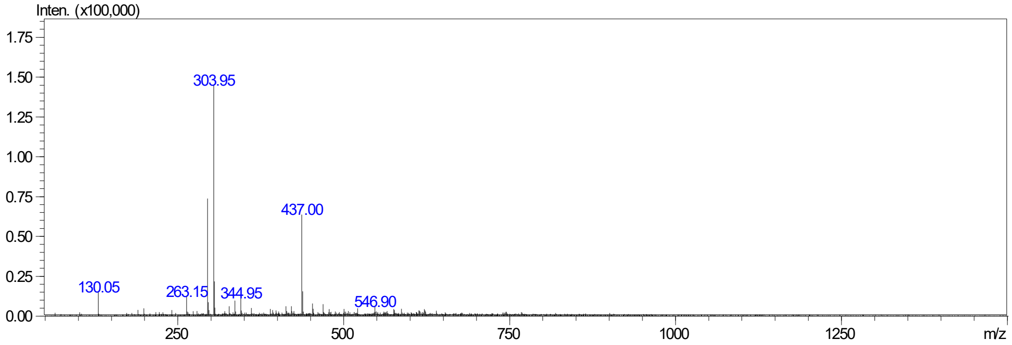


***Figure S29:*** ESI-LCMS analysis of **9**

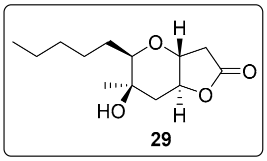


29

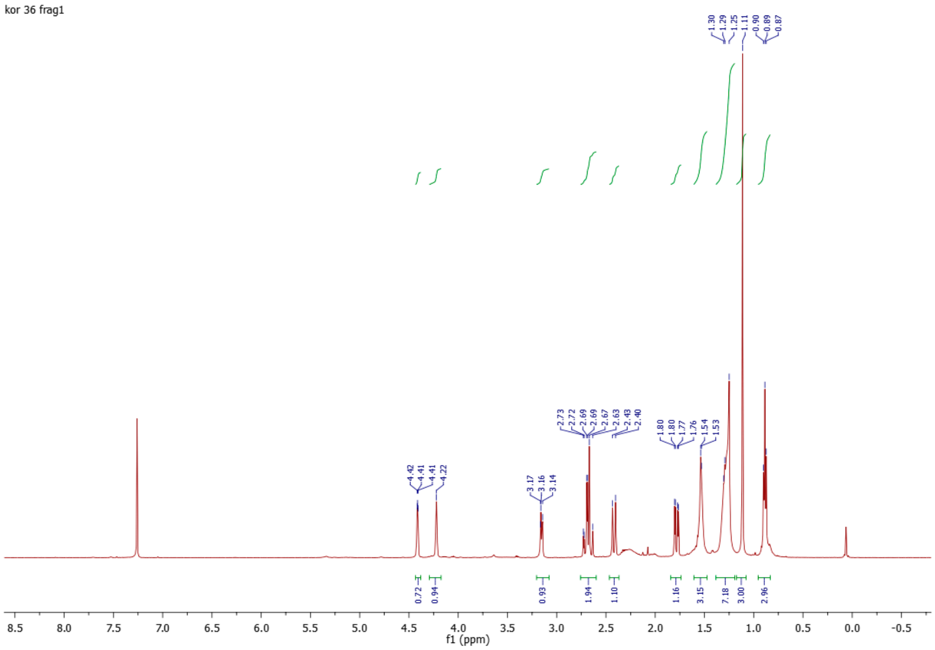


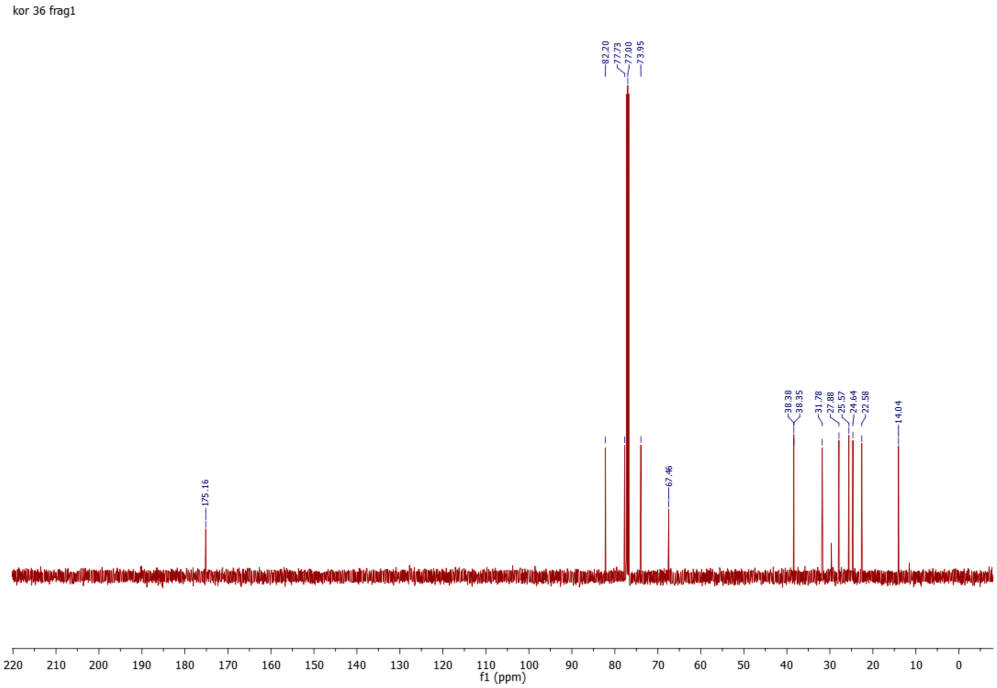


***Figure S30:*** $^{1}$H-NMR and $^{13}$C-NMR spectra for **29**

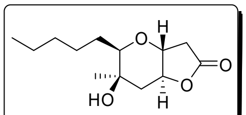


**29**

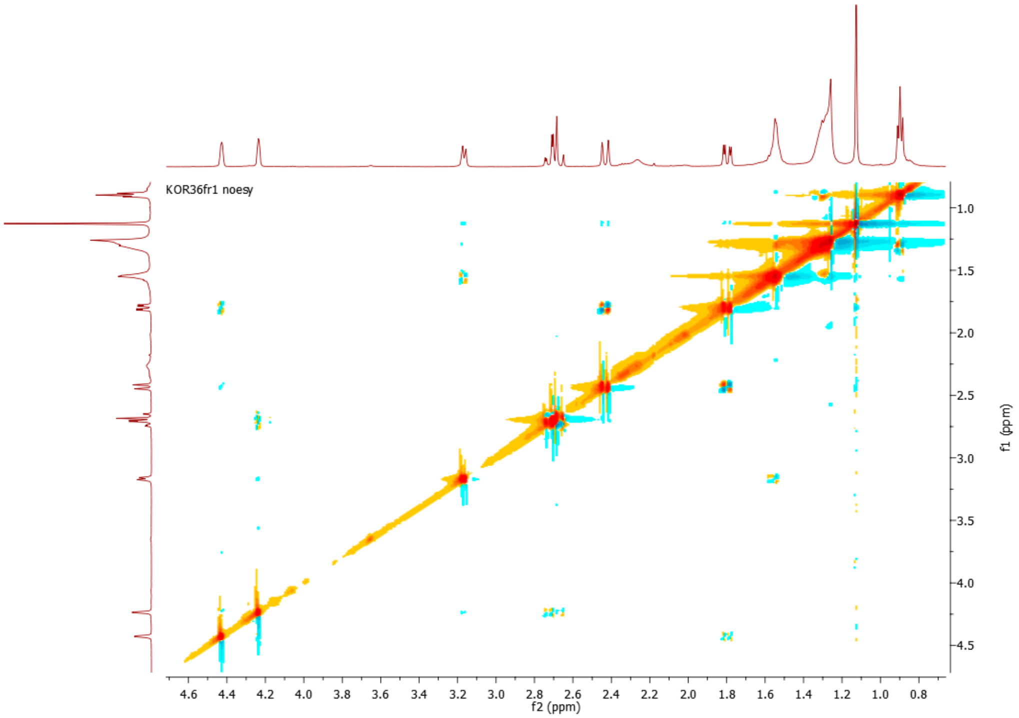


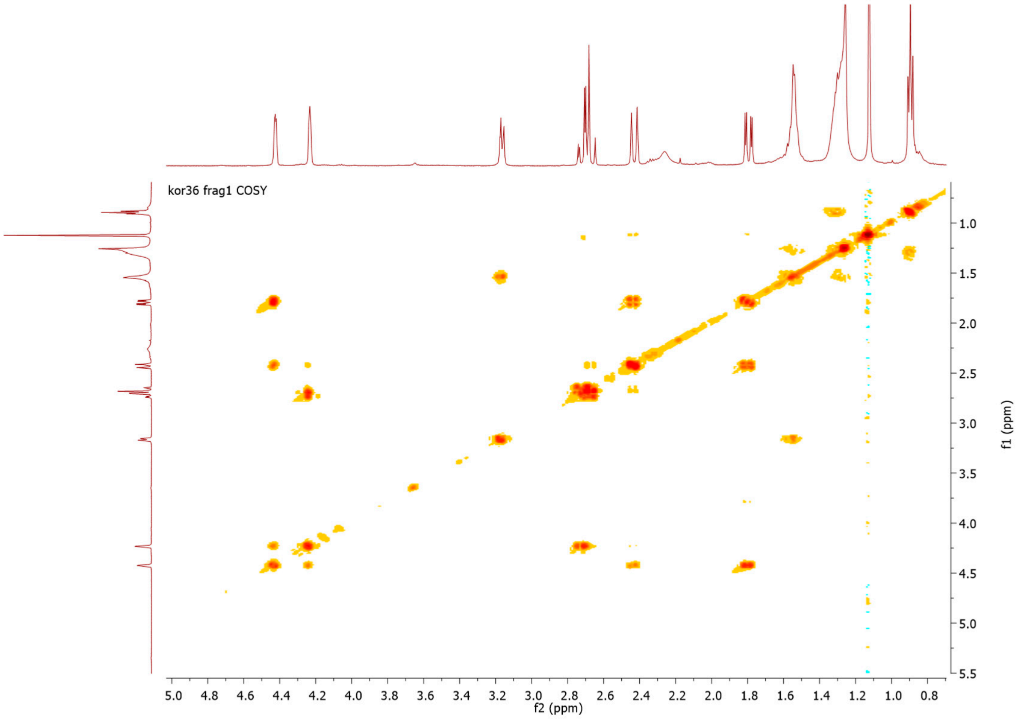


***Figure S31:*** $^{1}$H-$^{1}$H COSY and $^{1}$H-$^{1}$H NOESY spectra for **29**

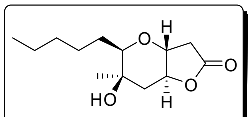


**29**

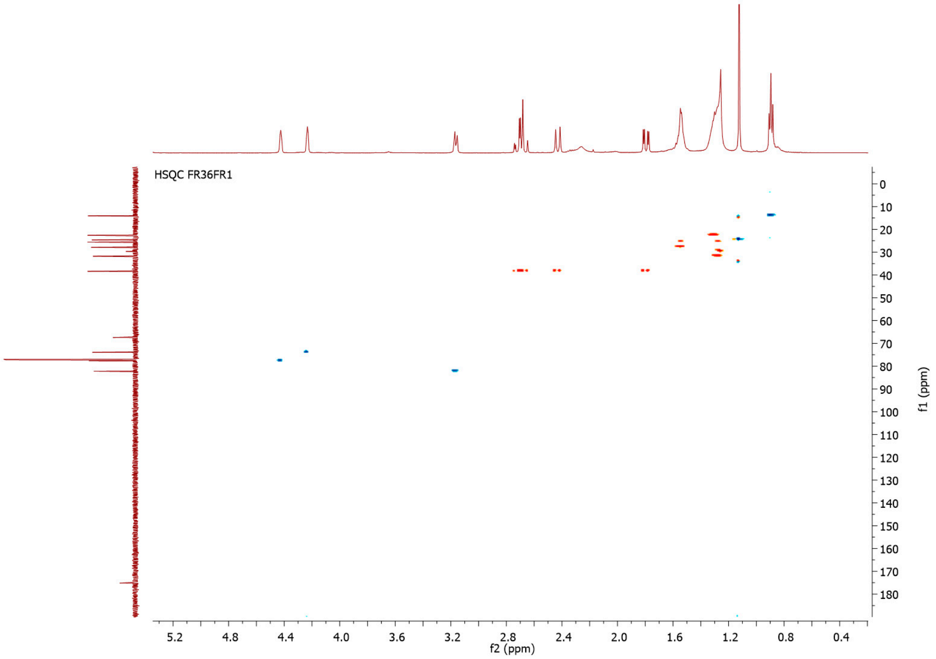


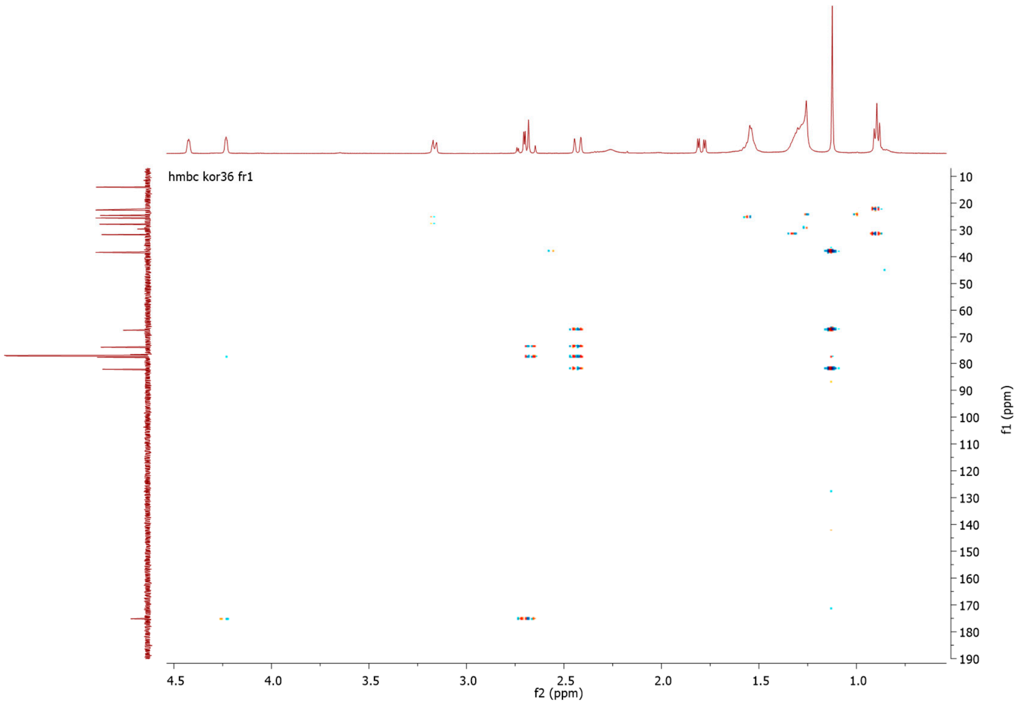


***Figure S32:*** $^{1}$H-$^{13}$C HSQC and $^{1}$H-$^{13}$C HMBC spectra for **29**

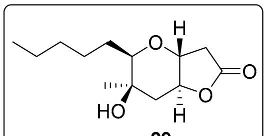

29

The compound **29** was eluted isocratically with methanol, with a retention time of 8.7 minutes. LC-MS analysis revealed a main peak with a relative area of 93.8 out of a total relative area of 100.0, corresponding to a calculated purity of 93.8%.

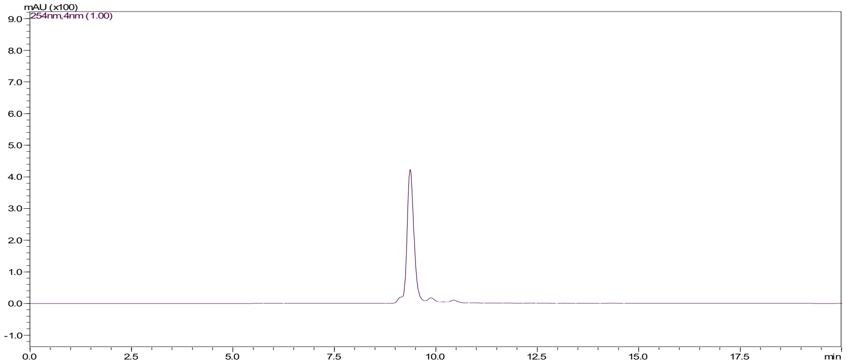


ESI-MS, positive mode: m/z calcd mass for $C_{15}H_{26}NO_4$ $[M+H+ACN]^+$ = 284.18, was found 285.00.

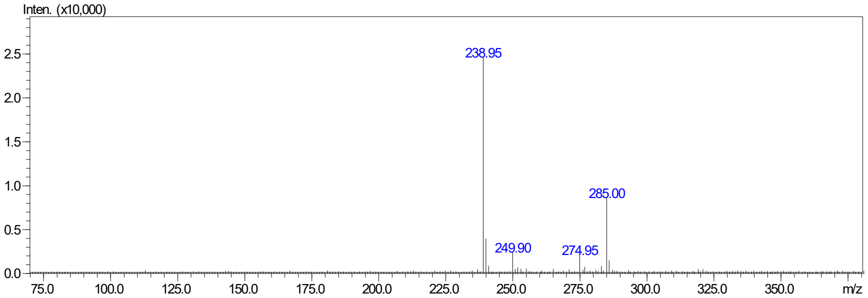


***Figure S33:*** ESI-LCMS for **29**

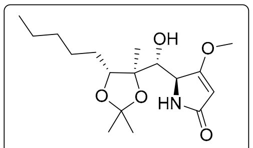


***syn*-30**

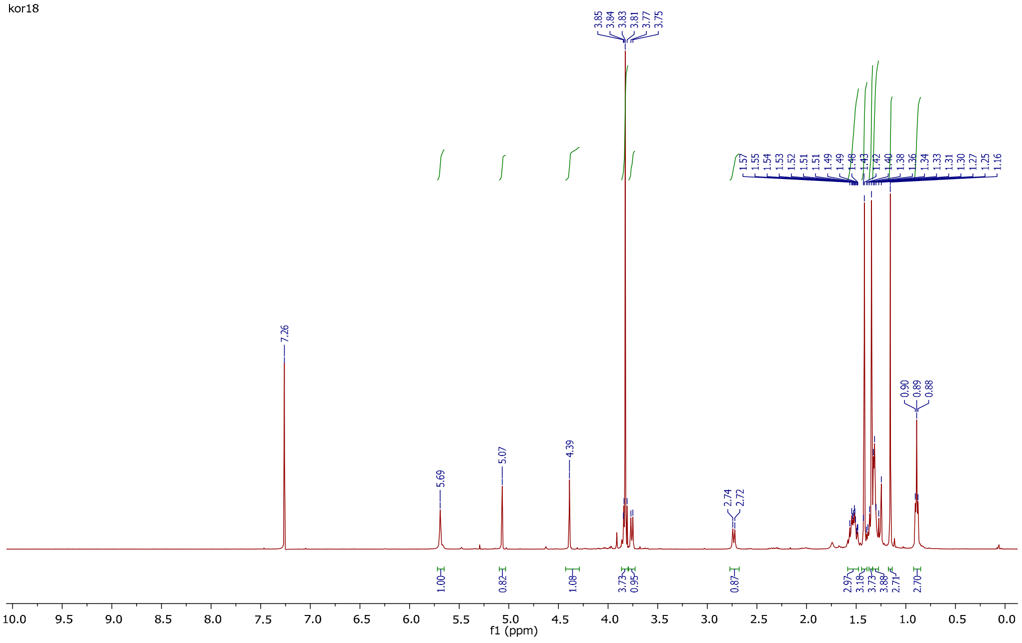


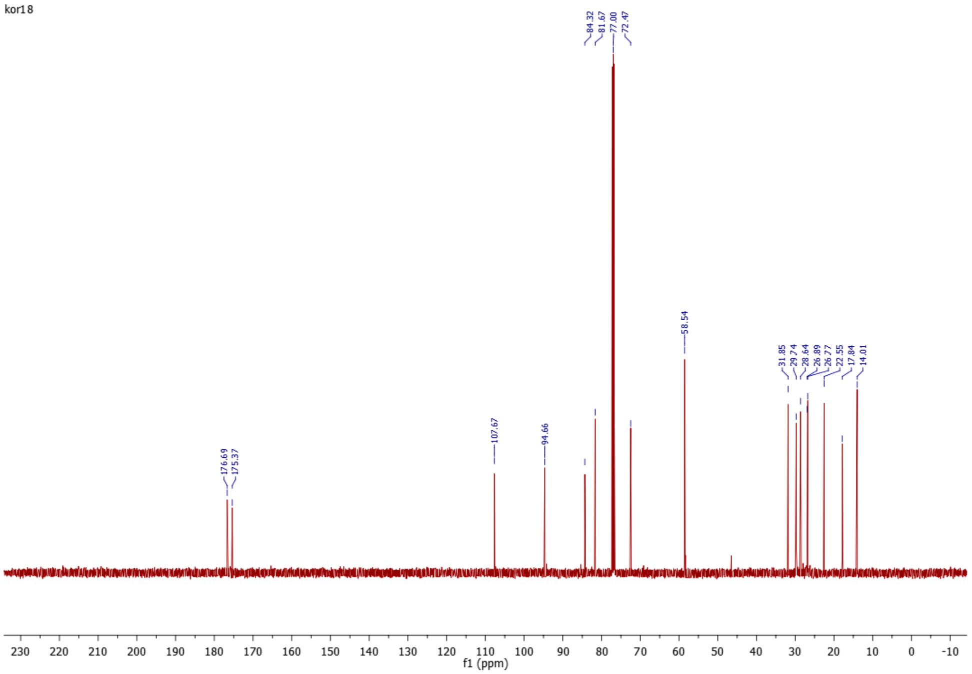


***Figure S34:*** $^{1}$H-NMR and $^{13}$C-NMR spectra for *syn*-**30**

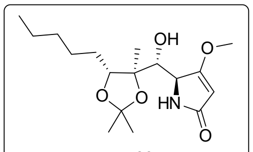

*syn*-**30**

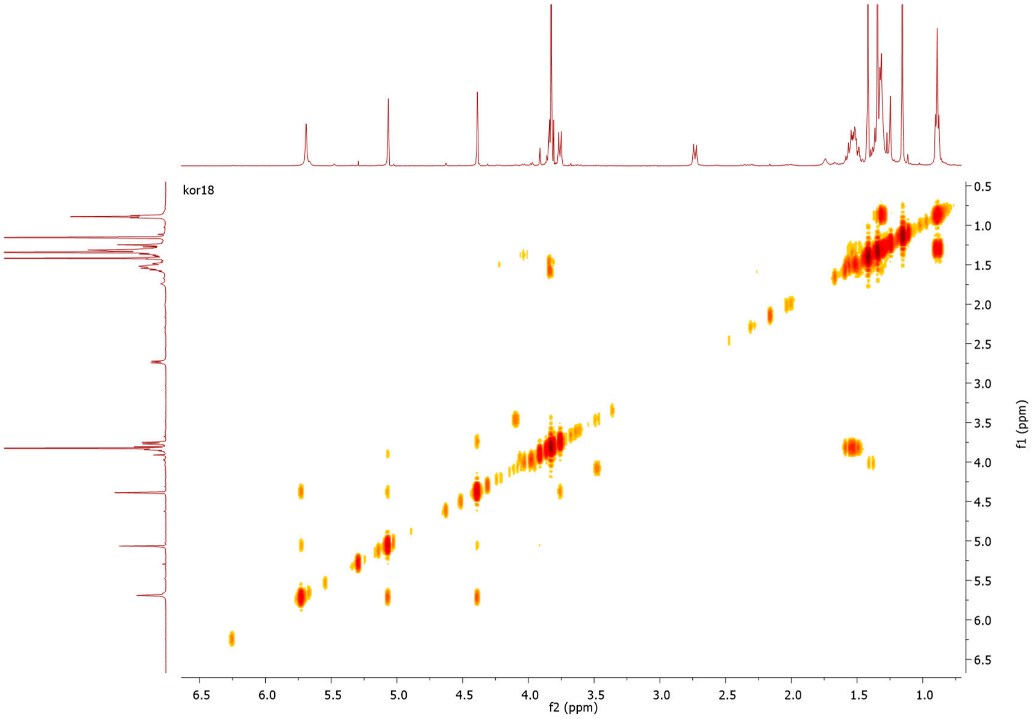


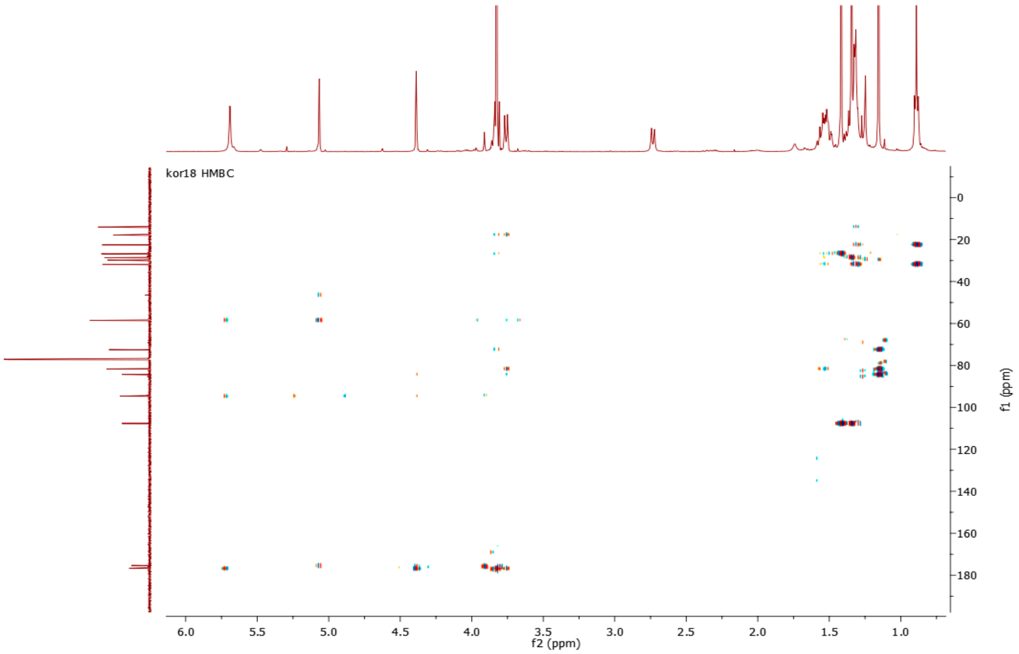


***Figure S35:*** $^{1}$H-$^{1}$H COSY and $^{1}$H-$^{13}$C HMBC spectra for *syn*-**30**

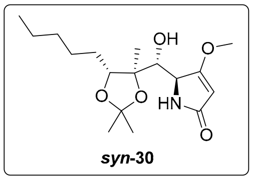


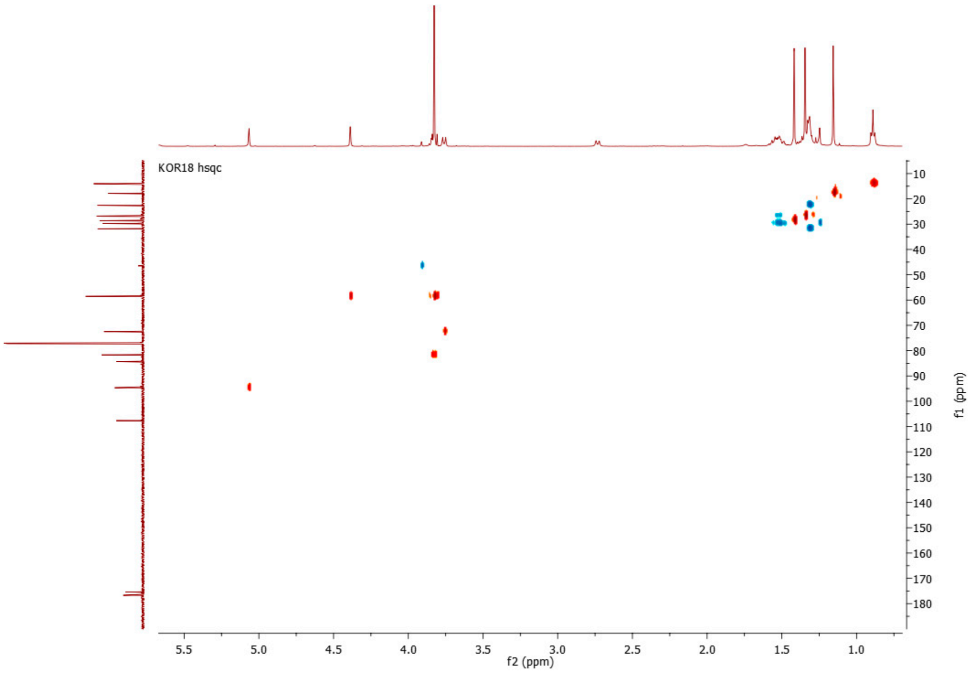


***Figure S36:*** $^{1}H$-$^{13}C$ HSQC spectrum for *syn*-**30**

ESI-MS, negative mode: m/z calcd mass for $C_{17}H_{28}NO_5$ $[M-H]^-$ = 326.1967, was found 326.05.

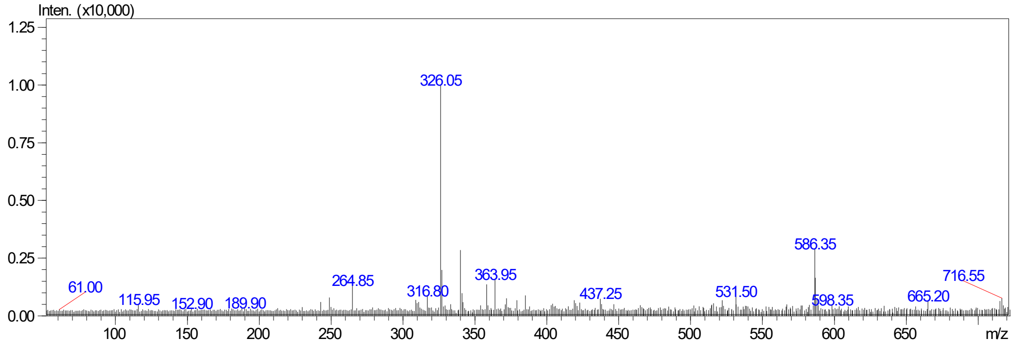


***Figure S37:*** MS analysis for *syn*-**30**

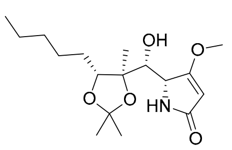


***anti*-30**

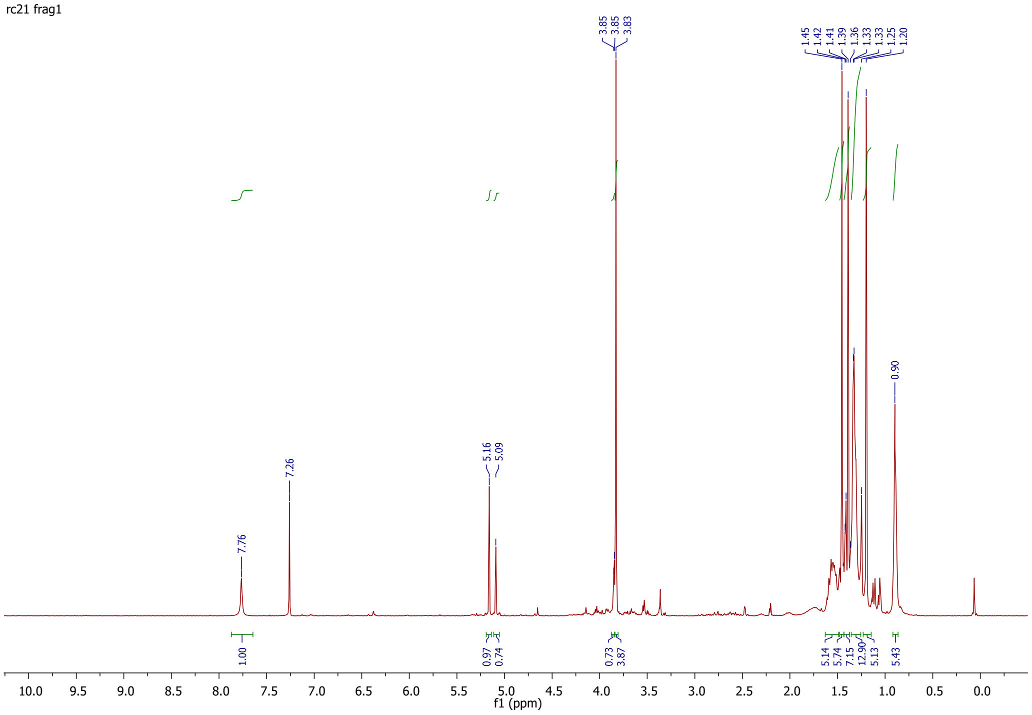


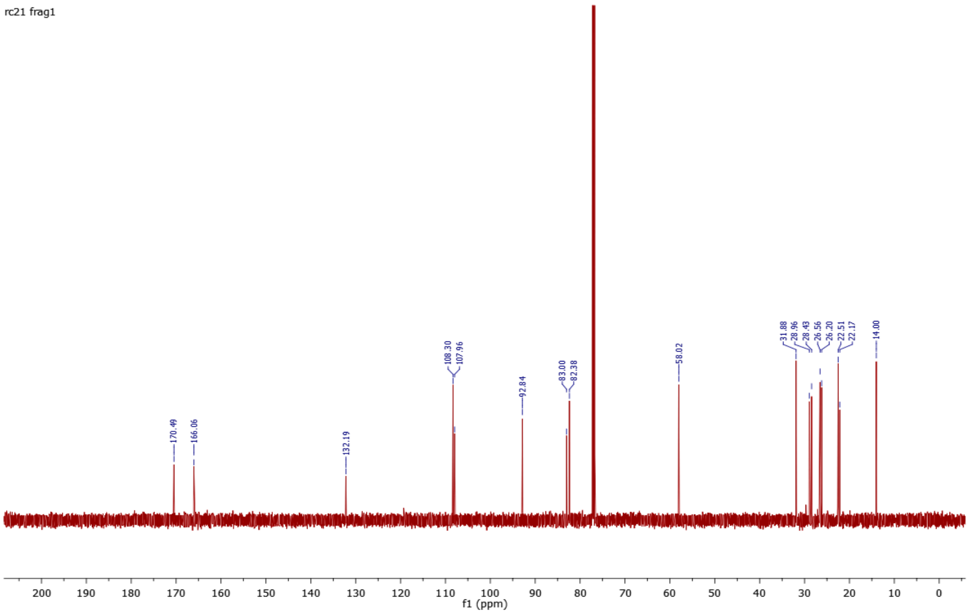


***Figure S38:*** $^{1}$H-NMR and $^{13}$C-NMR spectra for *anti*-**30**

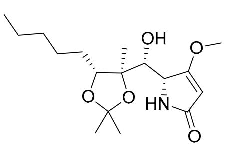


***anti*-30**

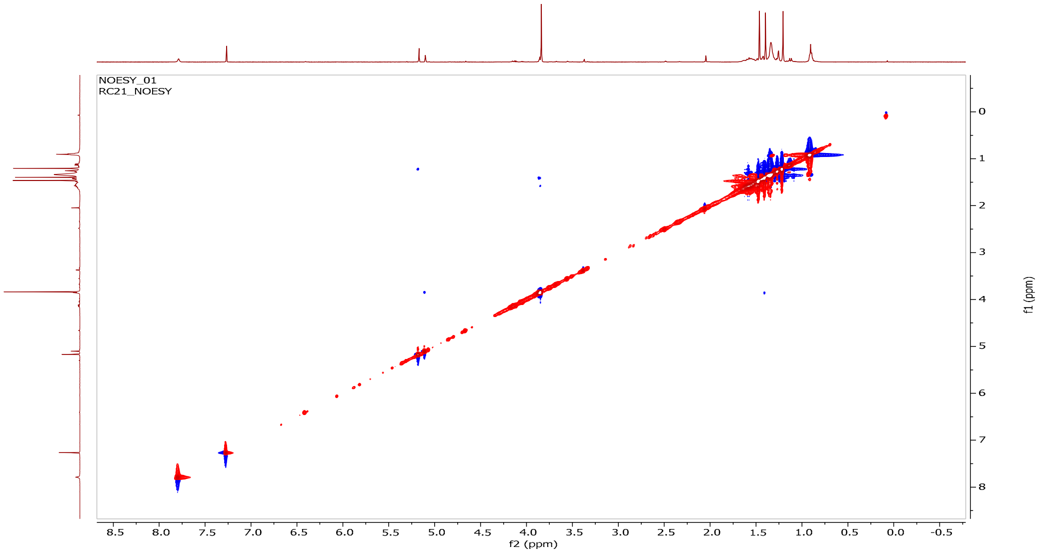


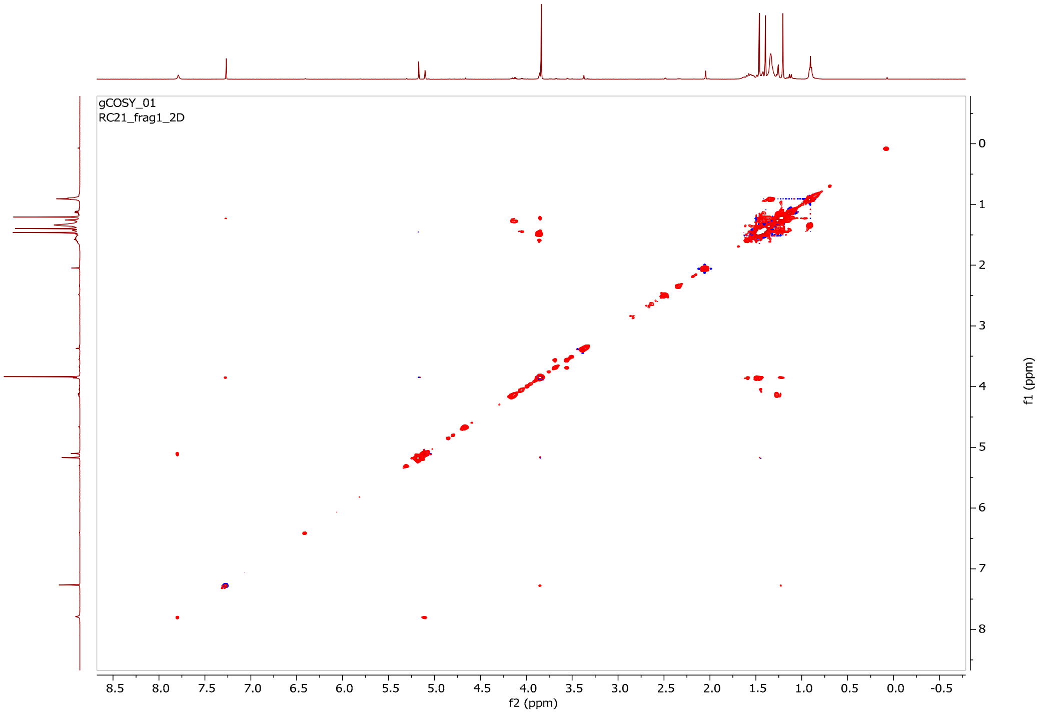


***Figure S39:*** $^{1}$H-$^{1}$H COSY and $^{1}$H-$^{1}$H NOESY spectra for *anti*-**30**

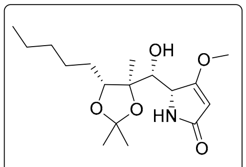


***anti*-30**

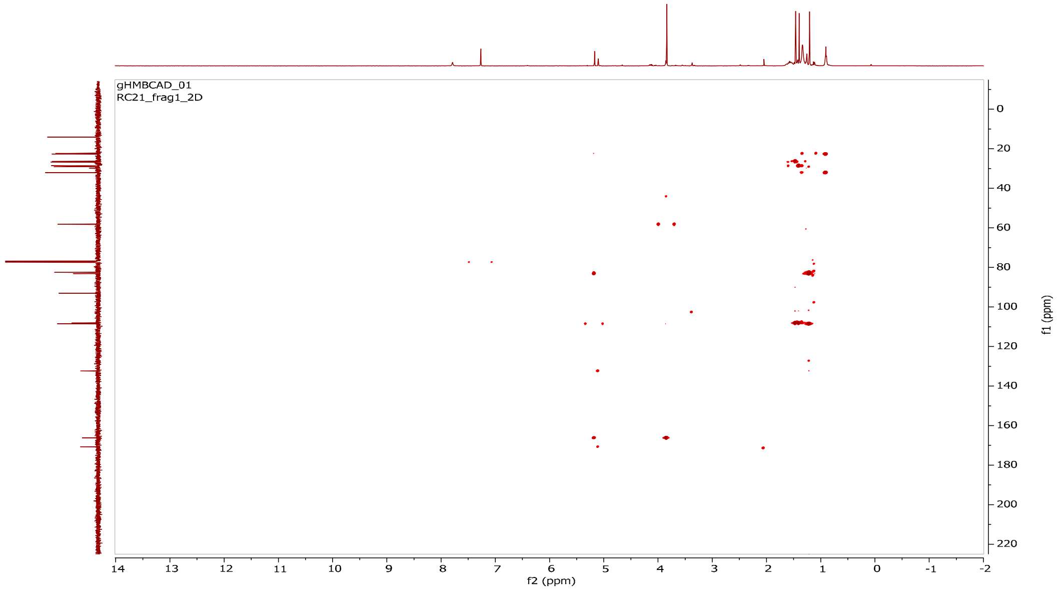


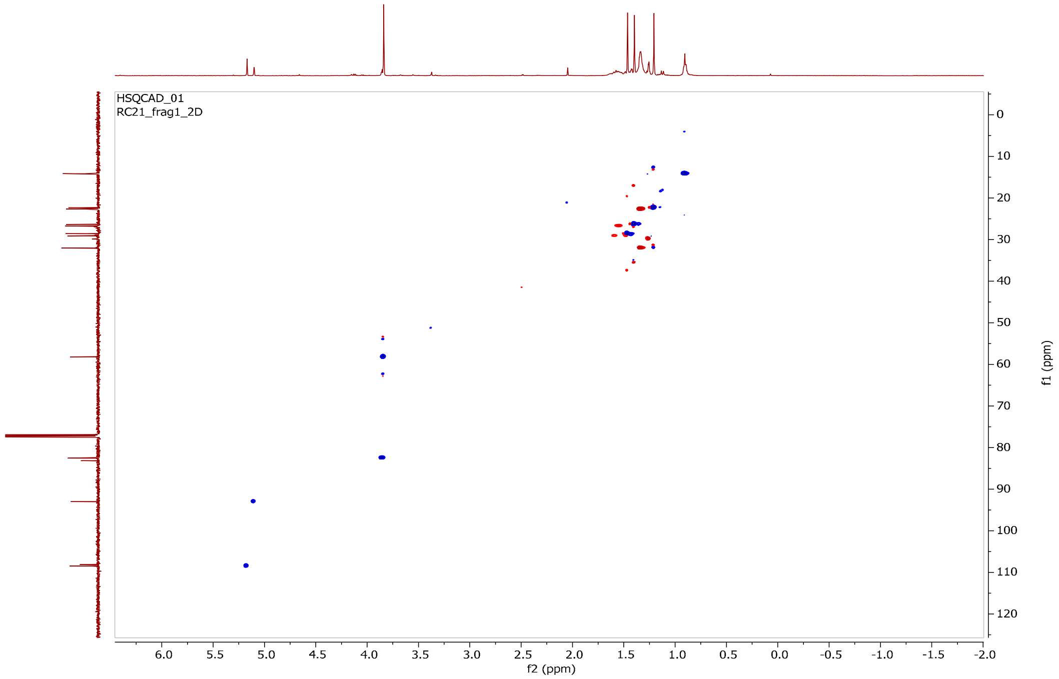


***Figure S40:*** $^{1}$H-$^{13}$C HMBC and $^{1}$H-$^{13}$C HSQC spectra for *anti*-**30**

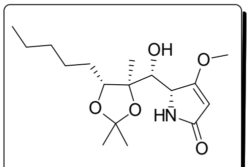


***anti*-30**

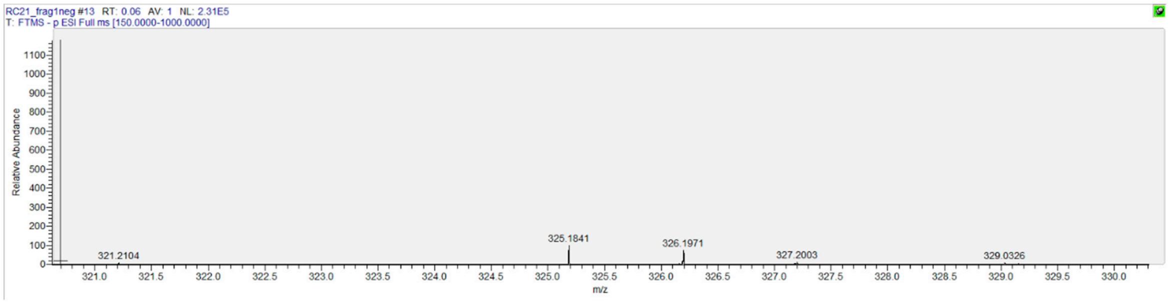


***Figure S41:*** HRMS analysis for *anti*-**30**

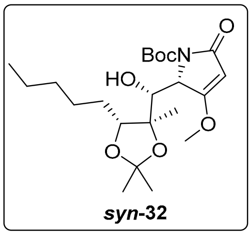


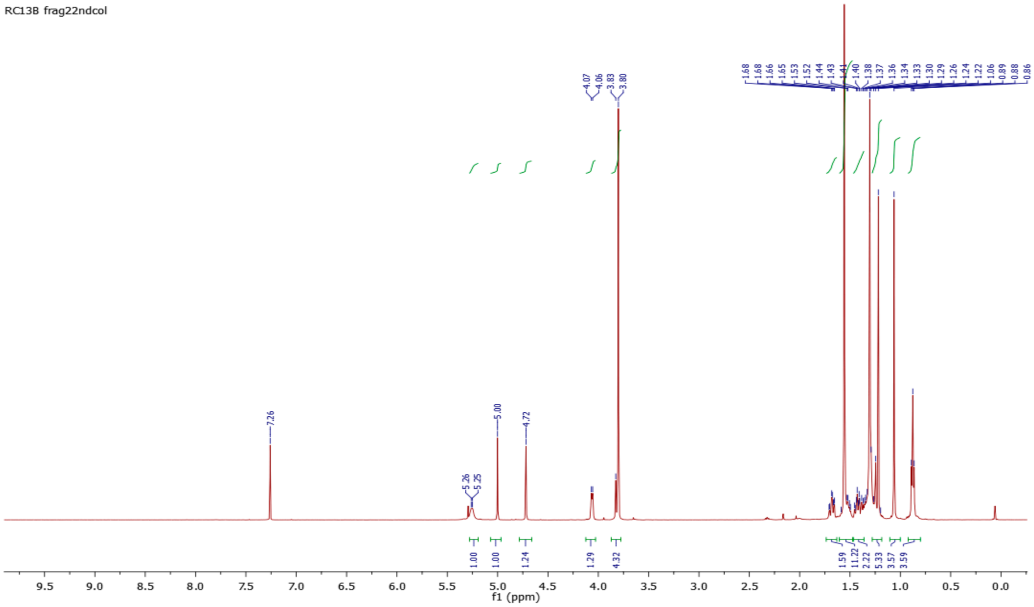


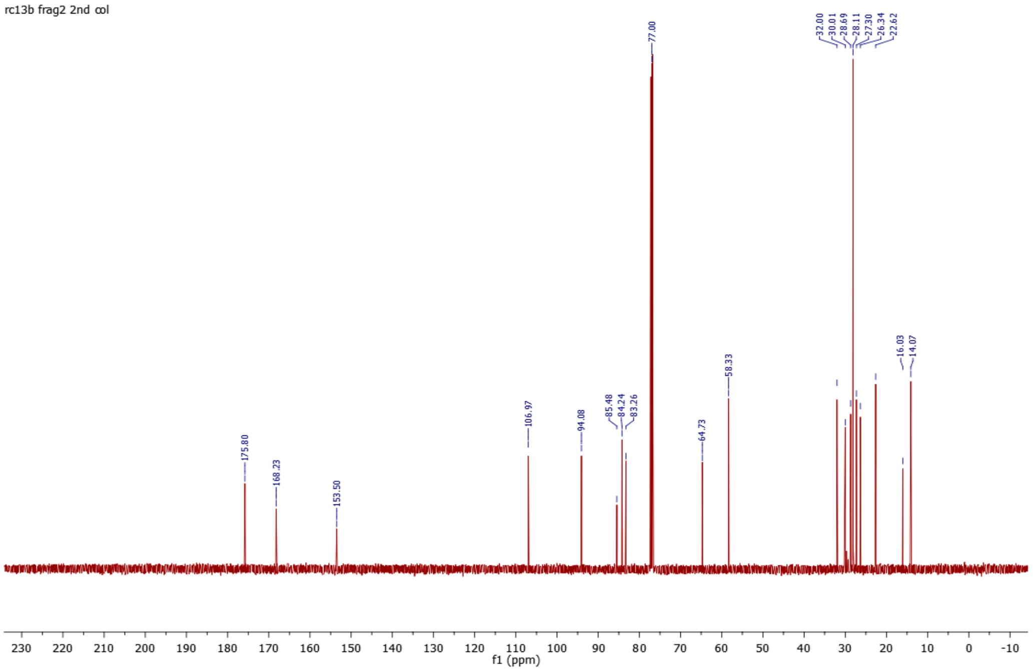


***Figure S42:*** $^{1}$H-NMR and $^{13}$C-NMR spectra for *syn*-**32**

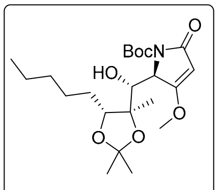


***anti*-32**

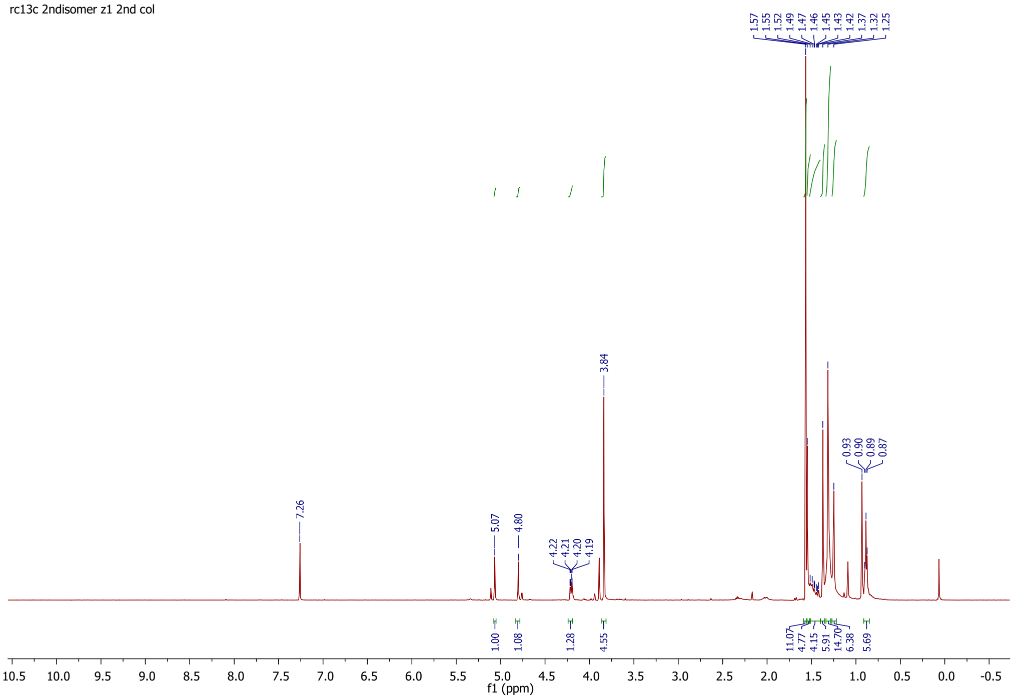


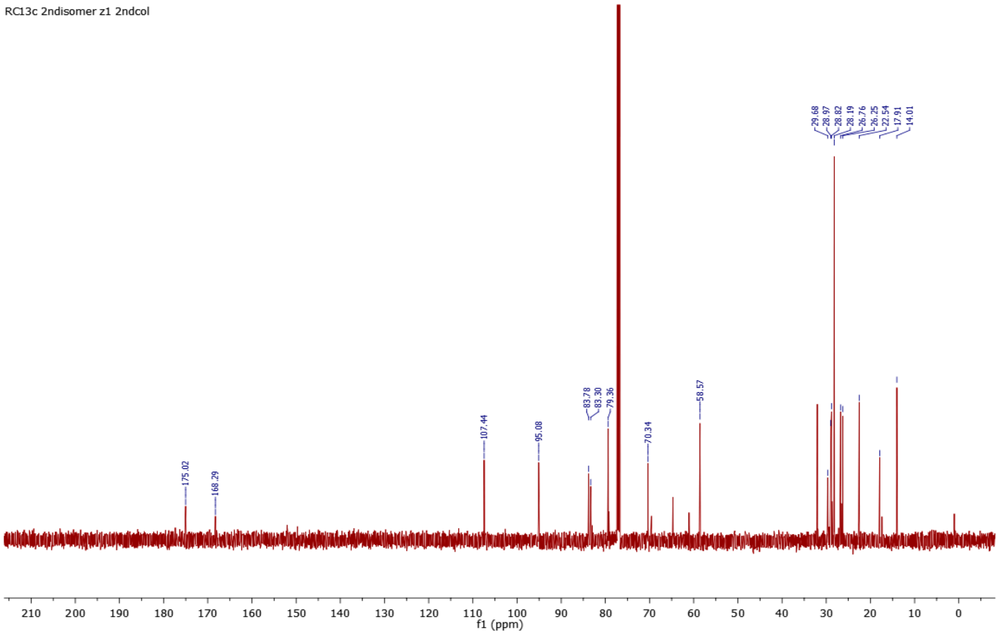


***Figure S43:*** $^{1}$H-NMR and $^{13}$C-NMR spectra for *anti*-**32**

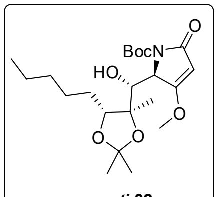

***anti*-32**

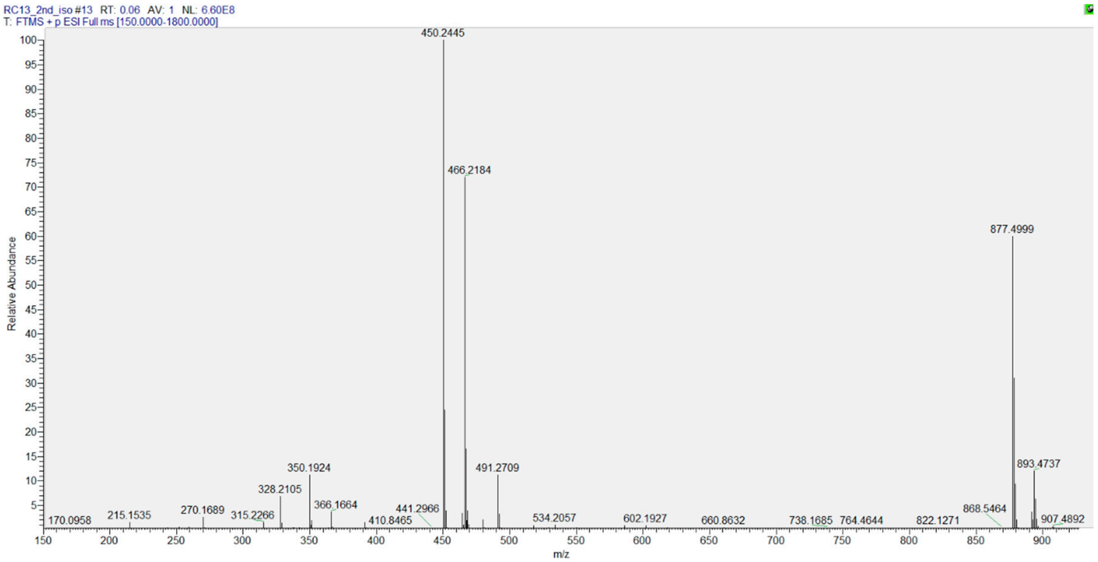


***Figure S44:*** HRMS analysis for *anti*-**32**

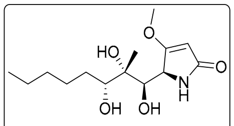


**17**

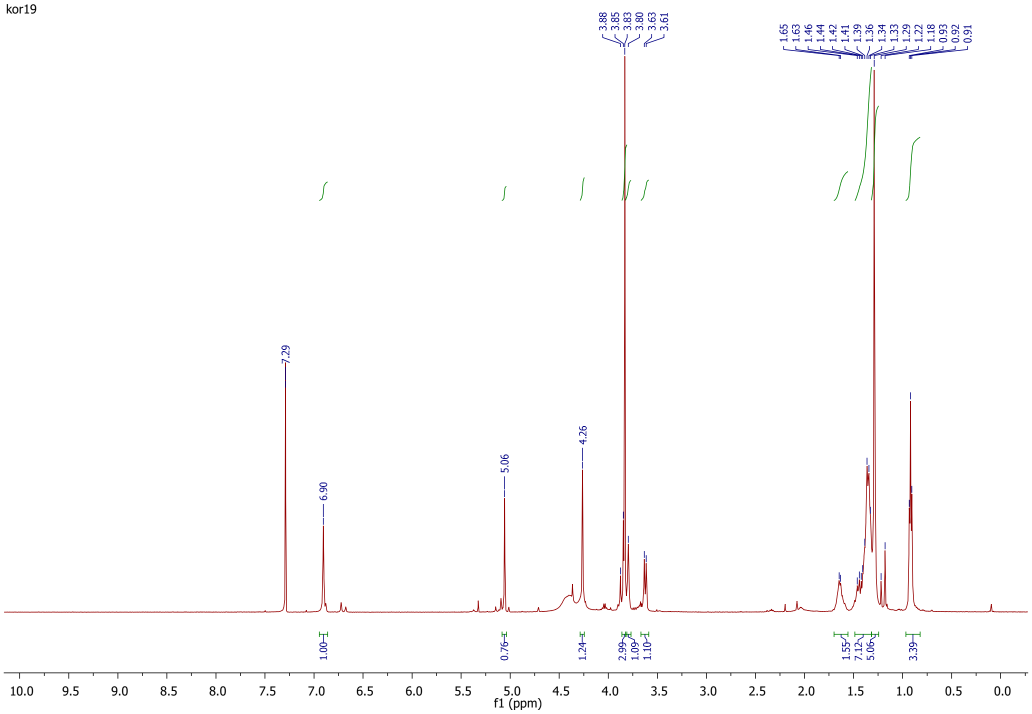


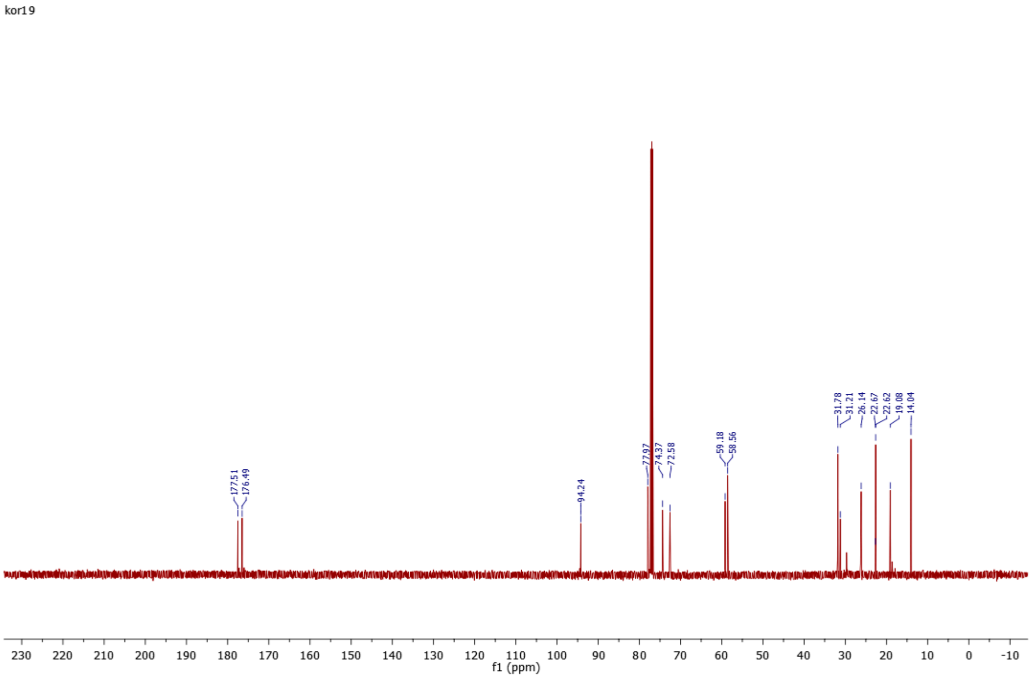


***Figure S45:*** $^{1}$H-NMR and $^{13}$C-NMR spectra for **17**

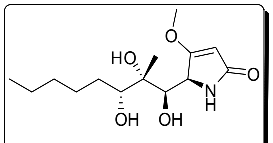


17

The compound **17** was eluted isocratically with methanol, with a retention time of 8.1 minutes. LC-MS analysis revealed a main peak with a relative area of 98.4 out of a total relative area of 100.0, corresponding to a calculated purity of 98.4%.

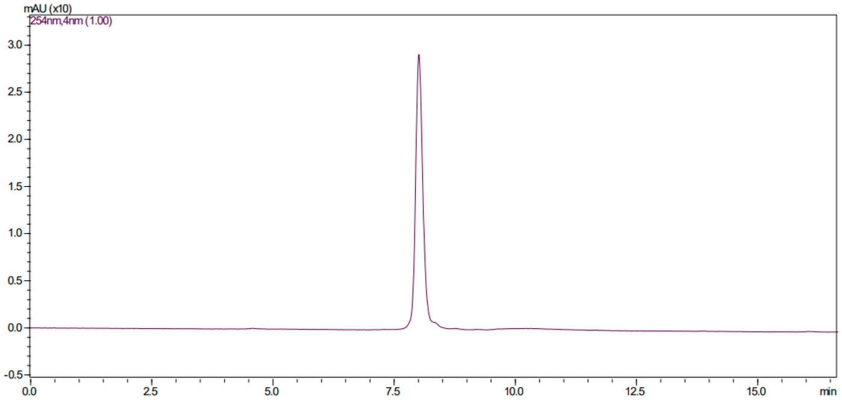


ESI-MS, positive mode: m/z calcd mass for $C_{14}H_{25}O_5$ $[M+Na]^+$ = 310.1630, was found 309.95.

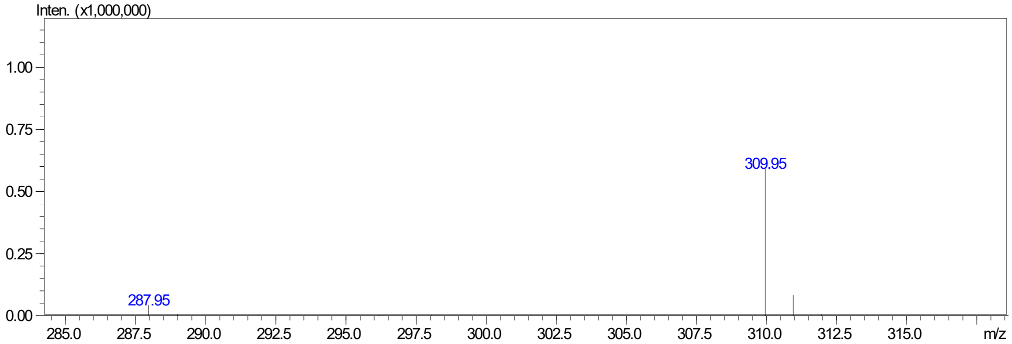


***Figure S46:*** ESI-LCMS analysis for **17**

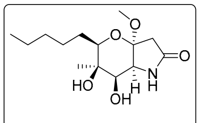

33a

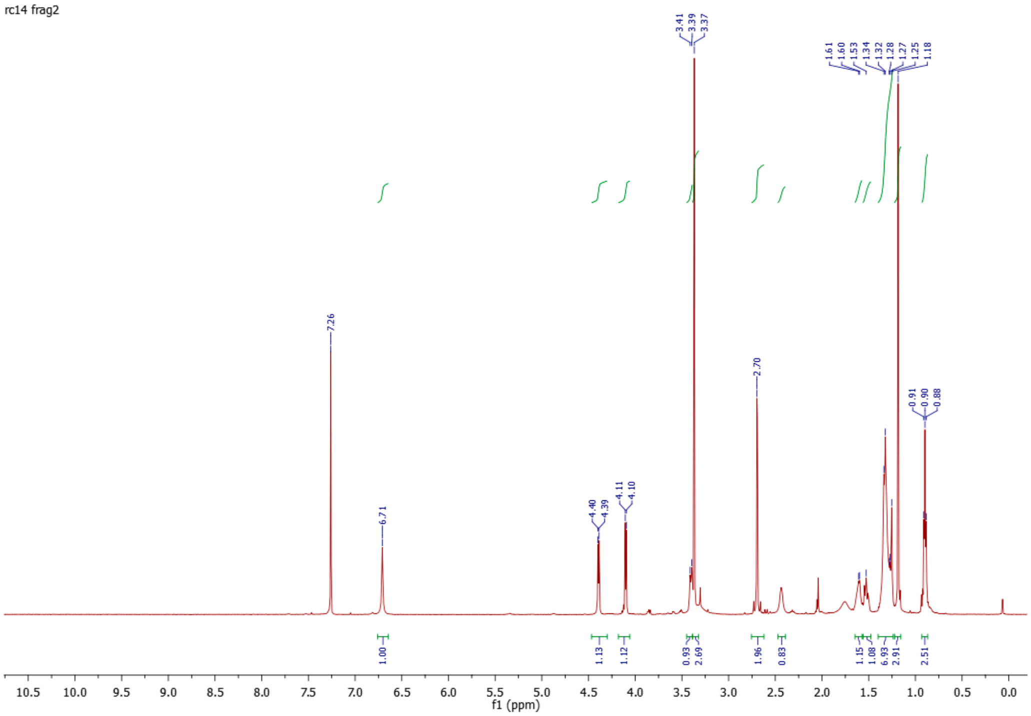


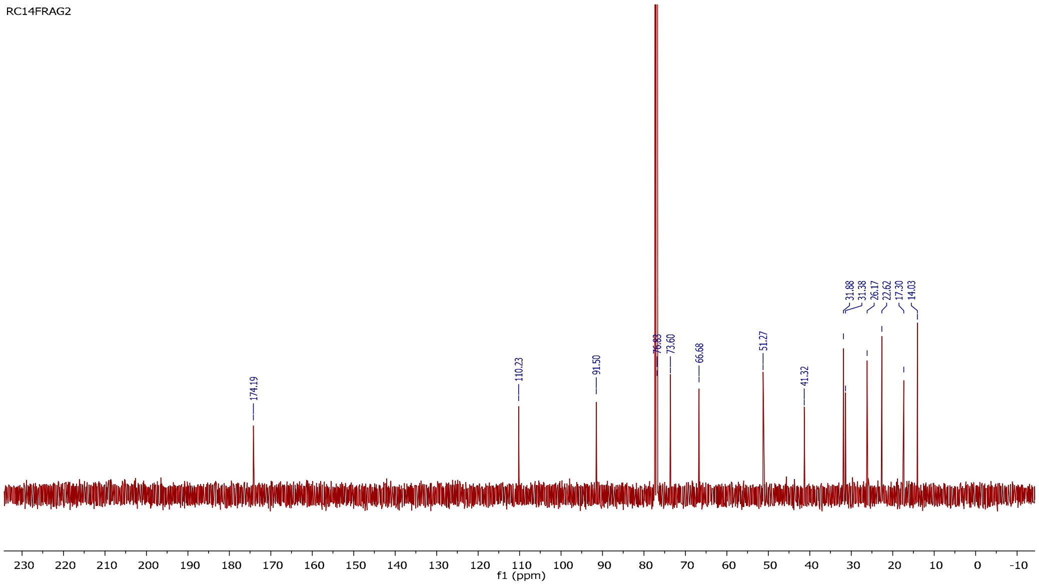


***Figure S47:*** $^{1}$H-NMR and $^{13}$C-NMR spectra for **33a**

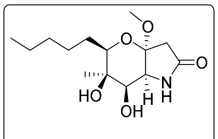

33a

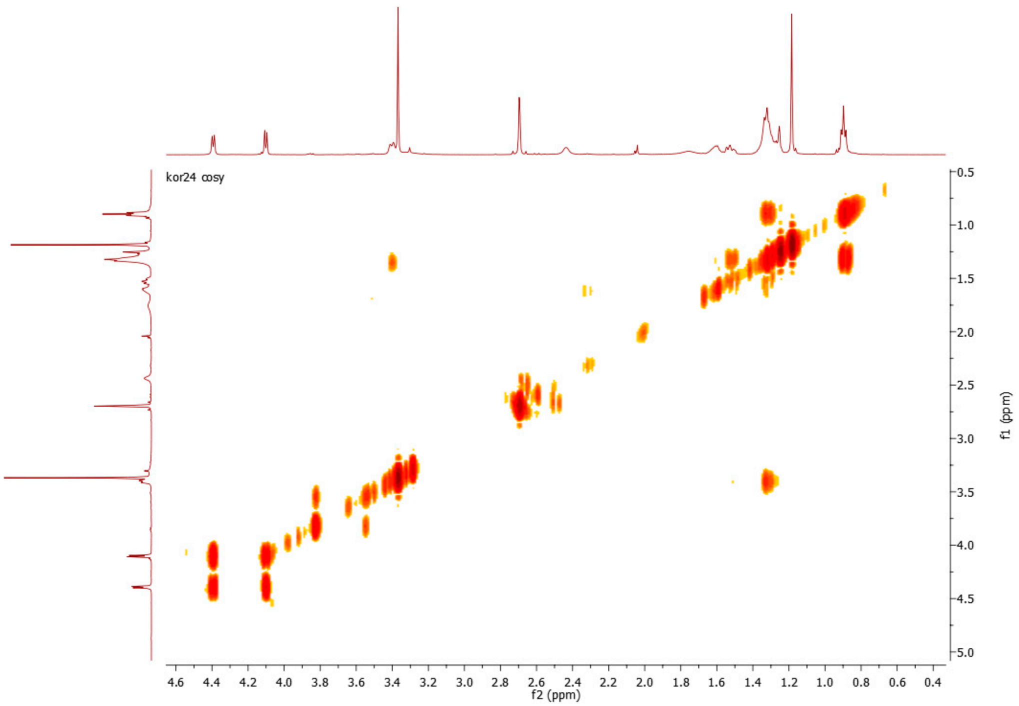


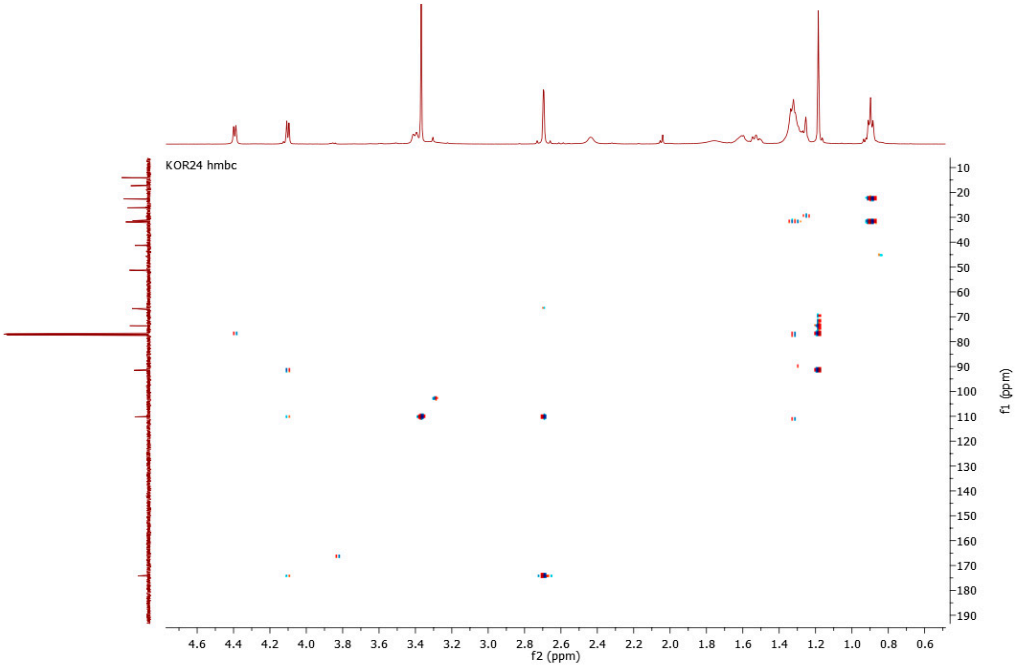


***Figure S48:*** $^{1}$H-$^{1}$H COSY spectrum and HMBC spectrum for **33a**

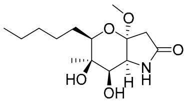


**33a**

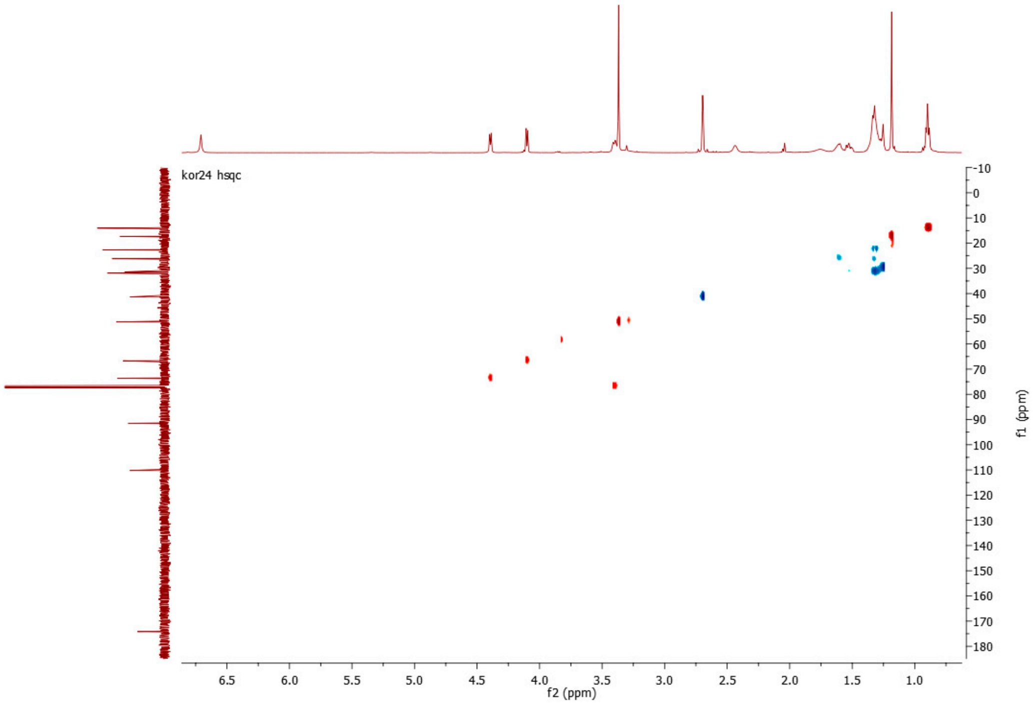


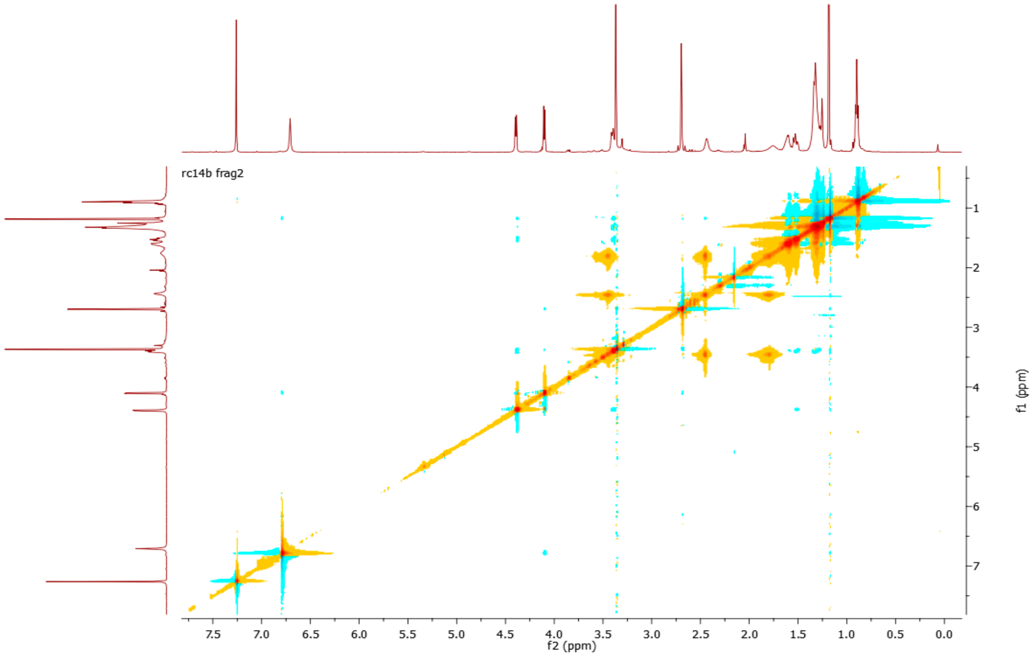


***Figure S49:*** $^{1}$H-$^{13}$C HSQC and $^{1}$H-$^{1}$H NOESY spectra for **33a**

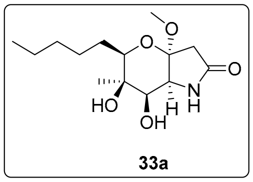


noesy

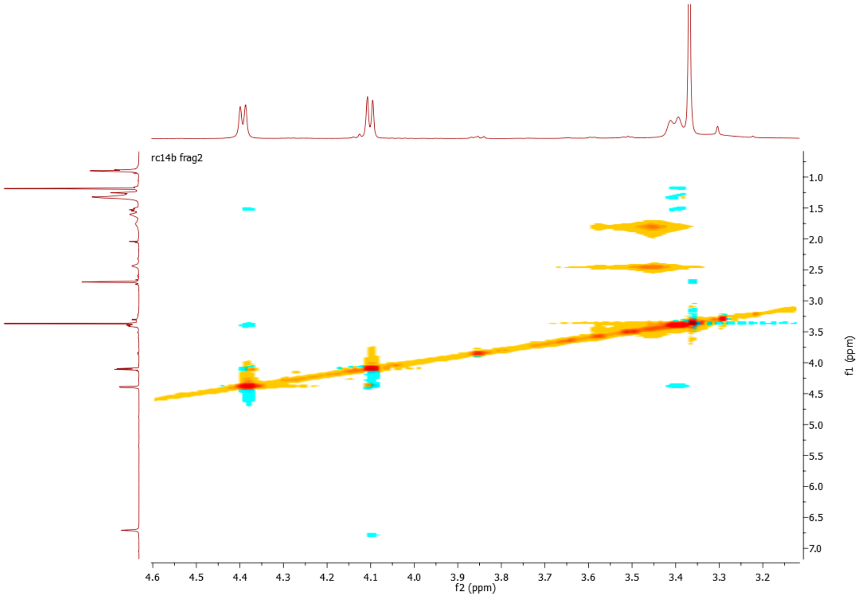


***Figure S50:*** $^{1}H$-$^{1}H$ NOESY spectrum for **33a**

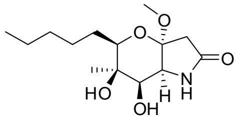

**33a**

The compound **33a** was eluted using a gradient system composed of water and acetonitrile, with a retention time of 20.7 minutes. LC-MS analysis revealed a main peak with a relative area of 92.7 out of a total relative area of 100.0, corresponding to a calculated purity of 92.7%. Early eluting peaks corresponding to solvent front and impurities were excluded from the integration.

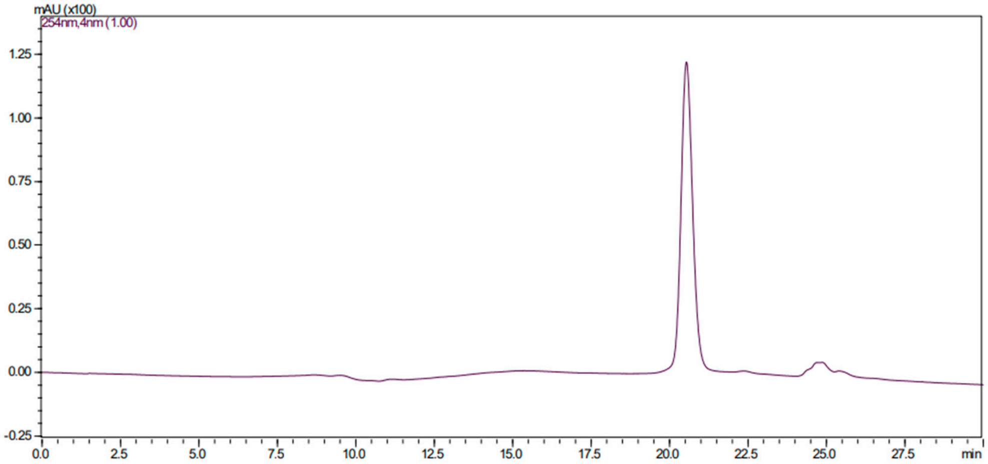


ESI-MS, positive mode: m/z calcd mass for $C_{14}H_{26}O_5$ $[M+H]^+$ = 288.1811, was found 287.90.

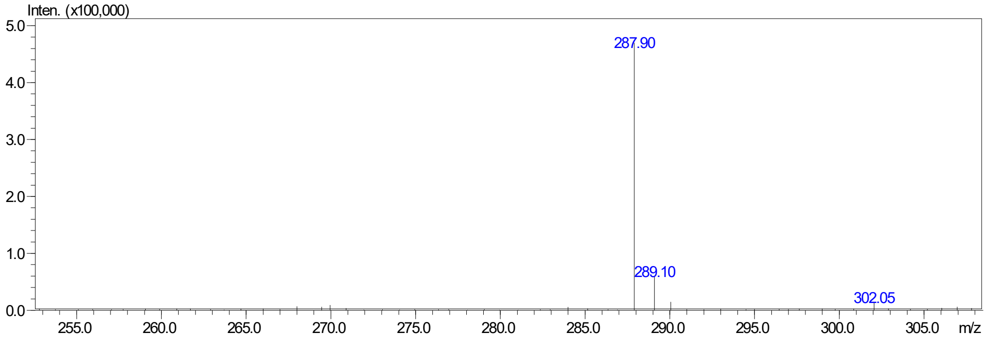


***Figure S51:*** ESI-LCMS analysis for **33a**

**HPLC-MS parameters and method development**

Chromatographic separation was performed using a Shimadzu LC-20AD system coupled to a Shimadzu LCMS-2010EV mass spectrometer. A Supelco Discovery C18 column (250 × 4.6 mm, 5 µm particle size) was employed for the separation. The mobile phase was delivered at a flow rate of 0.4 mL/min, and the column temperature was maintained at 26 °C. UV detection was carried out at 254 nm. The mass spectrometer was operated at an ionization voltage of 1.65 kV. All compounds, except for **33a**, were eluted isocratically using methanol as the mobile phase.

- Method for the elution of **33a**.

**Table S1**. Conditions for the elution of **33a**.

| time (min) | H2O (% v/v conc.) | ACN (% v/v conc.) |
|---|---|---|
| 3 | 90 | 10 |
| 22 | 15 | 85 |
| 25 | 10 | 90 |
| 26 | 10 | 90 |
